# The Gut Bacteria Dysbiosis Contributes to Chronic Graft-Versus-Host Disease Associated With a Treg/Th1 Ratio Imbalance

**DOI:** 10.3389/fmicb.2022.813576

**Published:** 2022-09-08

**Authors:** Yulian Wang, Lisi Huang, Tian Huang, Suxia Geng, Xiaomei Chen, Xin Huang, Peilong Lai, Xin Du, Jianyu Weng

**Affiliations:** Department of Hematology, Guangdong Provincial People’s Hospital, Guangdong Academy of Medical Sciences, Guangzhou, China

**Keywords:** chronic graft-versus-host disease, gut bacteria, dysbiosis, Treg, Th1

## Abstract

**Introduction:**

Dysbiosis of gut bacteria has been discovered in a large number of autoimmune diseases. However, the influence of the gut bacteria in the mice model of chronic sclerodermatous graft-versus-host disease (Scl-GVHD), a disease that resembles an autoimmune disease characterized by chronic inflammation of multiple organs, such as skin, remains elusive. Here, we explore the role of gut bacteria in an Scl-cGVHD mice model.

**Methods:**

We established a mouse model of Scl-cGVHD, collected fecal flora, analyzed the composition, and diversity of intestinal flora using 16S rDNA amplicon sequencing, and detected the proportion of Treg and Th1 cells in splenocytes of Scl-cGVHD mice. To verify the immunoregulatory effect of Scl-cGVHD intestinal flora, we prepared bacterial extracts, co-cultured with splenocytes *in vitro*, and used flow cytometry to detect T cell differentiation and cytokine secretion.

**Results:**

By examining T-cell differentiation in splenocytes of cGVHD mice, we found that Treg cells were significantly reduced (15.27 ± 0.23 vs. 12.23 ± 0.47, *p* = 0.0045) and Th1 cells were increased (1.54 ± 0.18 vs. 6.68 ± 0.80, *p* = 0.0034) in cGVHD mice. Significant differences were observed in the composition and diversity of the gut bacteria in mice with Scl-cGVHD versus without GVHD. Analysis of mice fecal bacteria samples (*n* = 10, 5 Scl-cGVHD and 5 Non-GVHD) showed significant separation [*R* = 0.732, *p* = 0.015, non-parametric analysis (ANOSIM)] in Scl-cGVHD and non-GVHD mice. The abundance of the family and genus *Ruminococcaceae* bacteria decreased and the family *Lachnospiraceae* and limited to the species *Lachnospiraceae_bacterium_DW17* increased in Scl-cGVHD mice. *In vitro* results of the cellular level study suggest that the bacteria extracts of gut microbiota from Scl-cGVHD mice modulated the splenic T cells toward differentiation into CD4^+^IFN-γ^+^ Th1 cells (14.37 ± 0.32 vs. 10.40 ± 2.19, *p* = 0.036), and the percentage of CD4^+^CD25^+^Foxp3^+^ Tregs decreased (6.36 ± 0.39 vs. 8.66 ± 0.07, *p* = 0.001) compared with the non-GVHD mice. In addition, the secretion of proinflammatory interferon- γ (IFN-γ) cytokine in the supplement of cellular culture was increased (4,898.58 ± 235.82 vs. 4,347.87 ± 220.02 pg/ml, *p* = 0.042) in the mice model of the Scl-cGVHD group, but anti-inflammatory interleukin (IL)-10 decreased (7,636.57 ± 608.05 vs. 9,563.56 ± 603.34 pg/ml, *p* = 0.018).

**Conclusion:**

Our data showed the different composition and diversity of gut bacteria in the Scl-cGVHD mice. The dysbiosis of gut bacteria may regulate the differentiation ratio of Treg and Th1 cells, which was associated with Scl-cGVHD.

## Introduction

Graft-versus-host disease (GVHD) is a life-threatening complication characterized by multi-organ dysfunction due to immune dysregulation after allogeneic hematopoietic stem cell transplantation (allo-HSCT) (Im et al., 2016; Annalisa et al., 2018; Zeiser et al., 2021), which is a potentially curative treatment for hematologic malignancies. According to the clinic manifestations and the time of occurrence, GVHD was classified as acute GVHD (aGVHD) and chronic GVHD (cGVHD), which the former inducing acute inflammatory reaction and the latter mediates the fibrotic pathological damage of various tissues and organs throughout the whole body (Ferrara et al., 2009; Min, 2011). Although the pathogenesis of cGVHD remained to be somewhat ill-defined, there were several T-helper lineages associated with the targeted tissue damage in chronic GVHD (Sarantopoulos et al., 2015; Jin et al., 2016). Th1 cells were reported to be the predominant cytotoxic effectors in sclerodermatous cGVHD (Im et al., 2016; Lai et al., 2018). Treg cells have been demonstrated to maintain the balance between autoimmunity and tolerance by suppressing the proliferation and effector functions of T, B, NK cells, and antigen presenting cells (APCs) (Sakaguchi et al., 2008; Kumar et al., 2019). A reduced percentage of Treg cells was shown to induce the development of cGVHD (Koreth et al., 2011; Du et al., 2017). Furthermore, in our previous studies, we also found that the therapy targeted to increase the ratio of the number of Treg cells and decrease the differentiation of T-helper lineages cells was shown to be effective in alleviating sclerodermatous cGVHD (Wang et al., 2017; Chen et al., 2018). Some factors have been reported to induce the pathophysiology of cGVHD, such as HLA-type, conditioning regimen, and the prolongation of aGVHD (Ringdén et al., 2017; Santoro et al., 2018; Saraceni et al., 2019). Recently, the dysbiosis of gut microbiota was demonstrated to play an important role in the development of acute gastrointestinal graft-versus-host disease (Mathewson et al., 2016; Peled et al., 2016) and a variety of autoimmune diseases, such as multiple sclerosis (MS) (Jangi et al., 2016), inflammatory bowel disease (IBD) (Chu et al., 2016), type 1 diabetes (Forbes et al., 2018), rheumatoid arthritis (RA) (Vatanen et al., 2018), systemic lupus erythematosus (SLE) (Luo et al., 2018) *via* modulating the differentiation of T-helper lineages in human and mice.

In multiple sclerosis, *Akkermansia muciniphila* and *Acinetobacter calcoaceticus* increased in patients with MS, which induced proinflammatory responses that revealed a pronounced effect on stimulating Th1 differentiation, but *Parabacteroides distasonis* stimulated anti-inflammatory Treg cells was reduced in patients with MS (Cekanaviciute et al., 2017). In the mice model of SLE, a decrease in *Lactobacillaceae* and an increase in *Lachnospiraceae* were observed. In patients with SLE, the relative abundance of *Lachnospiraceae* was significantly greater. In the aGVHD mice model, dysbiosis including decreased *Clostridiales* and increased *Enterobacteriales* was observed (Jenq et al., 2012; Simms-Waldrip et al., 2017). In human aGVHD, a previous retrospective study has indicated that the relative abundance of *Lachnospiraceae* and *Ruminococcaceae* was positively correlated to the percentage of Treg cells, influencing the development of acute gut GVHD (Han et al., 2018). Accordingly, a large number of gut bacteria species have been demonstrated to have immunomodulatory effects on the differentiation of T-helper lineages, which are devoted to the pathogenesis of cGVHD. The gut microbiota played a vital role in the development of aGVHD, but whether gut bacteria differ in chronic sclerodermatous GVHD versus non-GVHD individuals has not been reported. Taken together, we analyzed the relative abundance of gut microbiota in the mice model of chronic sclerodermatous GVHD and the immunoregulation of microbiota on the differentiation of Treg and Th1 cells.

## Materials and Methods

### Chronic Sclerodermatous Graft-Versus-Host Disease Mice Model

The male B10.D2 mice (10–12 weeks old, Jackson Laboratory) and female BALB/cJ mice (10–12 weeks old, Beijing Vital River Laboratory Animal Technology Co., Ltd.) were used to establish the mice model of sclerodermatous cGVHD that could simulate Scl-GVHD and systemic sclerosis in humans as described previously (Wang et al., 2017; Lai et al., 2018). Briefly, recipient female BALB/cJ mice received total body irradiation with 700 cGy Cs137 ray and were transplanted by tail vein injection with 8 × 10^6^ bone marrow cells and 8 × 10^6^ spleen cells from B10.D2 mice (1:1, Scl-cGVHD mice model; 1:0, non-GVHD). The clinical score, body weight loss, and activities of the recipient mice were monitored every 3 days beginning at Day 14 after bone marrow transplantation (BMT). Chronic sclerodermatous GVHD was assessed using an established system as reported previously (Lai et al., 2018). All experiments used on mice in this study were approved by the animal experimental ethics committee of Guangdong Provincial People’s Hospital.

### Hematoxylin-Eosin Staining of Skin Tissues

Skin tissues from mice were collected and fixed in 10% formaldehyde and then embedded in paraffin. Hematoxylin-Eosin (HE) staining was conducted to examine histopathological changes in the skin. The results of the HE staining were observed by light microscopy.

### Microbiota Sampling, DNA Extraction and Sequencing, and Metagenome Analysis

Fecal pellets were collected from chronic sclerodermatous GVHD or non-GVHD mice 35 days after bone marrow transplantation. All fecal pellets were stored at –80^°^C until they were processed for DNA extraction and sequencing. The fecal bacterial genomic DNA was extracted by cetyltrimethylammonium bromide (CTAB) or sodium dodecyl sulfate (SDS) method, and the purity and concentration of the DNA were detected by agarose gel electrophoresis. The appropriate amount of fecal bacterial genomic DNA was diluted to 1 ng/μl with sterile water (*n* = 5 per group). PCR amplification was performed using the diluted genomic DNA as a template and 16S V4 region primers, the forward primer: GTGCCAGCMGCCGCGGTAA, and the reverse primer: GGACTACHVGGGTWTCTAAT. Phusion^®^ High-Fidelity PCR Master Mix with GC Buffer and high-efficiency high-fidelity enzyme. The intestinal flora DNA library was constructed by using the Thermofisher’s Ion Plus Fragment Library Kit 48 rxns library kit. The constructed library was sequenced using the Thermo Fisher’s Ion S5TMXL by Novogene bioinformatics Technology Co., Ltd. Uparse software was used to cluster all clean reads of all samples. By default, the sequence is clustered into Operational Taxonomic Units (OTUs) with 97% identity. At the same time, the representative sequence of OTUs is selected according to the algorithm principle. The most frequently occurring sequences in OTUs were screened as representative sequences of OTUs. The UniFrac distance was calculated with Qiime software (Version 1.9.1). Diluted curves, beta diversity index analysis, principal component analysis (PCA), and principal coordinates analysis (PCoA) diagram drawing were performed by R software (Version 2.15.3). Analysis of similarities (Anosim) analysis was used by the R vegan package. The LEfSe analysis was analyzed with LEfSe software.

### Bacterial Extracts Preparation

We prepared bacterial extracts using an experimental approach based on a report by Cekanaviciute E. (Cekanaviciute et al., 2017). Briefly, total microbiota were isolated from cGVHD and non-GVHD mice stool samples by suspending 1–2 mg stool sample in 3 ml of PBS, removing the precipitate at low-speed centrifugation (40 g, 3 min), passing the supernatant through a 40 μm cell strainer and washing with 1.5 ml of PBS for two times by centrifuging at 8,000 rpm. Isolated bacteria were heat-inactivated at 65^°^C for 1 h, lysed with protease inhibitor and phosphatase inhibitor, and sonicated at 4^°^C for 2–3 min. Extracted bacteria protein concentrations were measured with the BCA protein assay kit.

### Mice Splenocytes Isolation, Cultures, and Activation

The splenocytes were isolated from female BALB/cJ mice and cultured in RPMI-1640 (Gibco) supplemented with 10% fetal bovine serum (FBS, Gibco), 1% penicillin/streptomycin (Sigma) at 37°C with 5% CO_2_. The splenocytes were counted and planked at 1 × 10^6^ cells/ml concentration and activated with IL-2 (40 ng/ml), anti-CD3 (5μg/ml), and anti-CD28 (2μg/ml) for 5 days, in the presence of bacterial extracts (1μg/ml) from Scl-cGVHD mice or non-GVHD mice.

### Flow Cytometry for Splenocytes

The Th1 and Treg cells were determined by CD4^+^IFNγ^+^, CD4^+^CD25^+^Foxp3^+^. The following antibodies were used to stain mice splenocytes: anti-CD4-FITC (eBioscience), anti-IFN-γ-APC (eBioscience), anti-CD25-APC (eBioscience), and anti-Foxp3-PE (eBioscience). Transcription factors Foxp3 were stained with antibodies after being formulated by the Foxp3/transcription factor staining buffer set. For interferon- γ (IFN-γ) detection, cells were cultured with a 1 × cell stimulation cocktail (plus protein transport inhibitors) for 4–6 h, fixed and permeabilized with a fixation and permeabilization buffer before staining with antibodies. Flow cytometry was performed on a BD CantoII cell analyzer and analyzed by FlowJo software (TreeStar). The percentage of Th1 and Treg cells was defined as Th1 and Treg cells within the CD4^+^ T cells.

### Cytokines Analysis

The proinflammatory cytokine IFN-γ and the immunoregulatory mediators IL-10 levels were detected using the BD™ Cytometry Bead Array kit.

### Statistical Analysis of *in vitro* Data

Statistical significance of expression of CD4^+^ T lymphocyte differentiation was determined using a two-tailed Student’s *t*-test. If the data were non-normally distributed, a two-tailed non-parametric Mann–Whitney test was applied instead. The percentage of Th1 and Treg cells was analyzed with FlowJo. SPSS 20.0 was used to analyze and GraphPad Prism 6 software was applied to plot the data. A *p*-value of < *0.05* was determined to be statistically significant.

## Results

### Successful Establishment of Sclerodermatous Chronic Graft-Versus-Host Disease Mice Model

**FIGURE 1:**
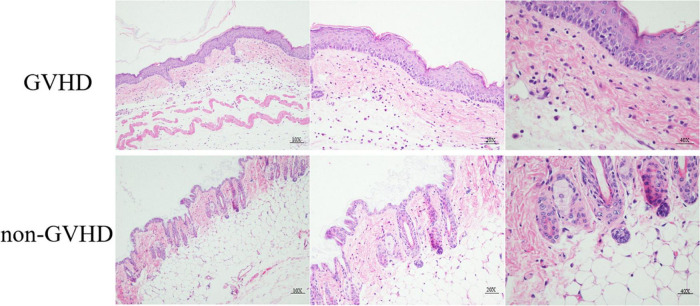
Histopathological HE staining analysis of skin from sclerodermatous chronic graft-versus-host disease (cGVHD) mice. Compared with the control group, the pathological damage to the skin in cGVHD mice was mainly manifested by obvious hyperplasia of the epidermis, massive collagen deposition in the dermis, extensive lymphocyte infiltration, severe subsclerodermatous fat atrophy, and a significant decrease in the number of hair follicles.

To analyze the role of gut microbiota and the relationship with the differentiation of Treg and Th1 cells on the sclerodermatous cGVHD, we established the mice model of sclerodermatous cGVHD (B10D2^H2d^♂→BALB/cJ^H2d^♀). The clinical manifestation of the sclerodermatous cGVHD mice is characterized by ulceration, escharotic, depilation, and sclerosis. Skin histopathological analysis with HE staining showed that the epidermal layer thickened, subsclerodermatous fat decreased, collagen deposited, a large number of inflammatory cells infiltrated the dermis, and hair follicles decreased (Figure 1).

### The Differentiation Proportion of Treg and Th1 Cells in the Splenocytes of Sclerodermatous Chronic Graft-Versus-Host Disease Mice

**FIGURE 2:**
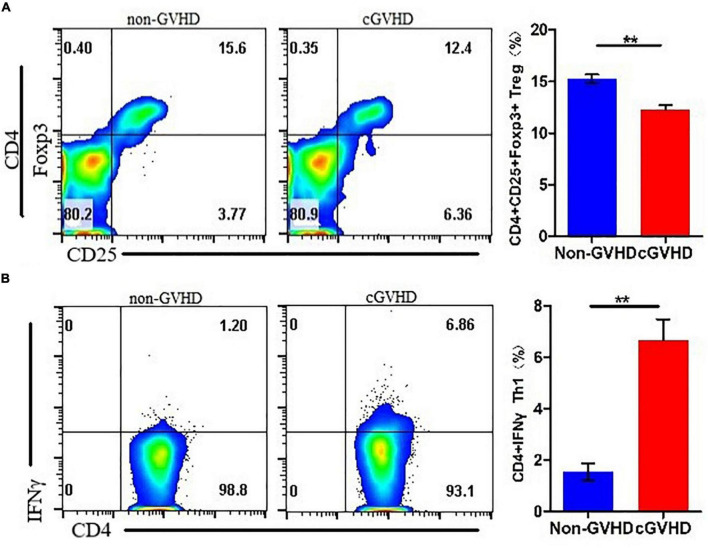
Decreased proportion of Treg cells and increased Th1 cells in splenocytes of sclerodermatous cGVHD mice. **(A)** The proportion of Treg cells in the spleen cells of cGVHD mice was significantly reduced (*n* = 3, **p < 0.01). **(B)** Increased Th1 cells in splenocytes of sclerodermatous cGVHD (*n* = 3, **p < 0.01).

To understand the differentiation of Treg and Th1 cells in splenocytes of sclerodermatous cGVHD mice, we detected the proportion of CD4 + CD 25^+^Foxp3^+^ Tregs (Figure 2A) and CD4^+^IFN-γ^+^ Th1 cells (Figure 2B) by flow cytometry and found a decreased proportion of Treg cells and increased Th1 cells in splenocytes of sclerodermatous cGVHD mice.

### Composition and Diversity of Gut Bacteria in Mice Model of Sclerodermatous Chronic Graft-Versus-Host Disease

**FIGURE 3:**
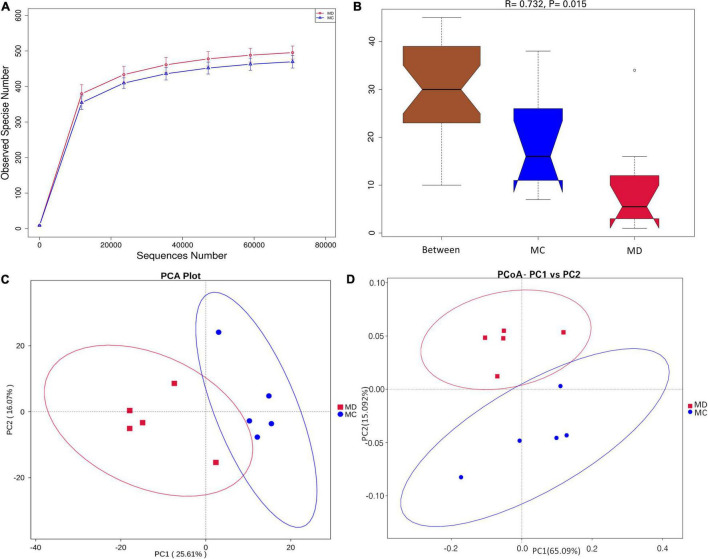
The composition and diversity of gut bacteria in mice model of sclerodermatous cGVHD. Fecal pellets were collected 35 days after transplantation [*n* = 10, 5 sclerodermatous cGVHD (MD group) and 5 non-GVHD mice (MC group)] and were subjected to 16S rDNA sequencing analysis. **(A)** Rarefaction curves of the species number in the sclerodermatous cGVHD (MD group) and non-GVHD mice (MC group). **(B)** ANOISM analysis of the beta diversity of the samples significantly separated the groups when *R* > 0 and *p* < 0.05. **(C,D)** Principal component analysis (PCA) and principal coordinates analysis (PCoA) plot (weighted_UniFrac distances) of beta diversity showed the difference in overall community structures between sclerodermatous cGVHD and non-GVHD mice.

To determine the composition and diversity of gut bacteria on the sclerodermatous cGVHD mice, we analyzed fecal pellets collected at a diseased time point (35 days) after transplantation. The gut bacteria communities in the mice model of sclerodermatous cGVHD changed compared with non-GVHD mice by 16S rDNA sequencing (Figure 3). The rarefaction analysis curves in the Scl-cGVHD and non-GVHD groups were smooth and approached to saturation (Figure 3A). It indicated that the sequencing data had high quality and adding more data would only produce a very small number of new species. Analysis of community structure between the groups showed a significant separation [*R* = 0.732, *p* = 0.015, non-parametric analysis (ANOSIM)] (Figure 3B), suggesting a clear difference in the gut bacteria composition in the Scl-cGVHD and non-GVHD mice. PCA and PCoA (weighted_UniFrac distances) of beta diversity showed a clear distinction of gut bacteria between the sclerodermatous cGVHD and non-GVHD mice (Figures 3C,D).

**FIGURE 4:**
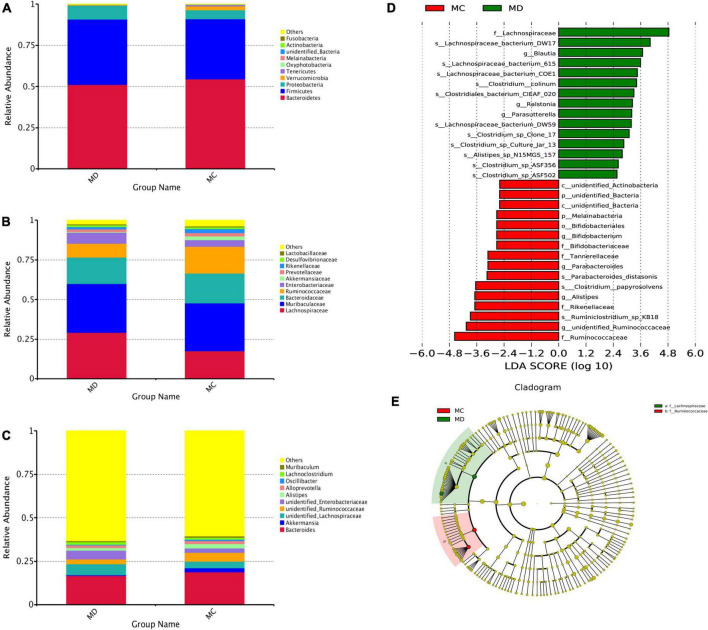
The specific bacterial taxa associated with sclerodermatous cGVHD. **(A–C)** The top 10 relative abundances of bacteria at phylum **(A)**, family **(B)**, and genus **(C)** levels in fecal samples from the mice with or without sclerodermatous cGVHD. **(D,E)** LEfSe analysis indicated that the listed microbiomes were significantly different (*p* < 0.05).

To determine whether the absence or presence of specific bacterial taxa was associated with sclerodermatous cGVHD, we compared the gut bacteria composition of the sclerodermatous cGVHD and non-GVHD mice (Figure 4). The top10 microbes at the phylum (Figure 4A), family (Figure 4B), and genus (Figure 4C) levels and the specific bacterial taxa by LEfSe analysis were shown in Figures 4D,E, indicating the variations in the composition of the gut bacteria. Analyses of microbes at the phylum level revealed the dominance of *Bacteroidetes*, *Firmicutes*, and *Proteobacteria* in both GVHD and non-GVHD mice. The relative abundance of the phylum *Bacteroidetes*, *Firmicutes*, and *Proteobacteria* was not different between the non-GVHD and sclerodermatous cGVHD mice. The Phylum *Melainabacteria* was lower in the sclerodermatous cGVHD mice. At the family level, the gut microbiota *Lachnospiraceae* was enriched in the fecal of sclerodermatous cGVHD mice compared with the non-GVHD mice, and the dominant specific species in *Lachnospiraceae*, such as *Lachnospiraceae_bacterium_DW17*, *Lachnospiraceae_bacterium_615*, *Lachnospiraceae_bacterium_C OE1*, and *Lachnospiraceae_bacterium_DW59* were increased in Scl-cGVHD mice. Whereas, the gut bacteria community in the sclerodermatous cGVHD mice was decreased in the family *Ruminococcaceae*, *Rikenellaceae, Tannerellaceae, and Bifidobacteriaceae.* At the genus level, the genus *unidentified_Ruminococcaceae*, *Alistipes*, *Parabacteriodes*, *and Bifidobacterium* were lower and *Blautia*, *Ralstonia*, and *Parasutterella* were higher in sclerodermatous cGVHD mice. Specifically, we also observed other gut bacteria species, such as *Ruminiclostridium_sp_KB18*, *Clostridium_papyrosolven*s, and *Parabacteroids_distasonis* were decreased in sclerodermatous cGVHD mice (Supplementary Material).

### Sclerodermatous Chronic Graft-Versus-Host Disease-Associated Gut Bacteria Inhibit Tregs and Promote Th1 Cells Differentiation *in vitro*

**FIGURE 5:**
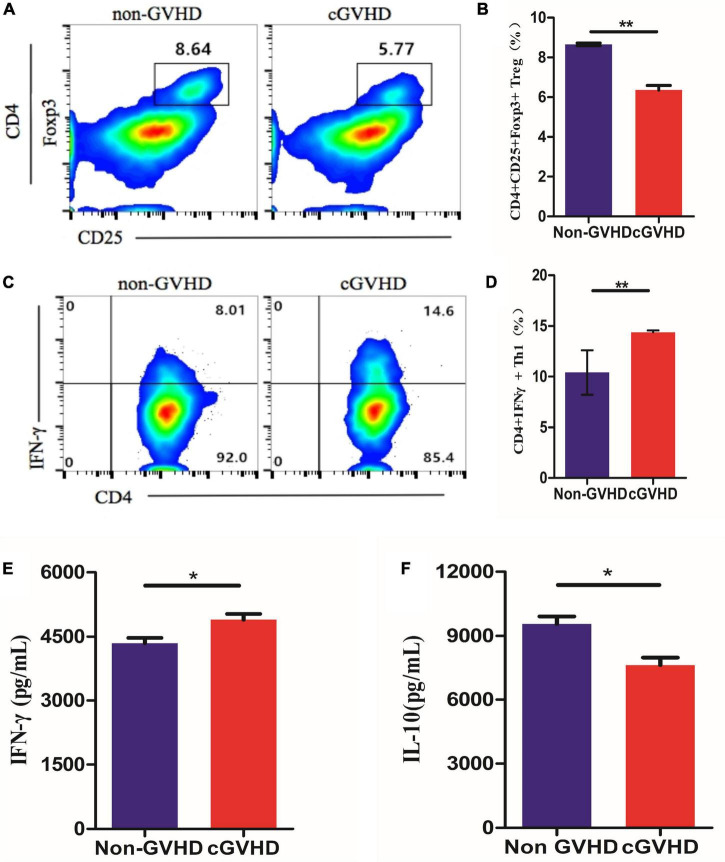
Sclerodermatous cGVHD-associated gut bacteria inhibit Tregs and promote Th1 lymphocytes Differentiation *in vitro*. **(A,B)** Representative flow cytometry plots and quantification of CD25^+^Foxp3^+^ Tregs within CD4^+^ T cells differentiated in response to bacterial extracts from sclerodermatous cGVHD and non-GVHD mice (*n* = 3, **p < 0.01). **(C,D)** The percentage of interferon- γ (IFN-γ^+^) in CD4^+^ T cells significantly increased by stimulating bacterial extracts from sclerodermatous cGVHD compared with the non-GVHD group (*n* = 3, **p < 0.01). **(E,F)** The concentration of anti-inflammatory interleukin-10 (IL-10) and IFN-γ was detected by the CBA kit. The secretion of IL-10 decreased but IFN-γ increased markedly in the supernatant stimulated by bacterial extract from sclerodermatous cGVHD mice (*n* = 3, *p < 0.01).

To validate the hypothesis that gut bacteria taxa altered in sclerodermatous cGVHD mice play functional roles in regulating immune responses. To validate the hypothesis, we established an *in vitro* model system by exposing splenocytes from healthy female BALB/cJ mice to a suspension of bacterial extracts that were heat-killed and sonicated gut microbes of Scl-cGVHD and non-GVHD mice and used the flow cytometry to test T-cells differentiation. We found that the bacterial extracts from the sclerodermatous cGVHD mice reduced the proportions of CD 25^+^Foxp3^+^ Tregs within CD4^+^ T cells (Figures 5A,B) and promoted the differentiation of Th1 (CD4^+^IFN-γ^+^) cells (Figures 5C,D). These results indicated that gut bacteria from sclerodermatous cGVHD mice restrains immunoregulatory T-cell development and potentially exacerbates inflammation. Furthermore, we examined the proinflammatory (IFN-γ) and anti-inflammatory (IL-10) cytokines in three sclerodermatous cGVHD and three non-GVHD plates after incubating SPC for 5 days with bacteria extracts. The bacterial extracts from sclerodermatous cGVHD mice tended to induce an increased concentration of IFN-γ production from T cells but decreased anti-inflammatory IL-10 levels in comparison with non-GVHD mice (Figures 5E,F).

## Discussion

Numerous studies on microbes have demonstrated that the gut bacteria community was involved in the etiopathogenesis of several inflammatory autoimmune diseases (Forbes et al., 2018; van der Meulen et al., 2019). However, the diversity and composition of the gut bacteria on sclerodermatous cGVHD were not reported. In our study, we analyzed the gut bacteria from sclerodermatous cGVHD mice by using 16S rDNA sequencing. In sclerodermatous cGVHD mice, significant difference in the gut bacteria community was observed versus non-GVHD mice. The microbiota from sclerodermatous cGVHD mice tended to be less diverse. Fewer relative abundance of *Ruminococcaceae* and more *Lachnospiraceae* were found in the sclerodermatous cGVHD mice. The studies of *Ruminococcaceae* were consistent with other studies (Han et al., 2018), which reported that *Ruminococcaceae* was negatively corrected with severe aGVHD by regulating the differentiation ratio of Treg cells in human aGVHD. A significantly lower relative abundance of the *Lachnospiraceae* was shown in gut aGVHD. However, we observed a higher relative abundance of the *Lachnospiraceae* in Scl-cGVHD mice. This is possibly due to differences in the study, such as (1) there may be similarities in the gut bacteria of patients with sclerodermatous cGVHD versus non-GVHD; (2) our mouse model is mainly characterized by scleroderma, which is different from gut aGVHD and may be more likely to the SLE, which was also characterized with autoimmune antibodies and it was reported that *Lachnospiraceae* was increased in patients with SLE and mice.

A study by Im A showed that Th1 was the predominant cytotoxic effector on the skin in cGVHD (Im et al., 2016), and we found that the percentage of Treg cells was decreased in the sclerodermatous cGVHD mice in our previous study (Wang et al., 2017). In addition, we found a decreased percentage of Treg cells and an increased percentage of Th1 cells in splenocytes of Scl-cGVHD mice. While it was always consistent with the studies previously reported. Interactions between the differentiation of CD4^+^ T cells and commensal gut bacteria were not well understood. Recently, it was shown that the *Bacteroidales* regulated the differentiation of Treg cells and *A. calcoaceticus* and *A. muciniphila* induced the differentiation of Th1 cells. *Rumminococaceae* was found to be positively associated with the differentiation of Treg cells and *Enterobacteriaceae* was negatively associated with it. In this study, we prepared the bacterial extracts of the microbiota from sclerodermatous cGVHD mice to validate the regulatory function *in vitro*. We first examined the ratio of Th1 cells and Treg cells and proinflammatory IFN-γ and immunoregulatory IL-10 cytokines in three sclerodermatous cGVHD and three non-GVHD mice after incubating spleen cells with bacterial extracts. We found that the bacterial extracts from sclerodermatous cGVHD mice demonstrated the regulatory effect on the differentiation of Th1 and Treg cells by inducing the secretion of immunosuppressive cytokine IFN-γ and inhibiting immunoregulatory IL-10. It will be important in further studies that we try to enroll a population of sclerodermatous cGVHD patients and analyze the gut microbiotas. *In vitro*, we will prepare the bacterial extracts of gut bacteria from patients with sclerodermatous cGVHD and non-GVHD and culture the specific microbiome to verify the function of the differentiation of CD4^+^ T cells. Furthermore, in the sclerodermatous cGVHD mice model, we will evaluate the curative effect on fecal microbiota transplantation by gavaging the bacteria extracts from non-GVHD mice to the sclerodermatous cGVHD mice. In this way, the influence of gut bacteria on patients with sclerodermatous cGVHD patients and mice models may be achieved, enabling us to understand whether fecal microbiota transplantation may be used as a new target treatment for sclerodermatous cGVHD.

## Conclusion

In conclusion, our results demonstrate an altered gut bacteria community in our established sclerodermatous cGVHD mice. Further, the abnormal gut bacteria extracts from the sclerodermatous cGVHD mice would inhibit the differentiation ratio of Tregs and promote the differentiation of the Th1 cells by regulating the secretion of proinflammatory cytokine IFN-γand the anti-inflammatory cytokine IL-10. Our data provide evidence that dysbiosis of the gut bacteria community may be a potential risk factor for the development of cGVHD. Regulation of the gut bacteria community would be a novel therapy for cGVHD treatment.

## Data Availability Statement

The datasets presented in this study can be found in online repositories. The names of the repository/repositories and accession number(s) can be found below: Dryad, https://doi.org/10.5061/dryad.zs7h44jd1.

## Ethics Statement

The animal study was reviewed and approved by the Animal Experimental Ethics Committee of Guangdong Provincial People’s Hospital.

## Author Contributions

YW, PL, XD, and JW conceived and designed the experiments and wrote the manuscript. YW, LH, TH, XC, SG, and XH performed the experiments. YW and PL analyzed the data. All authors contributed to the article and approved the submitted version.

## Conflict of Interest

The authors declare that the research was conducted in the absence of any commercial or financial relationships that could be construed as a potential conflict of interest.

## Publisher’s Note

All claims expressed in this article are solely those of the authors and do not necessarily represent those of their affiliated organizations, or those of the publisher, the editors and the reviewers. Any product that may be evaluated in this article, or claim that may be made by its manufacturer, is not guaranteed or endorsed by the publisher.
