# Supplementary material for: The Gut Bacteria Dysbiosis Contributes to Chronic Graft-Versus-Host Disease Associated With a Treg/Th1 Ratio Imbalance
Source: Front Microbiol. 2022 Sep 8;13:813576. doi: 10.3389/fmicb.2022.813576 (PMC9493085; doi:10.3389/fmicb.2022.813576)
Supplement: Supplementary file 1 [file Data_Sheet_1.zip › P101SC18090073-01-B1-3-4_result/02.OTUanalysis/genus_evolutionary_tree_group/genus_group_100.tree.pdf]

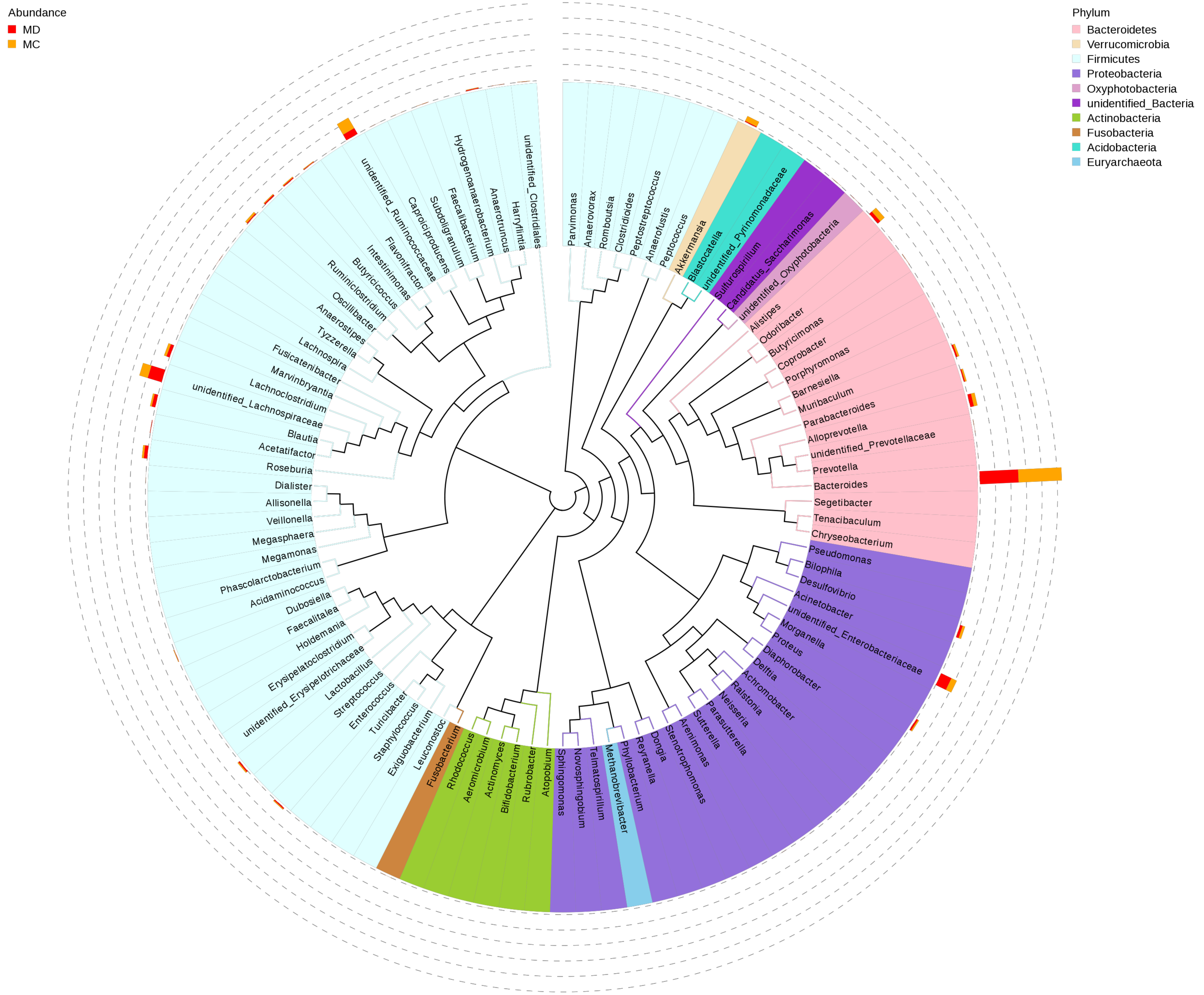

Abundance
MD
MC
Phylum
Bacteroidetes
Verrucomicrobia
Firmicutes
Proteobacteria
Oxyphotobacteria
unidentified_Bacteria
Actinobacteria
Fusobacteria
Acidobacteria
Euryarchaeota
Parvimonas
Anaerovorax
Romboutsia
Clostridioides
Peptostreptococcus
Anaerofustis
Peptococcus
Akkermansia
Blastocatella
unidentified_Pyrinomonadaceae
Sulfurospirillum
Candidatus_Saccharimonas
unidentified_Oxyphotobacteria
Alistipes
Odoribacter
Butyricimonas
Coprobacter
Porphyromonas
Barnesiella
Muribaculum
Parabacteroides
Alloprevotella
unidentified_Prevotellaceae
Prevotella
Bacteroides
Segetibacter
Tenacibaculum
Chryseobacterium
Pseudomonas
Bilophila
Desulfovibrio
Acinetobacter
unidentified_Enterobacteriaceae
Morganella
Proteus
Diaphorobacter
Delftia
Achromobacter
Ralstonia
Neisseria
Parasutterella
Sutterella
Arenimonas
Stenotrophomonas
Dongia
Reyranella
Phyllobacterium
Methanobrevibacter
Telmatospirillum
Novosphingobium
Sphingomonas
Atopobium
Rubrobacter
Bifidobacterium
Actinomyces
Aeromicrobium
Rhodococcus
Fusobacterium
Leuconostoc
Exiguobacterium
Staphylococcus
Turicibacter
Enterococcus
Streptococcus
Lactobacillus
unidentified_Erysipelotrichaceae
Erysipelatoclostridium
Holdemania
Faecalitalea
Dubosiella
Acidaminococcus
Phascolarctobacterium
Megamonas
Megasphaera
Veillonella
Allisonella
Dialister
Roseburia
Acetatifactor
Blautia
unidentified_Lachnospiraceae
Lachnoclostridium
Marvinbryantia
Fusicatenibacter
Lachnospira
Tyzzerella
Anaerostipes
Oscillibacter
Ruminiclostridium
Butyricicoccus
Intestinimonas
Flavonifractor
unidentified_Ruminococcaceae
Caproiciproducens
Subdoligranulum
Faecalibacterium
Hydrogenoanaerobacterium
Anaerotruncus
Harryflintia
unidentified_Clostridiales
