# Supplementary material for: The Gut Bacteria Dysbiosis Contributes to Chronic Graft-Versus-Host Disease Associated With a Treg/Th1 Ratio Imbalance
Source: Front Microbiol. 2022 Sep 8;13:813576. doi: 10.3389/fmicb.2022.813576 (PMC9493085; doi:10.3389/fmicb.2022.813576)
Supplement: Supplementary file 1 [file Data_Sheet_1.zip › P101SC18090073-01-B1-3-4_result/03.AlphaDiversity/Alpha_div/Alpha_div.pdf]

```
|          |-- *.Tukey.txt
            --diff             【组间差值】
            --lwr              【置信区间上限】
            --upr              【置信区间下限】
            --p adj            【P 值】
|          |--*.wilcox.txt
            --Difference       【均值差】
            --pvalue           【P 值】
            --sig.             【是否显著,若 p 值<0.05,标*,p 值<0.01 标 **,p 值<0.001 标 ***】
            --LCL              【置信区间下限】
            --UCL              【置信区间上限】
```
