# Supplementary figures and images for: The Gut Bacteria Dysbiosis Contributes to Chronic Graft-Versus-Host Disease Associated With a Treg/Th1 Ratio Imbalance (part 1 of 2)
Source: Front Microbiol. 2022 Sep 8;13:813576. doi: 10.3389/fmicb.2022.813576 (PMC9493085; doi:10.3389/fmicb.2022.813576)

## Abundance

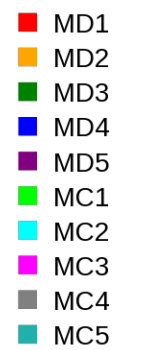

## Phylum

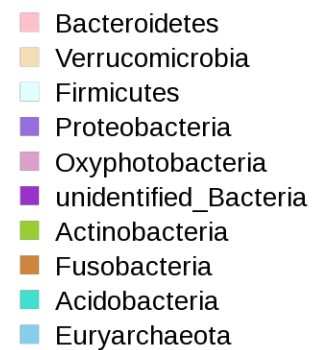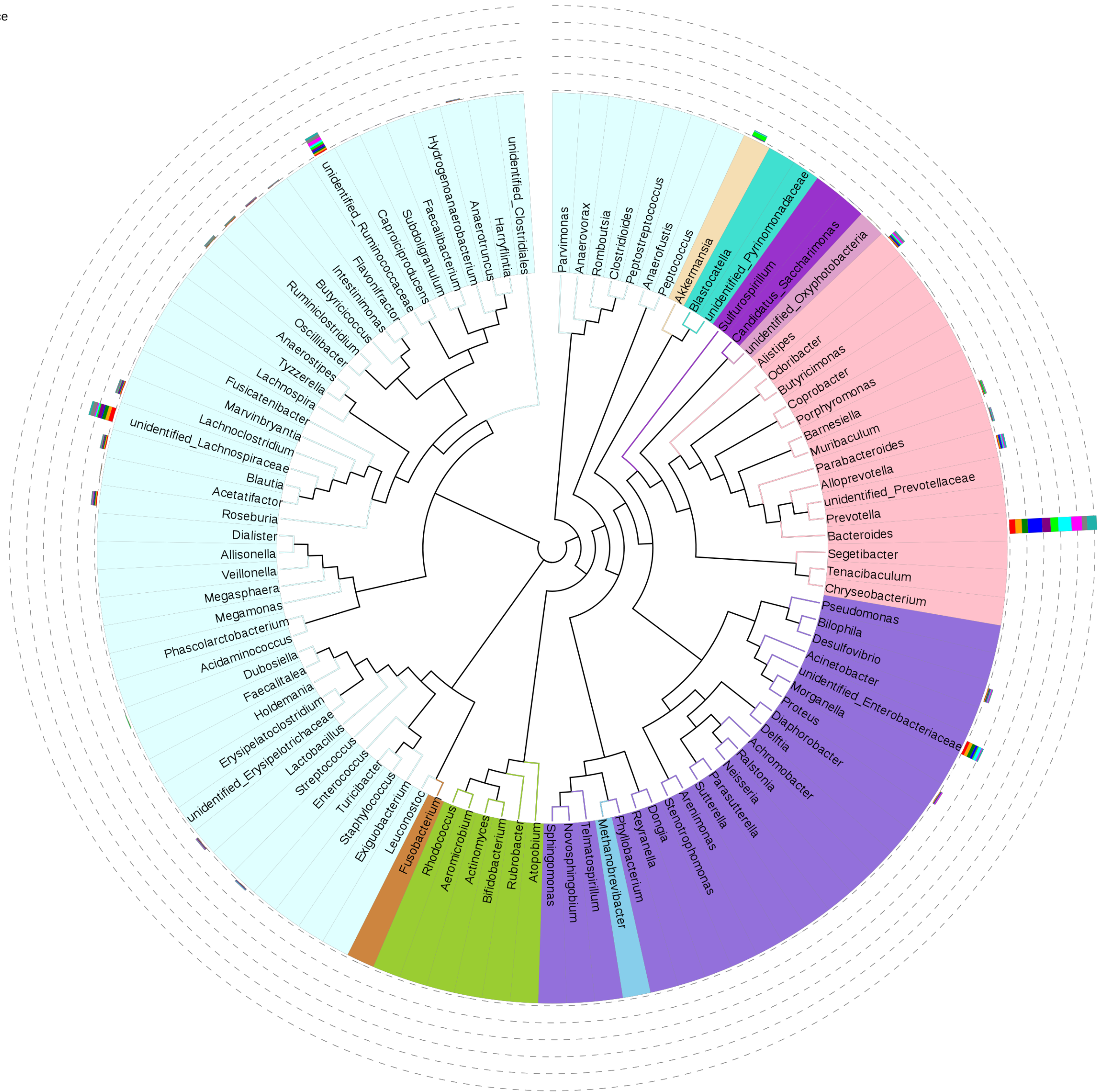

Supplement: Supplementary file 1 [file Data_Sheet_1.zip › P101SC18090073-01-B1-3-4_result/02.OTUanalysis/genus_evolutionary_tree/genus_100.tree.pdf]

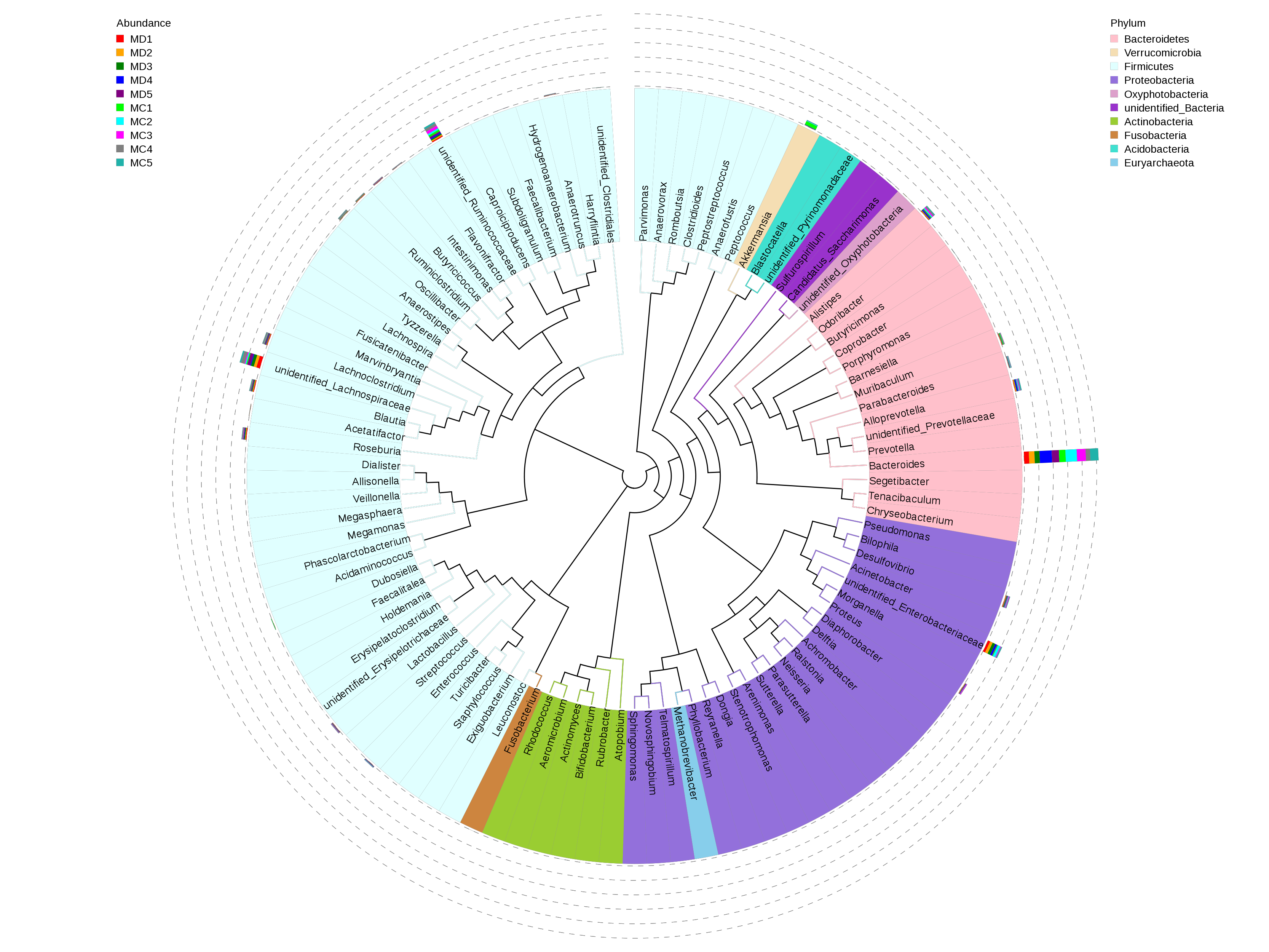

Supplement: Supplementary file 1 [file Data_Sheet_1.zip › P101SC18090073-01-B1-3-4_result/02.OTUanalysis/genus_evolutionary_tree/genus_100.tree.png]

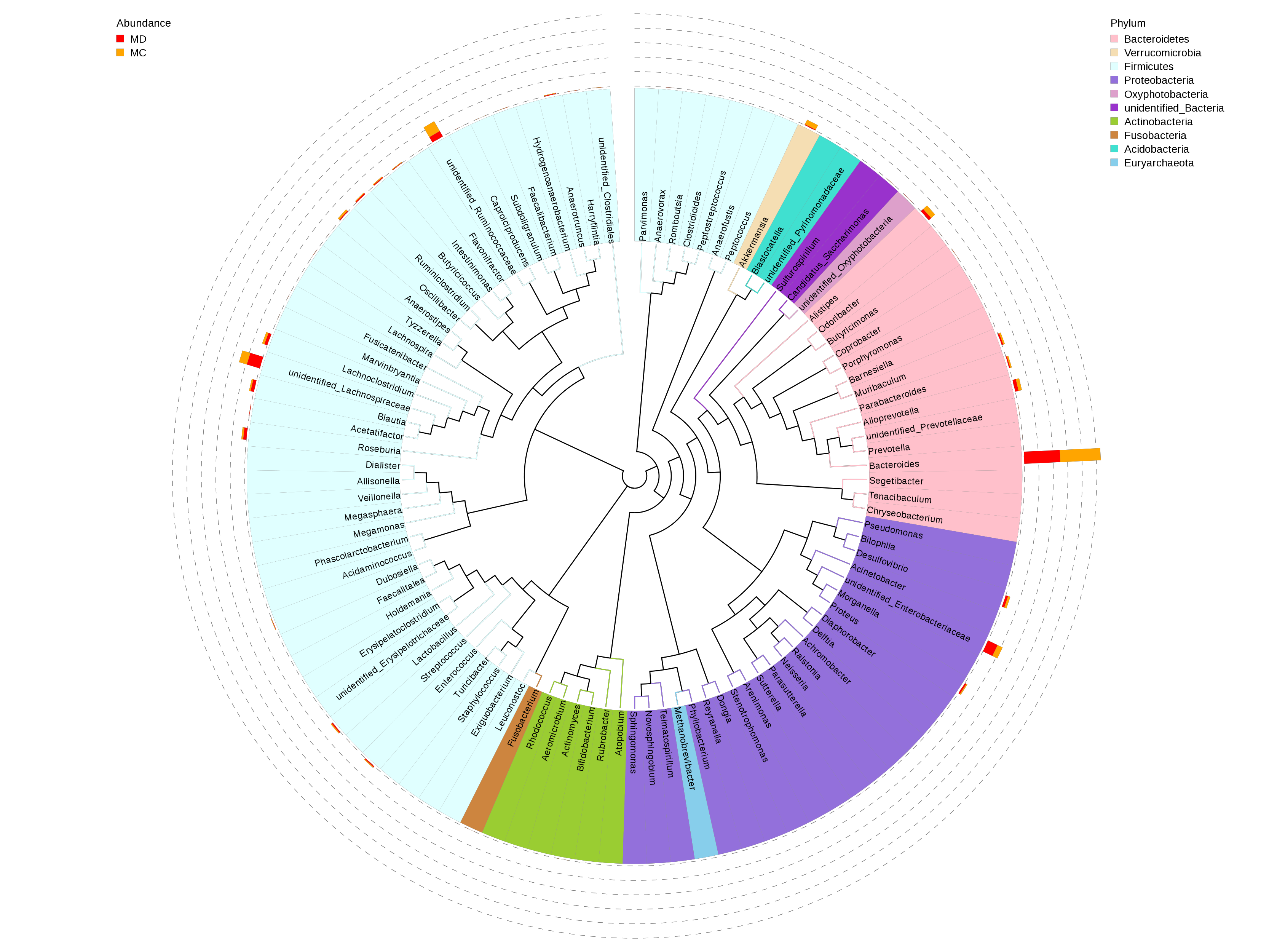

Supplement: Supplementary file 1 [file Data_Sheet_1.zip › P101SC18090073-01-B1-3-4_result/02.OTUanalysis/genus_evolutionary_tree_group/genus_group_100.tree.png]

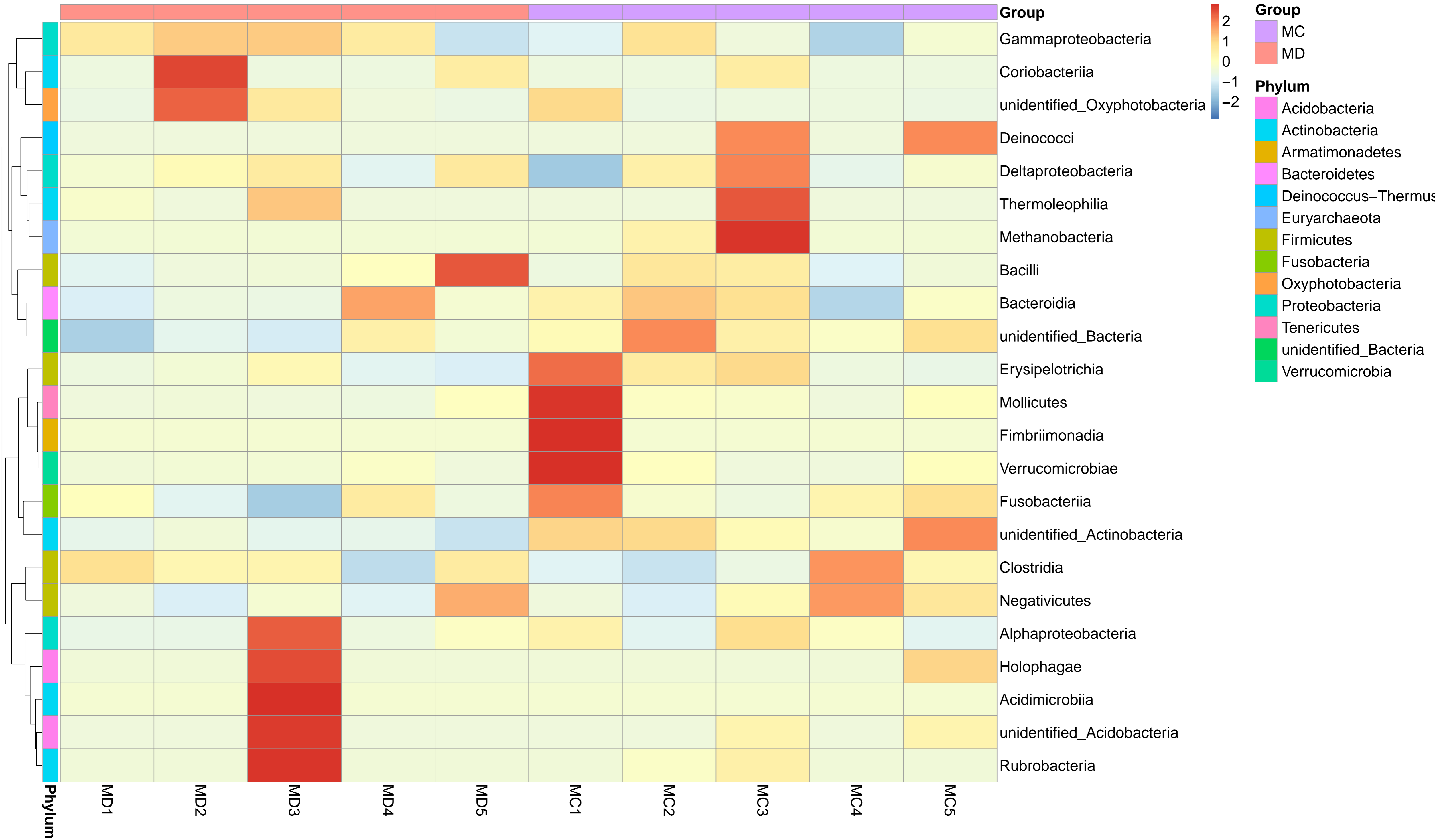

Supplement: Supplementary file 1 [file Data_Sheet_1.zip › P101SC18090073-01-B1-3-4_result/02.OTUanalysis/taxa_heatmap/cluster/cluster.c.pdf]

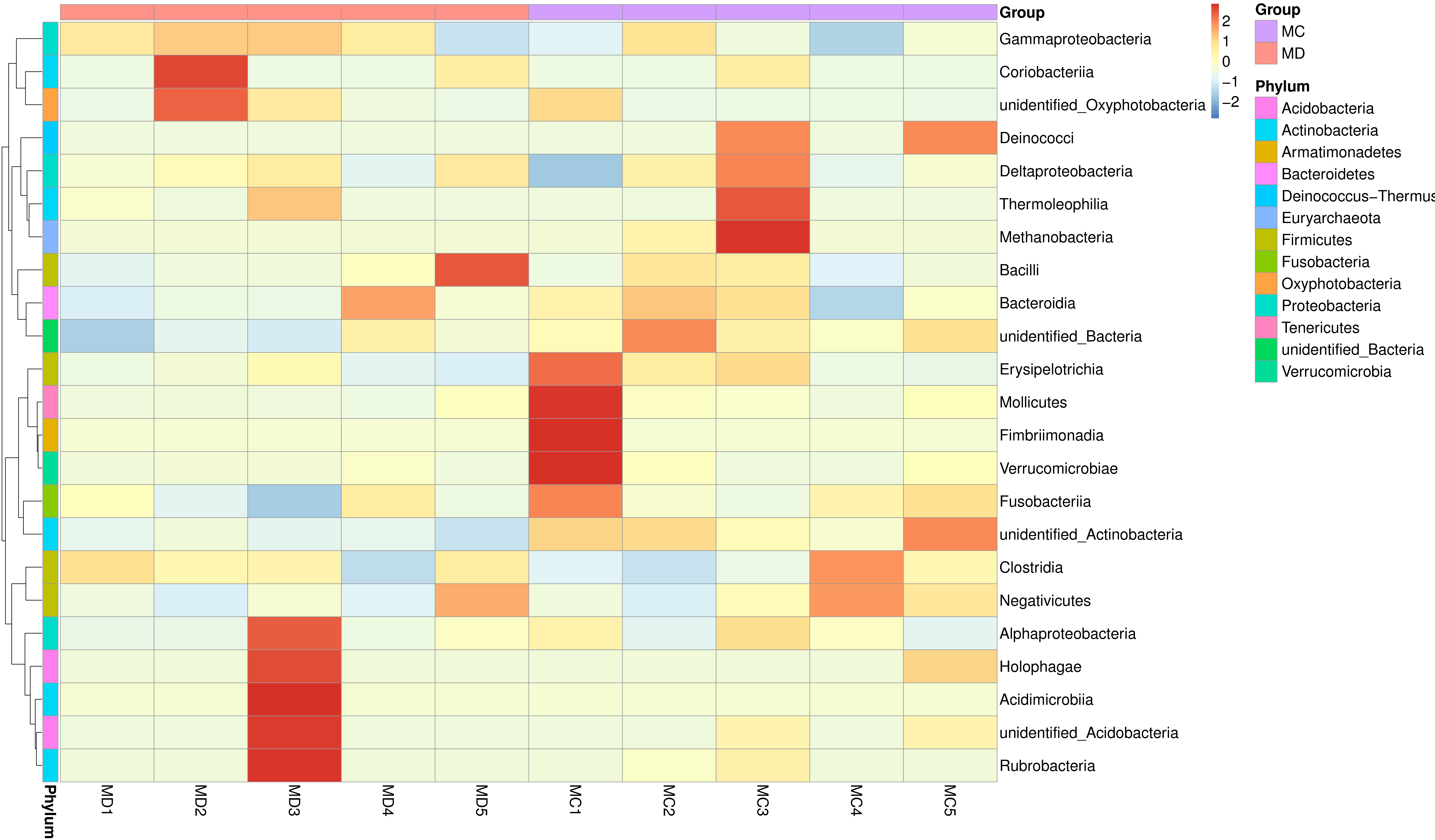

Supplement: Supplementary file 1 [file Data_Sheet_1.zip › P101SC18090073-01-B1-3-4_result/02.OTUanalysis/taxa_heatmap/cluster/cluster.c.png]

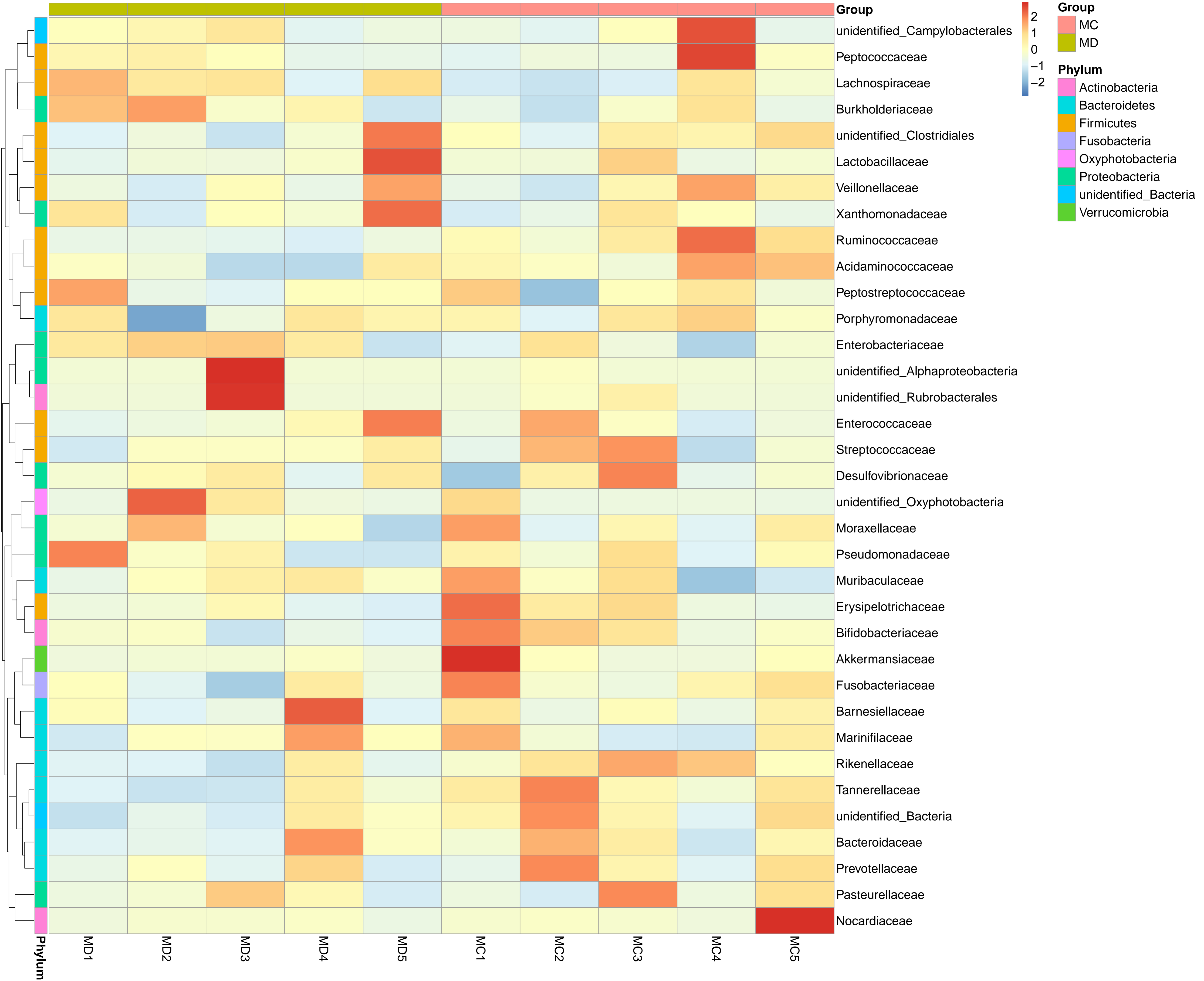

Supplement: Supplementary file 1 [file Data_Sheet_1.zip › P101SC18090073-01-B1-3-4_result/02.OTUanalysis/taxa_heatmap/cluster/cluster.f.pdf]

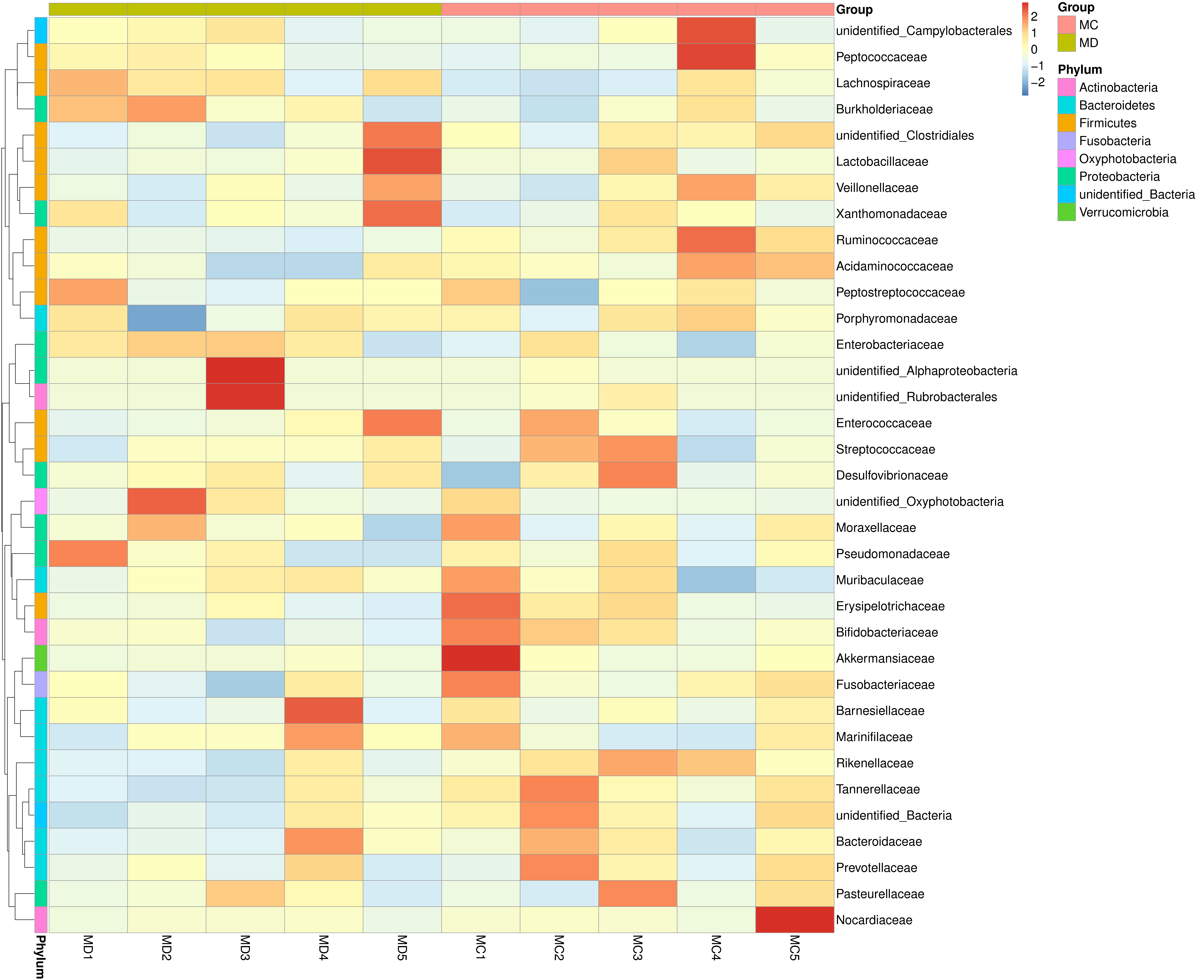

Supplement: Supplementary file 1 [file Data_Sheet_1.zip › P101SC18090073-01-B1-3-4_result/02.OTUanalysis/taxa_heatmap/cluster/cluster.f.png]

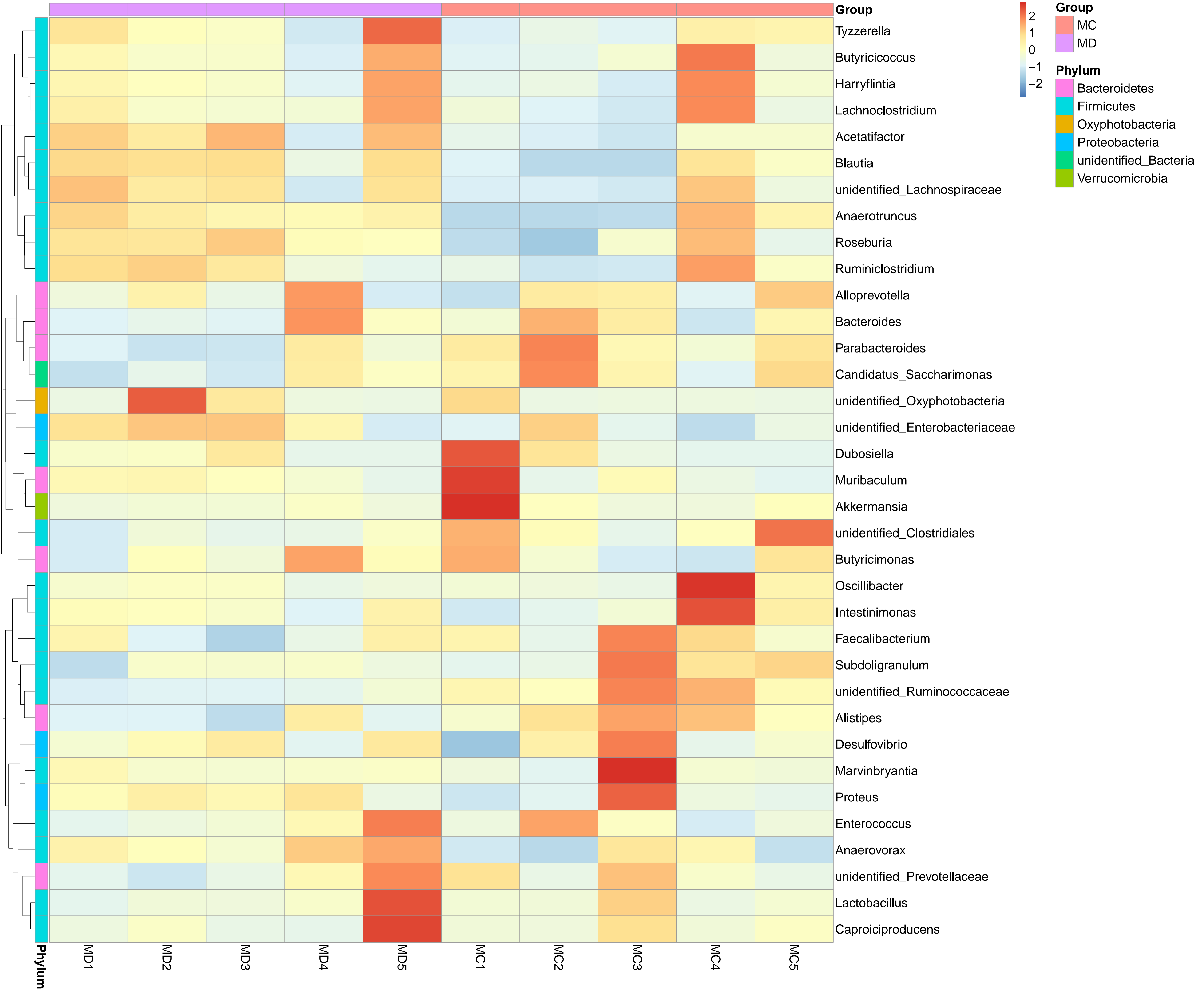

Supplement: Supplementary file 1 [file Data_Sheet_1.zip › P101SC18090073-01-B1-3-4_result/02.OTUanalysis/taxa_heatmap/cluster/cluster.g.pdf]

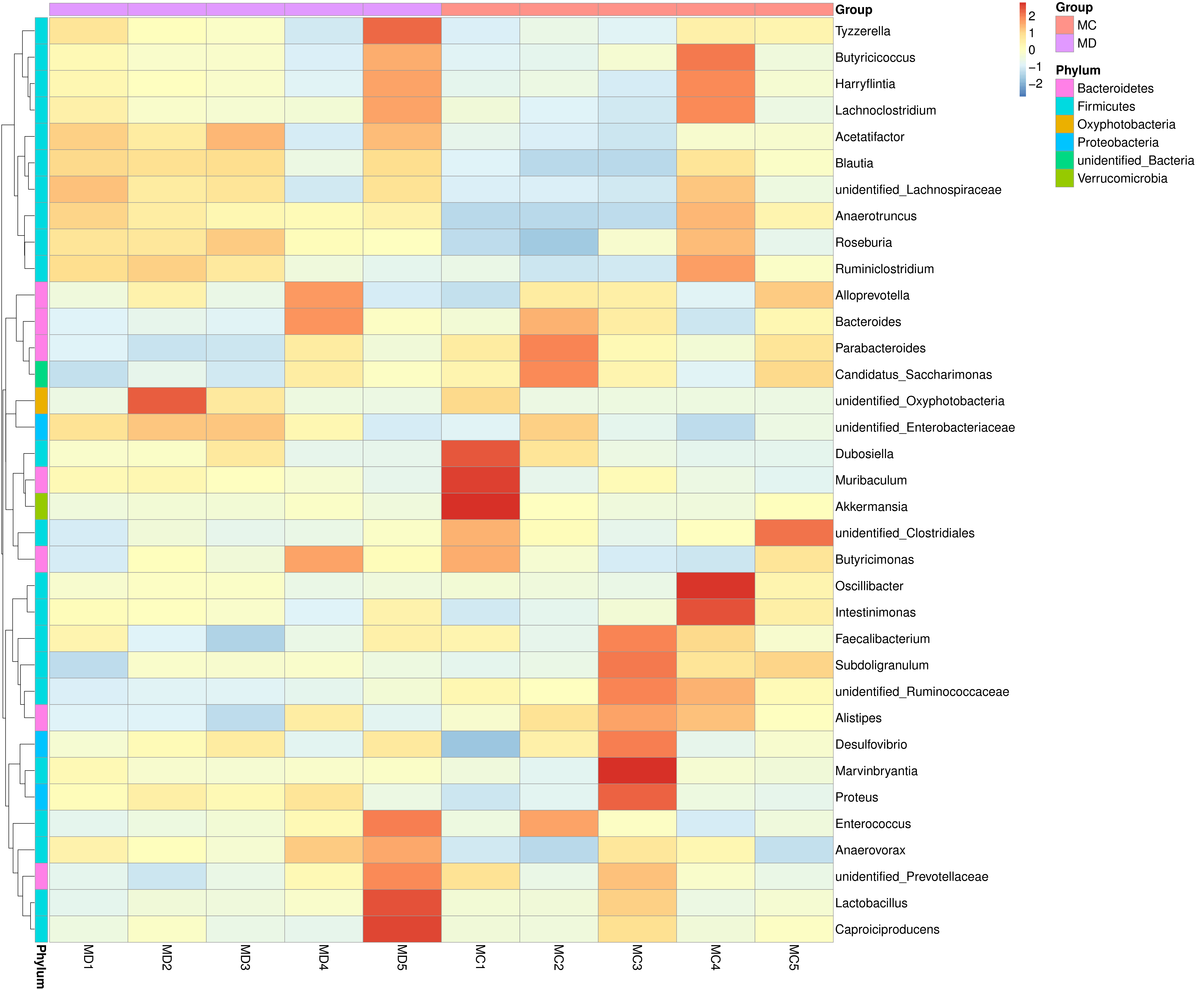

Supplement: Supplementary file 1 [file Data_Sheet_1.zip › P101SC18090073-01-B1-3-4_result/02.OTUanalysis/taxa_heatmap/cluster/cluster.g.png]

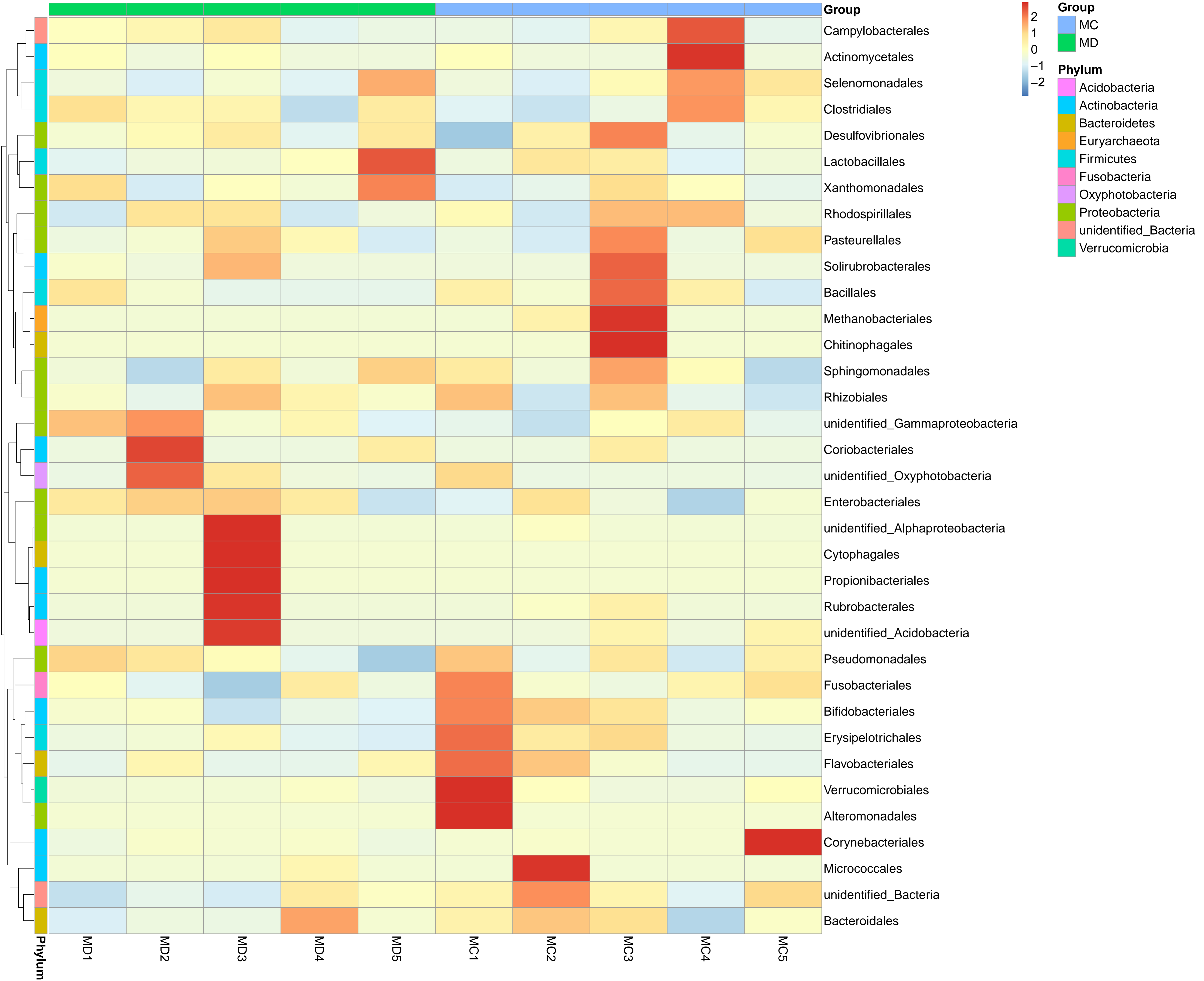

Supplement: Supplementary file 1 [file Data_Sheet_1.zip › P101SC18090073-01-B1-3-4_result/02.OTUanalysis/taxa_heatmap/cluster/cluster.o.pdf]

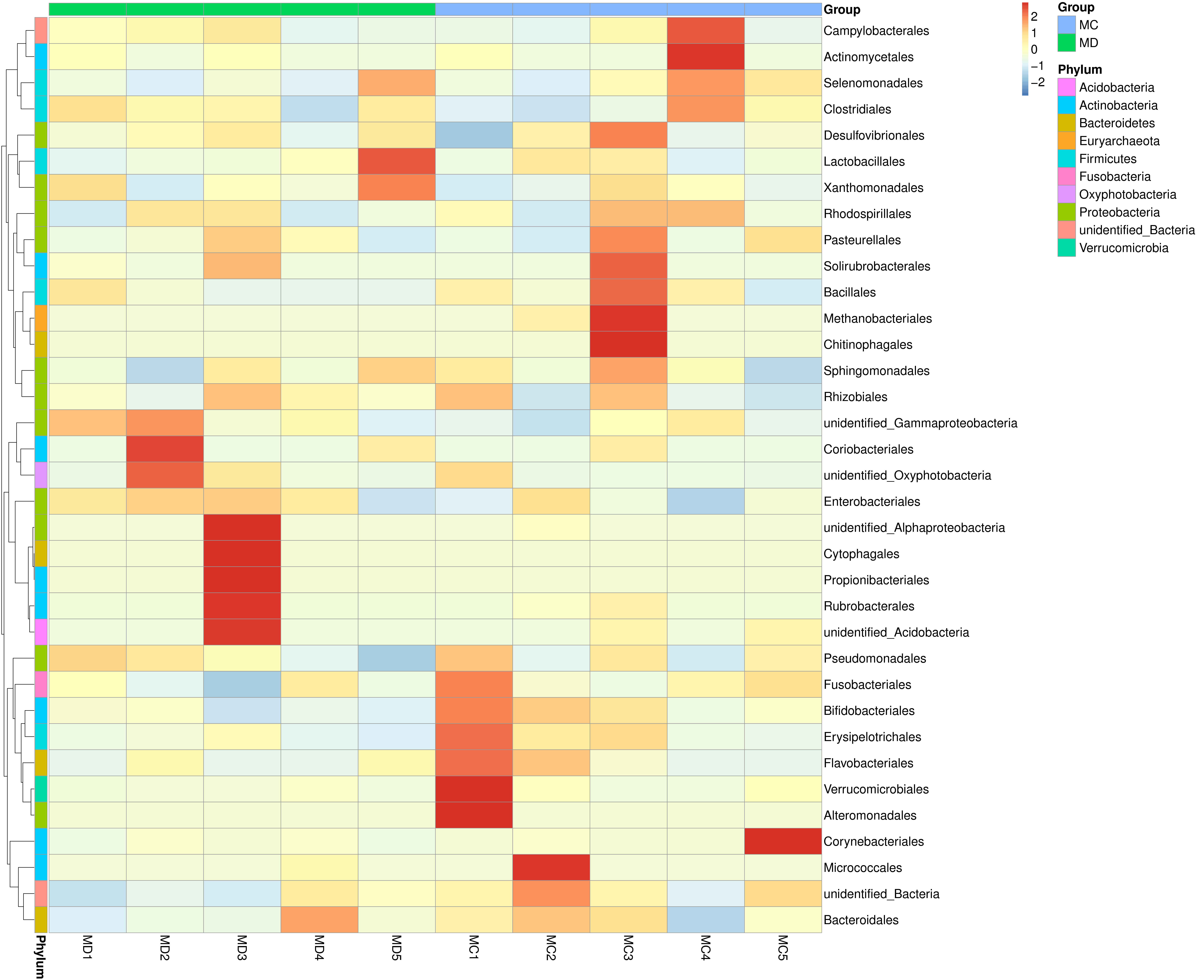

Supplement: Supplementary file 1 [file Data_Sheet_1.zip › P101SC18090073-01-B1-3-4_result/02.OTUanalysis/taxa_heatmap/cluster/cluster.o.png]

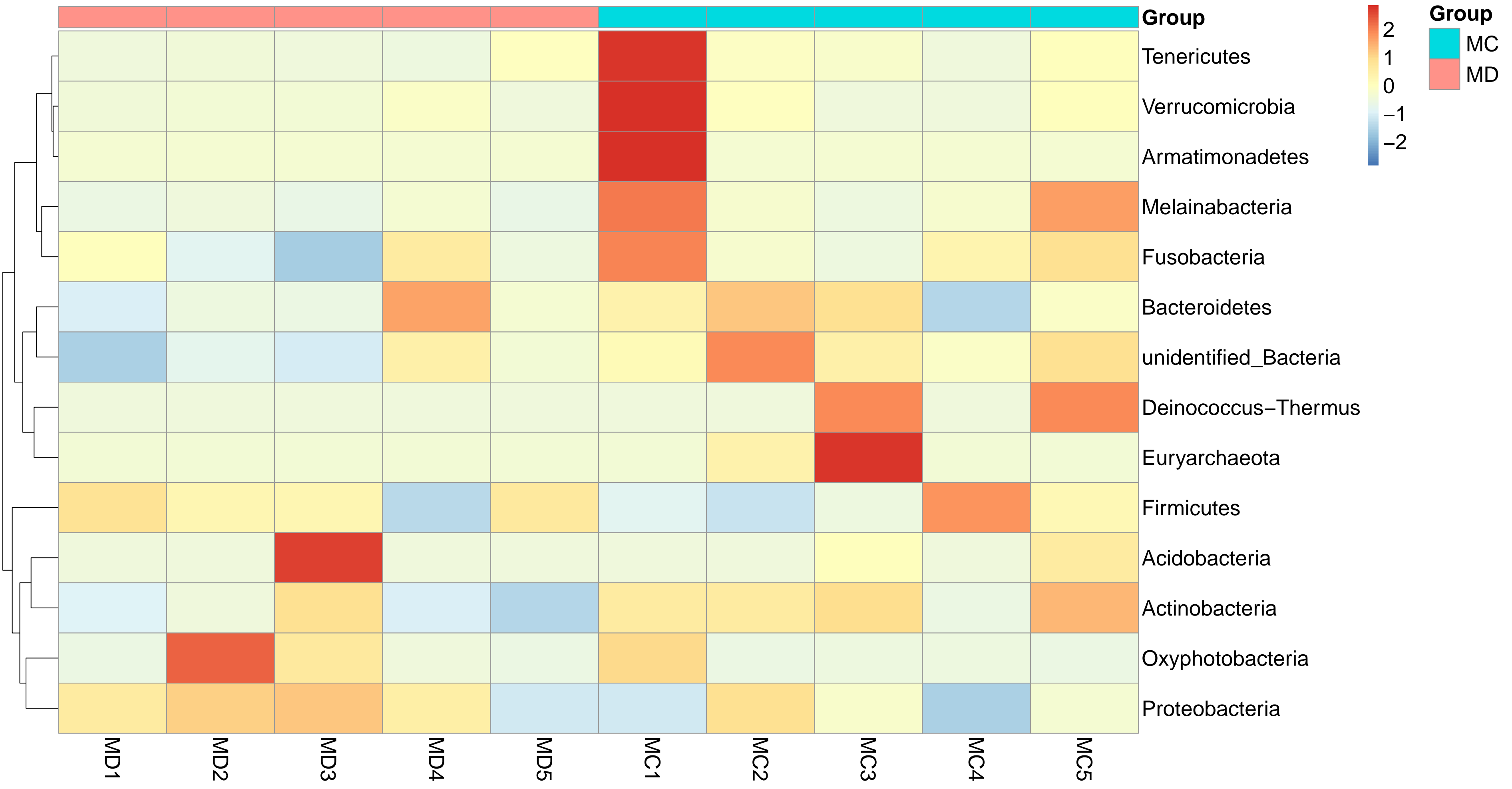

Supplement: Supplementary file 1 [file Data_Sheet_1.zip › P101SC18090073-01-B1-3-4_result/02.OTUanalysis/taxa_heatmap/cluster/cluster.p.pdf]

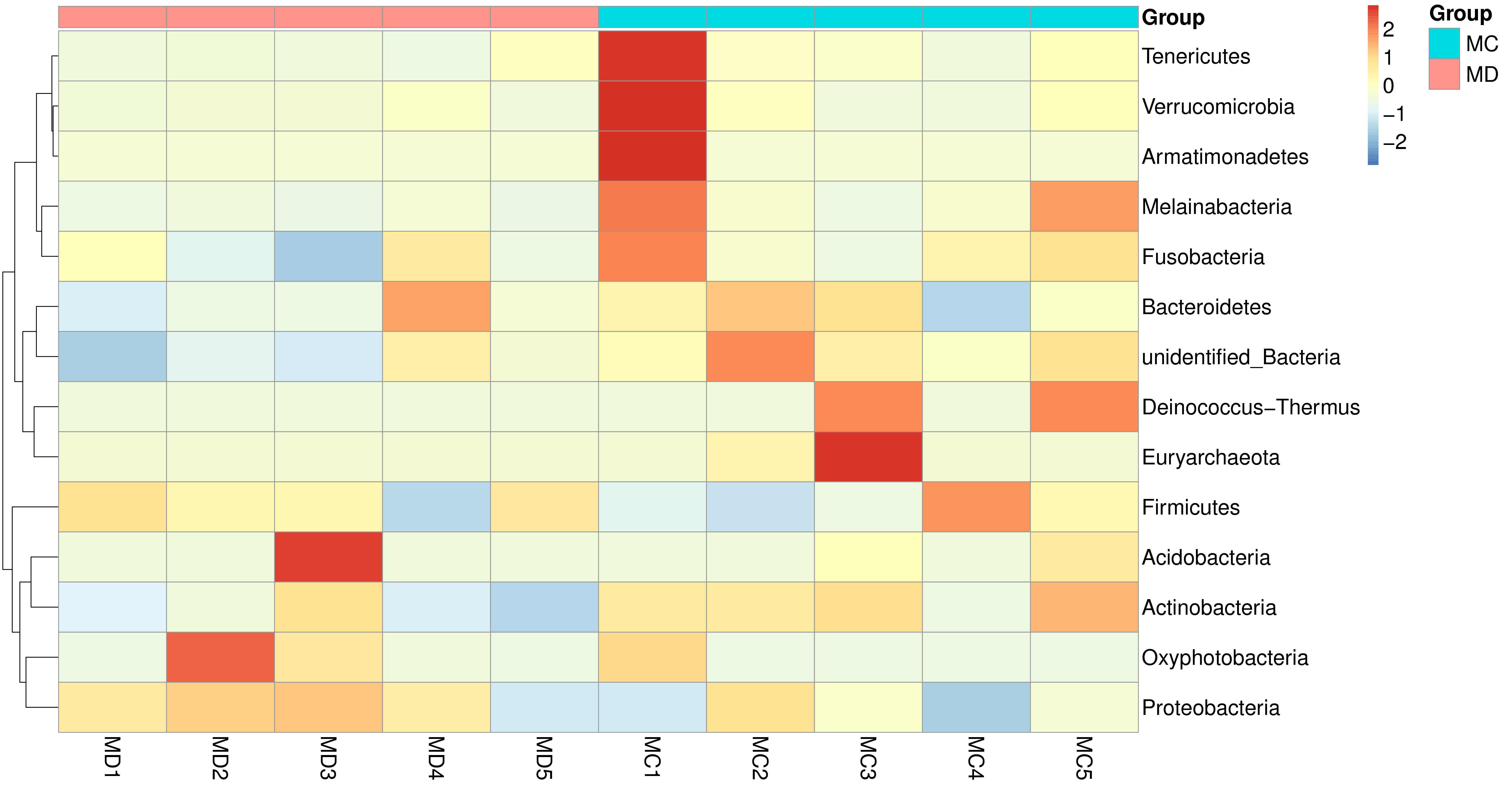

Supplement: Supplementary file 1 [file Data_Sheet_1.zip › P101SC18090073-01-B1-3-4_result/02.OTUanalysis/taxa_heatmap/cluster/cluster.p.png]

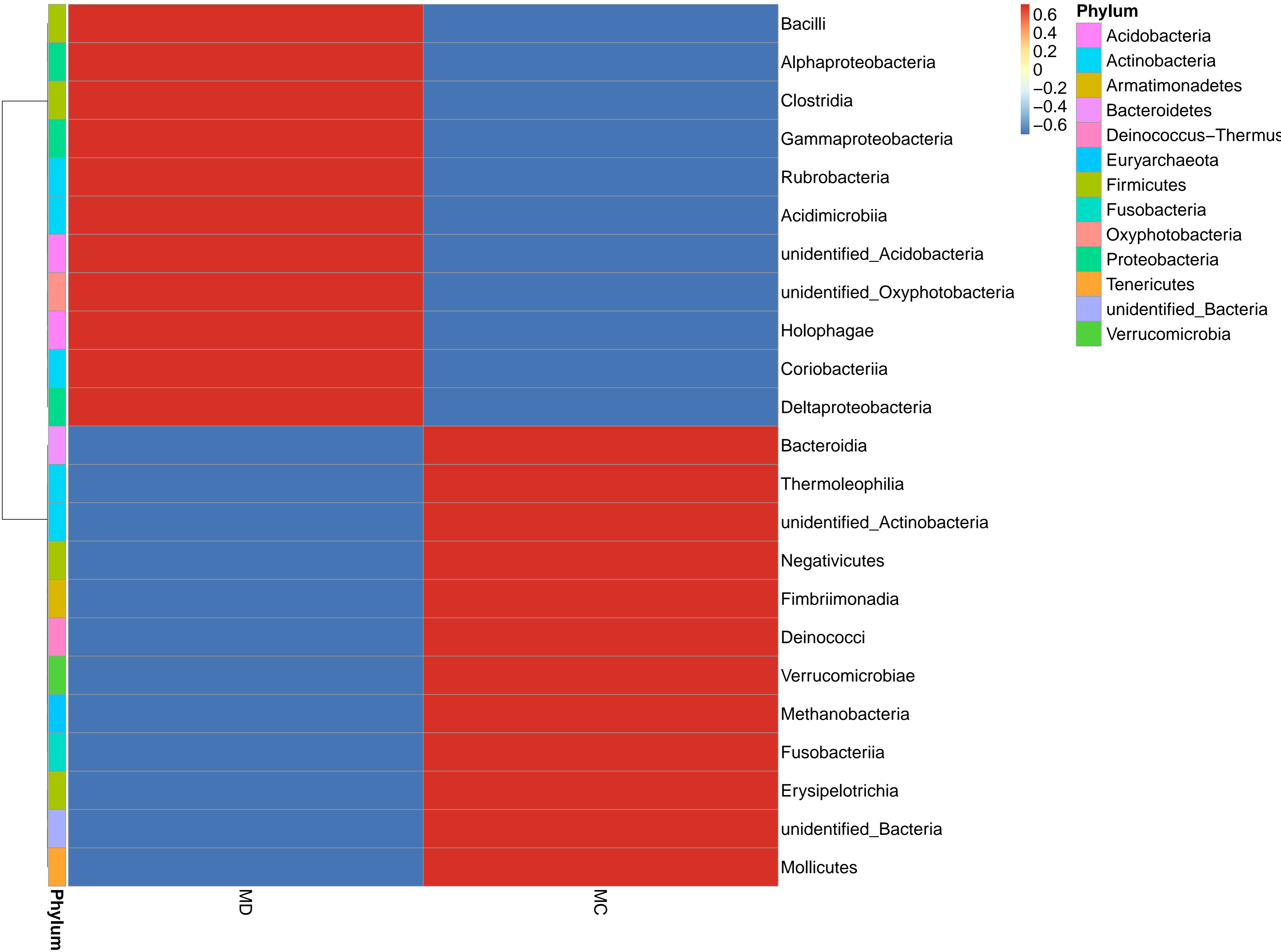

Supplement: Supplementary file 1 [file Data_Sheet_1.zip › P101SC18090073-01-B1-3-4_result/02.OTUanalysis/taxa_heatmap/cluster_group/cluster.c.pdf]

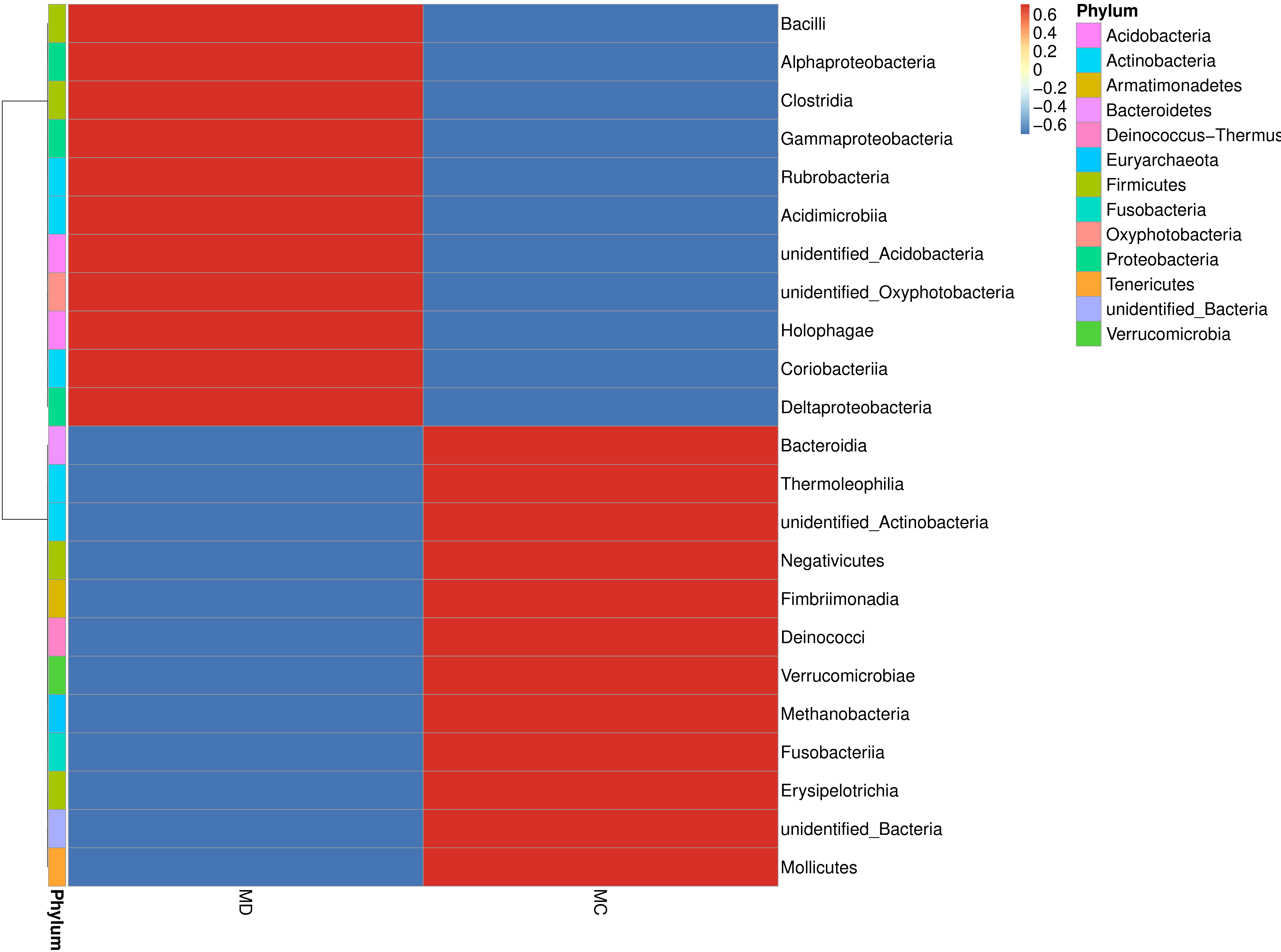

Supplement: Supplementary file 1 [file Data_Sheet_1.zip › P101SC18090073-01-B1-3-4_result/02.OTUanalysis/taxa_heatmap/cluster_group/cluster.c.png]

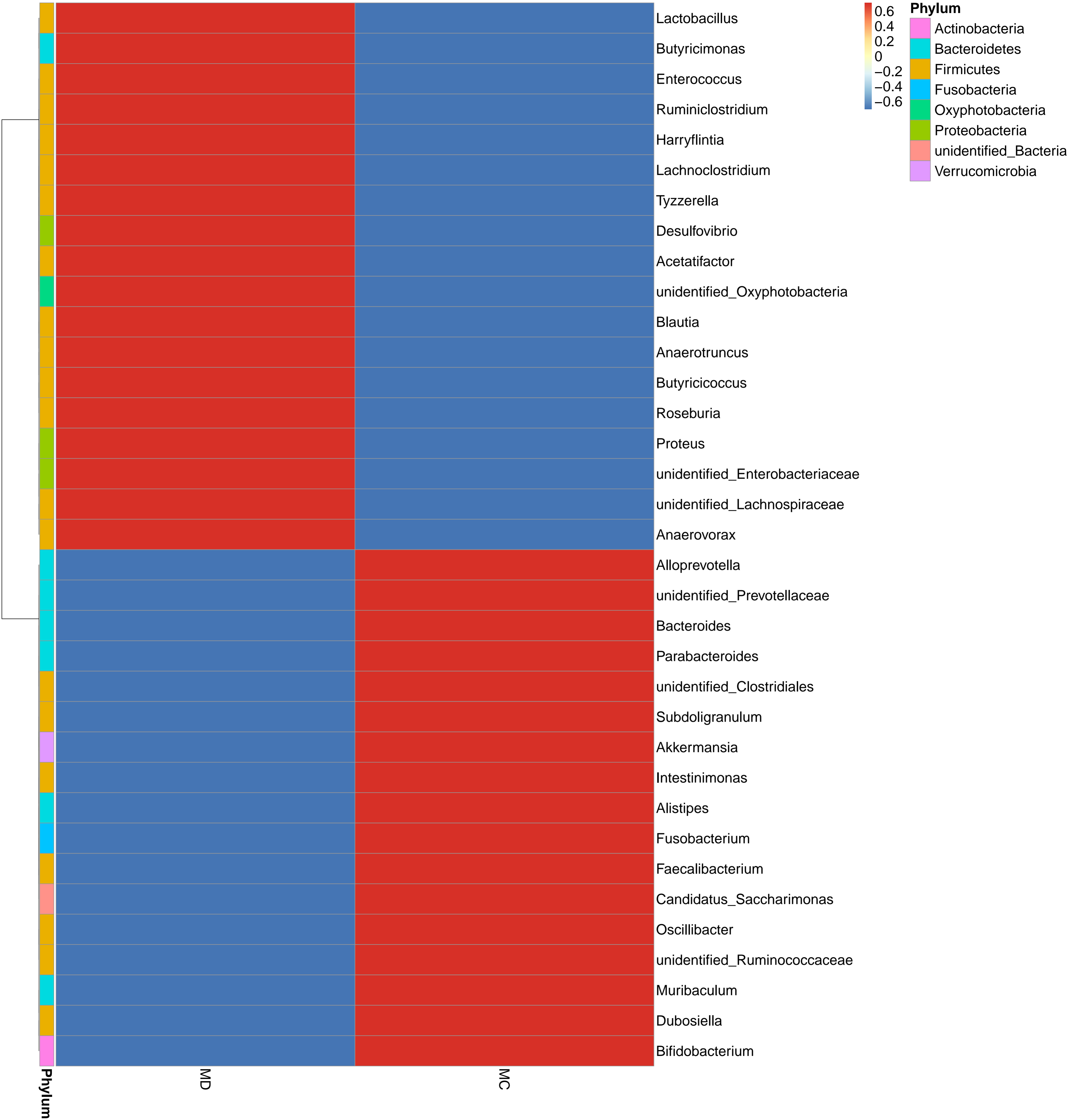

Supplement: Supplementary file 1 [file Data_Sheet_1.zip › P101SC18090073-01-B1-3-4_result/02.OTUanalysis/taxa_heatmap/cluster_group/cluster.g.pdf]

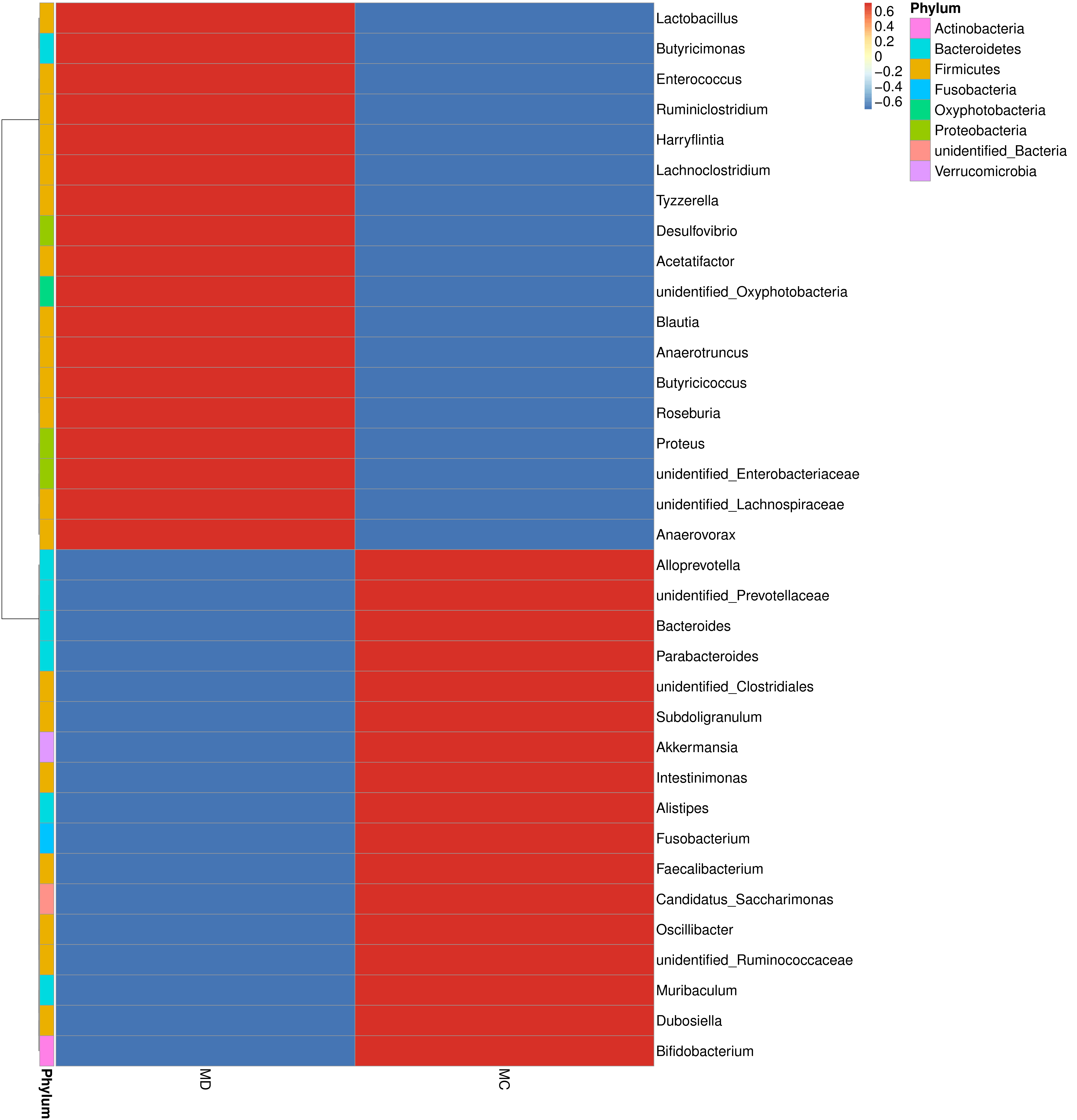

Supplement: Supplementary file 1 [file Data_Sheet_1.zip › P101SC18090073-01-B1-3-4_result/02.OTUanalysis/taxa_heatmap/cluster_group/cluster.g.png]

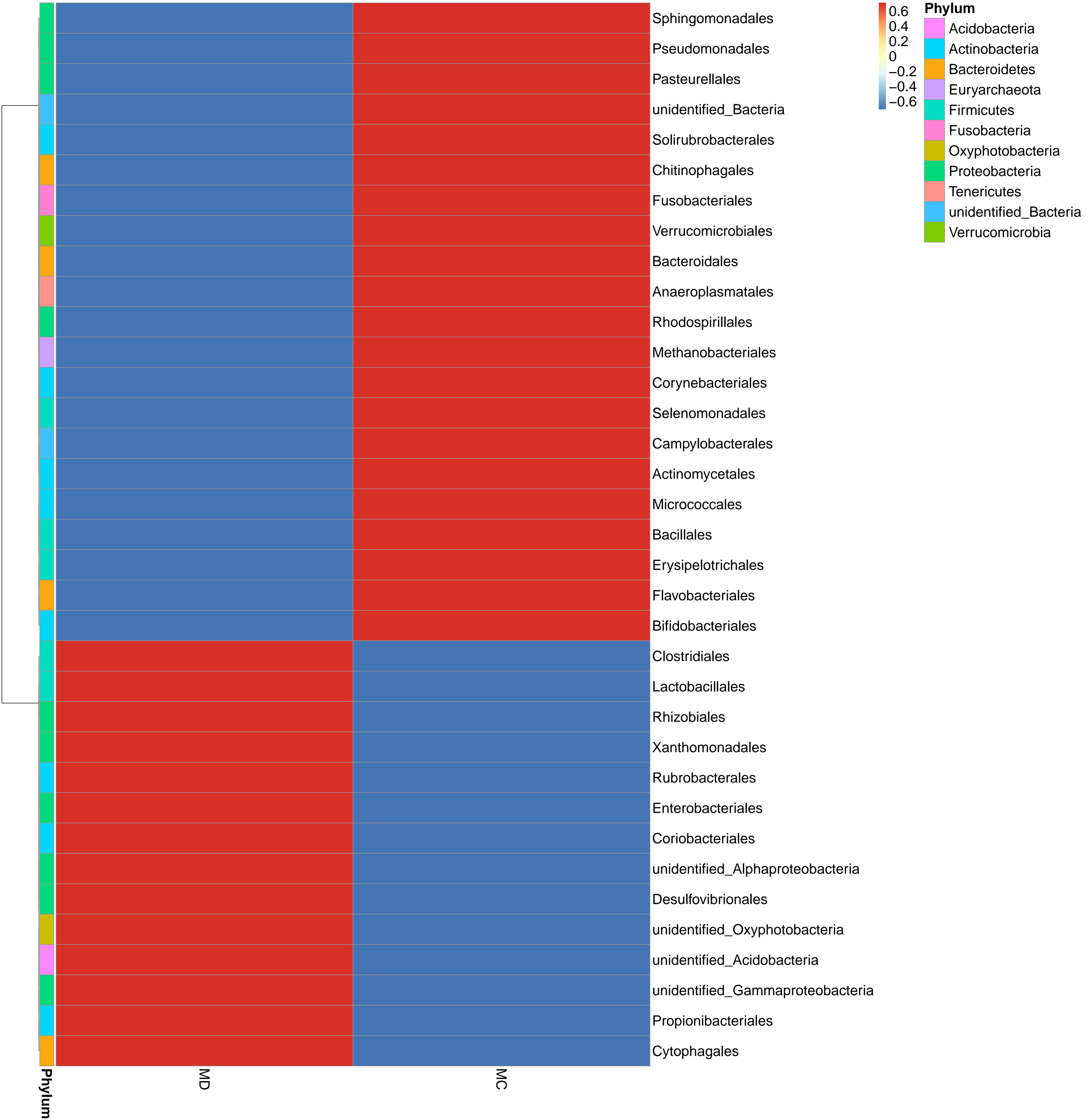

Supplement: Supplementary file 1 [file Data_Sheet_1.zip › P101SC18090073-01-B1-3-4_result/02.OTUanalysis/taxa_heatmap/cluster_group/cluster.o.pdf]

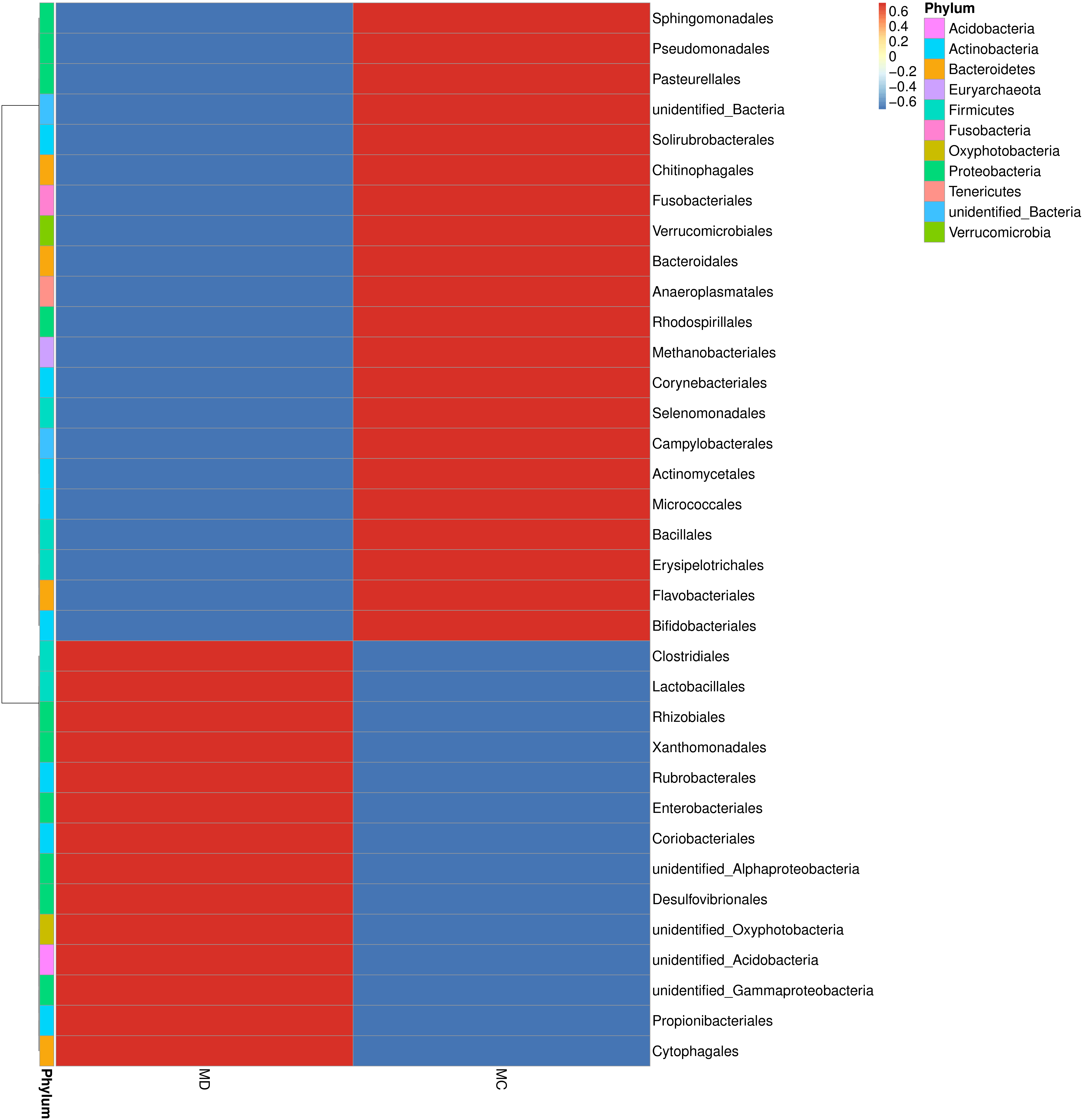

Supplement: Supplementary file 1 [file Data_Sheet_1.zip › P101SC18090073-01-B1-3-4_result/02.OTUanalysis/taxa_heatmap/cluster_group/cluster.o.png]

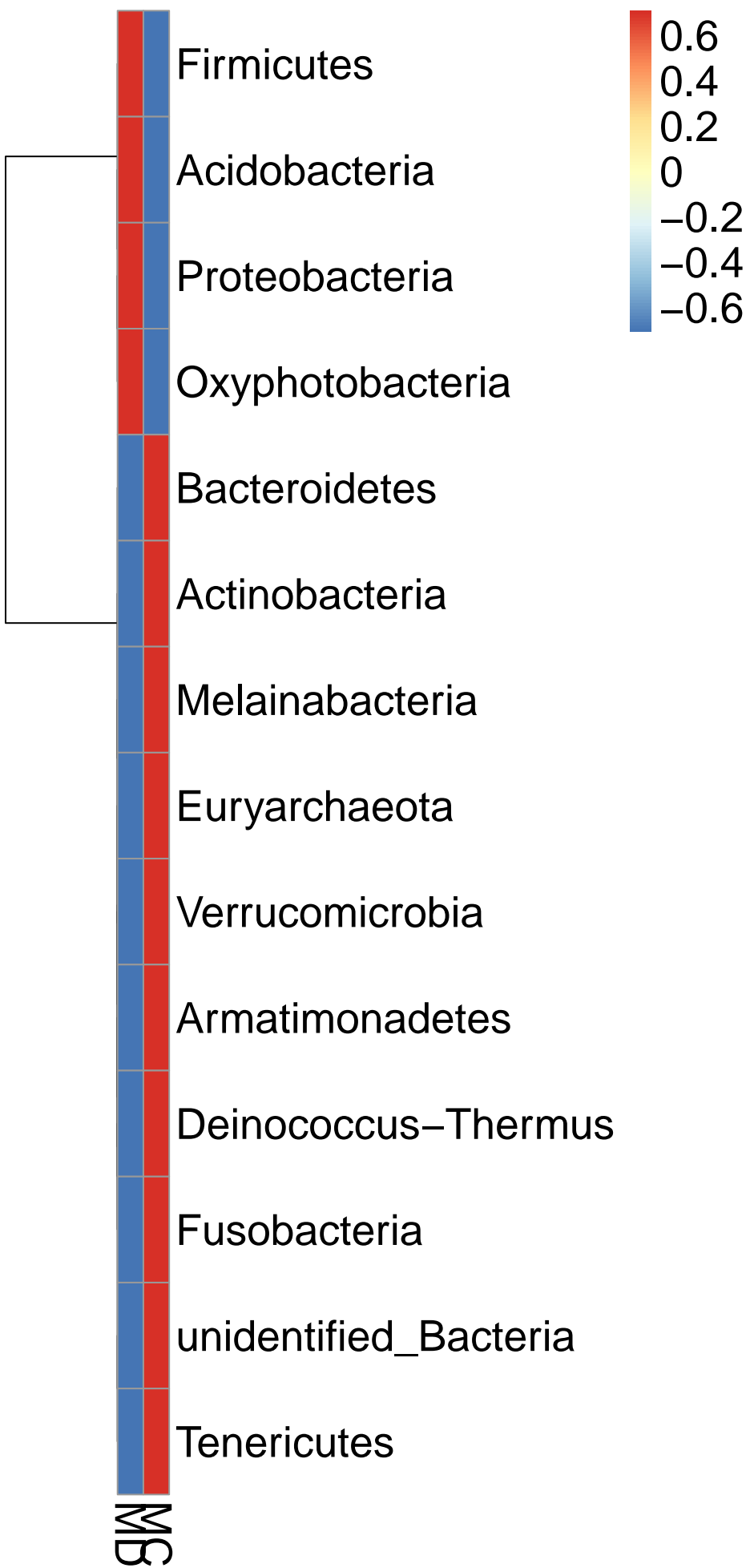

Supplement: Supplementary file 1 [file Data_Sheet_1.zip › P101SC18090073-01-B1-3-4_result/02.OTUanalysis/taxa_heatmap/cluster_group/cluster.p.pdf]

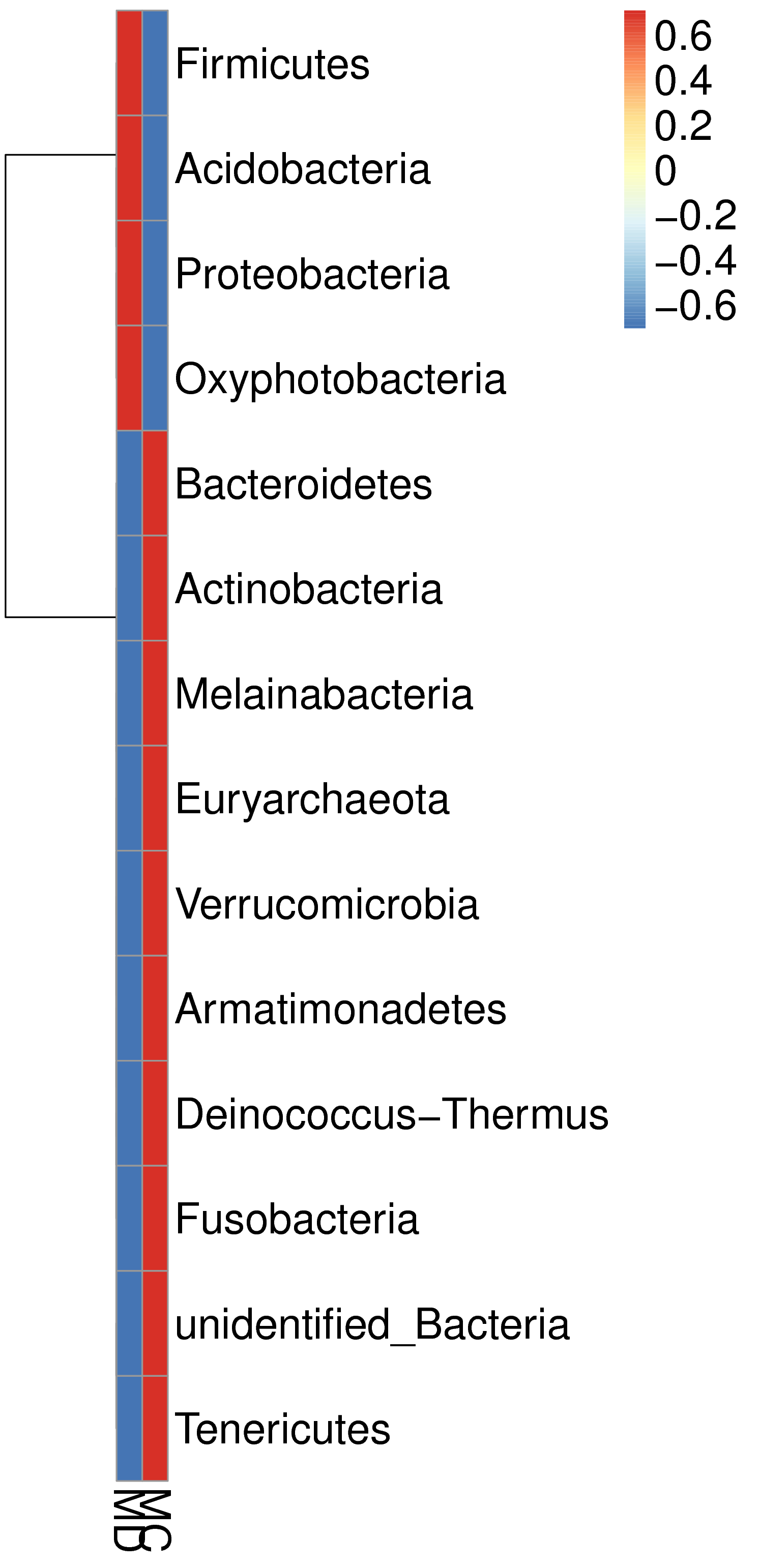

Supplement: Supplementary file 1 [file Data_Sheet_1.zip › P101SC18090073-01-B1-3-4_result/02.OTUanalysis/taxa_heatmap/cluster_group/cluster.p.png]

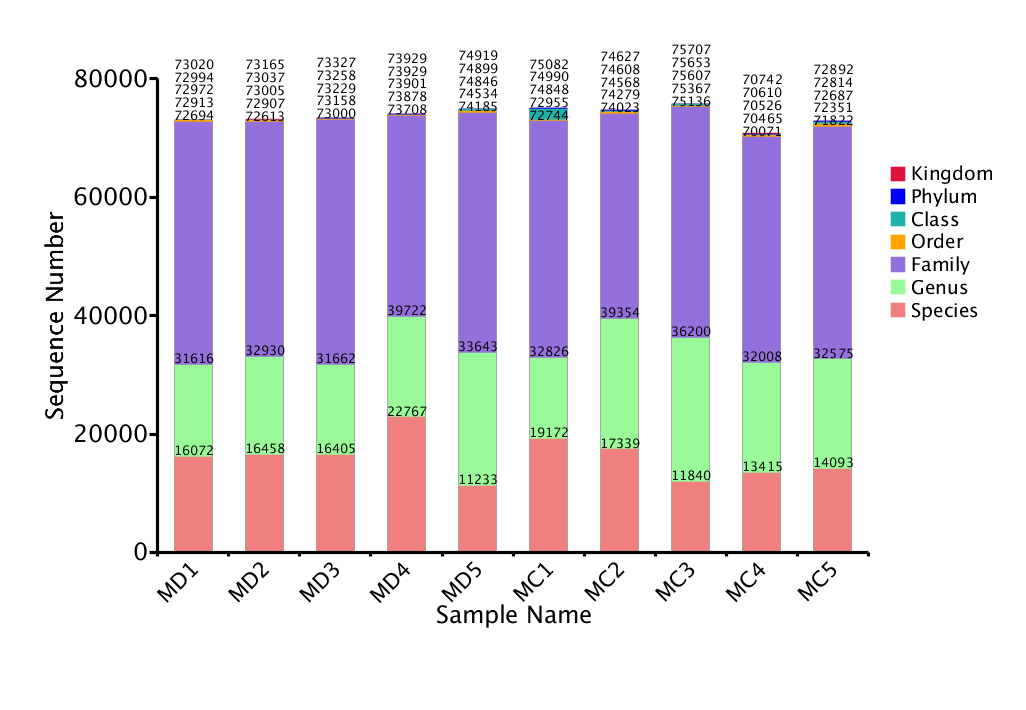

Supplement: Supplementary file 1 [file Data_Sheet_1.zip › P101SC18090073-01-B1-3-4_result/02.OTUanalysis/taxa_stat/Classified_stat.png]

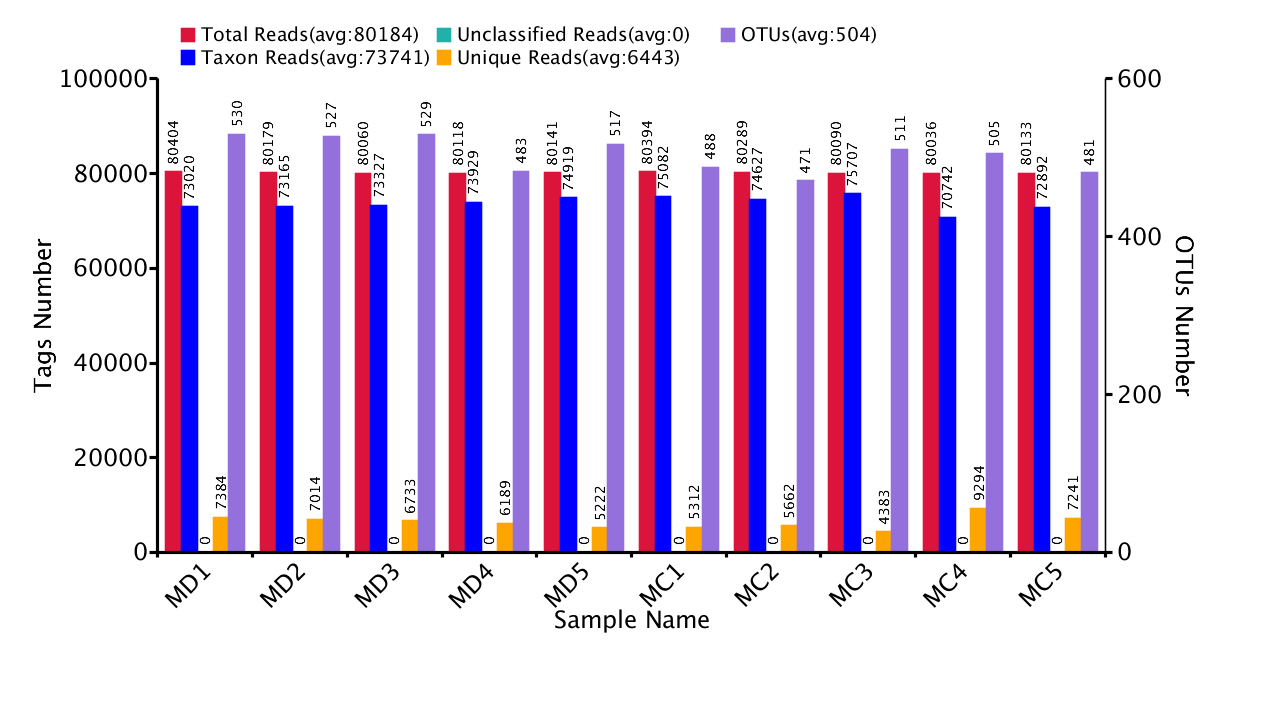

Supplement: Supplementary file 1 [file Data_Sheet_1.zip › P101SC18090073-01-B1-3-4_result/02.OTUanalysis/taxa_stat/Sample_stats-OTUs_dis.png]

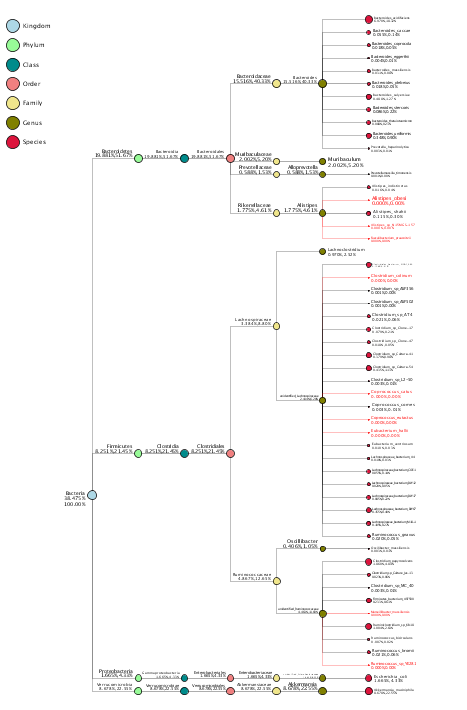

Supplement: Supplementary file 1 [file Data_Sheet_1.zip › P101SC18090073-01-B1-3-4_result/02.OTUanalysis/taxa_tree/MC1.taxtree.png]

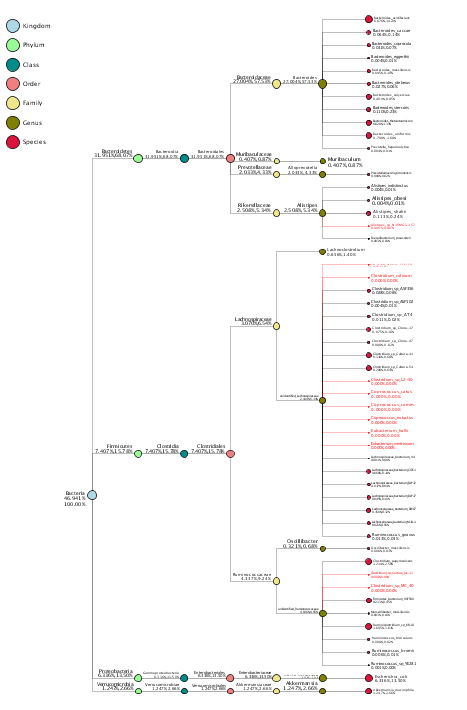

Supplement: Supplementary file 1 [file Data_Sheet_1.zip › P101SC18090073-01-B1-3-4_result/02.OTUanalysis/taxa_tree/MC2.taxtree.png]

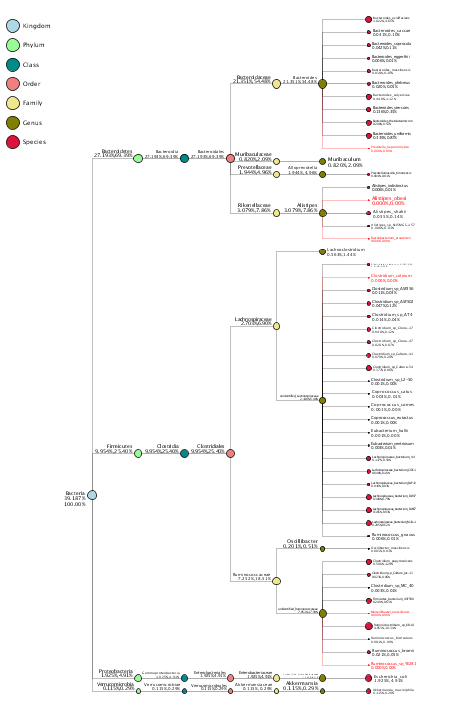

Supplement: Supplementary file 1 [file Data_Sheet_1.zip › P101SC18090073-01-B1-3-4_result/02.OTUanalysis/taxa_tree/MC3.taxtree.png]

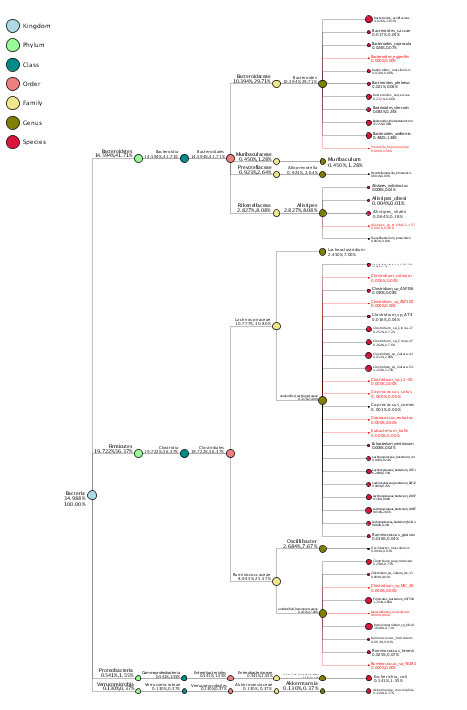

Supplement: Supplementary file 1 [file Data_Sheet_1.zip › P101SC18090073-01-B1-3-4_result/02.OTUanalysis/taxa_tree/MC4.taxtree.png]

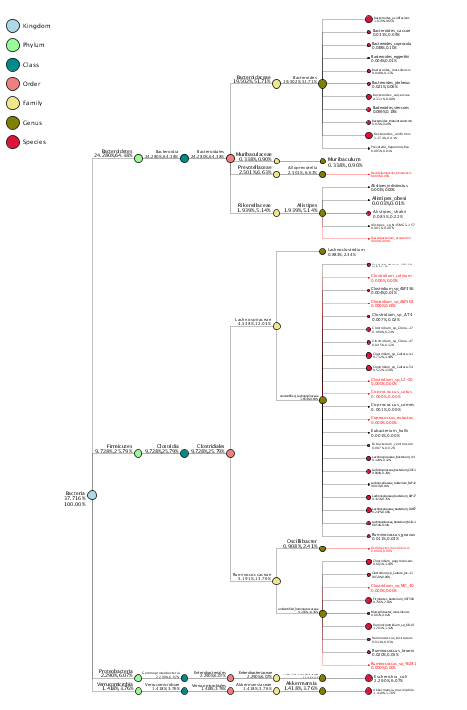

Supplement: Supplementary file 1 [file Data_Sheet_1.zip › P101SC18090073-01-B1-3-4_result/02.OTUanalysis/taxa_tree/MC5.taxtree.png]

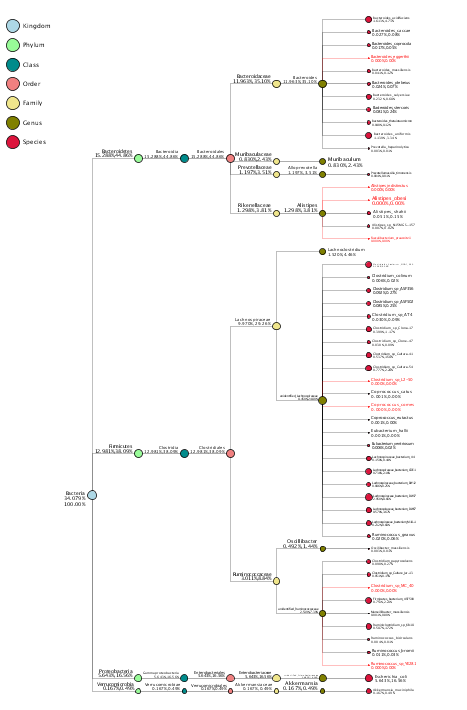

Supplement: Supplementary file 1 [file Data_Sheet_1.zip › P101SC18090073-01-B1-3-4_result/02.OTUanalysis/taxa_tree/MD1.taxtree.png]

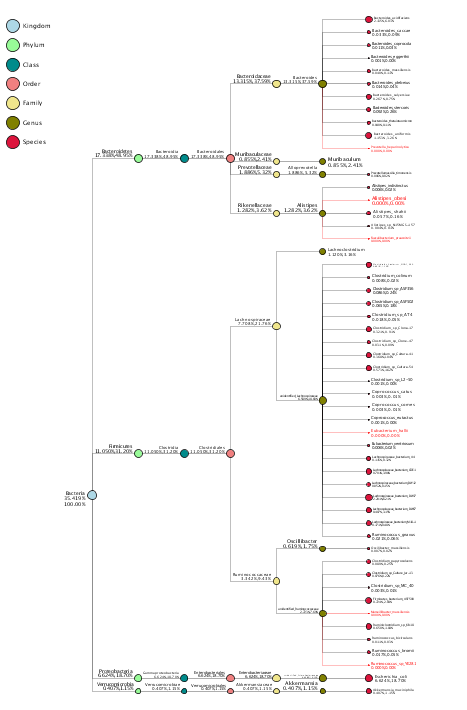

Supplement: Supplementary file 1 [file Data_Sheet_1.zip › P101SC18090073-01-B1-3-4_result/02.OTUanalysis/taxa_tree/MD2.taxtree.png]

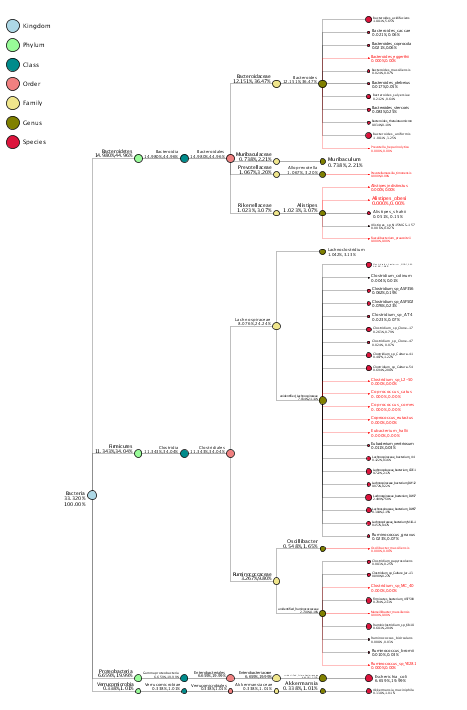

Supplement: Supplementary file 1 [file Data_Sheet_1.zip › P101SC18090073-01-B1-3-4_result/02.OTUanalysis/taxa_tree/MD3.taxtree.png]

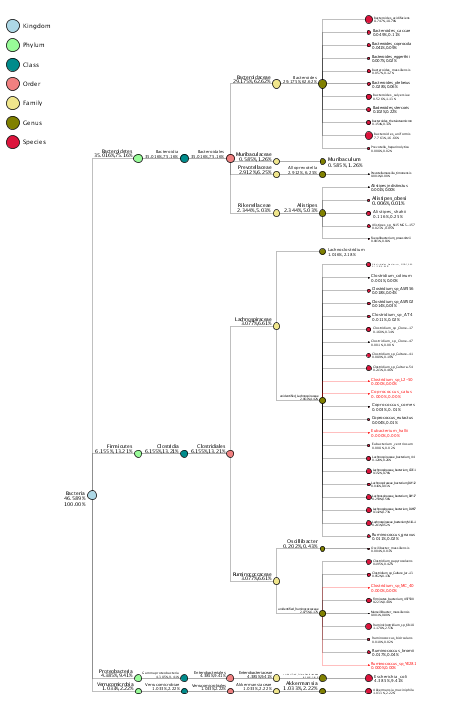

Supplement: Supplementary file 1 [file Data_Sheet_1.zip › P101SC18090073-01-B1-3-4_result/02.OTUanalysis/taxa_tree/MD4.taxtree.png]

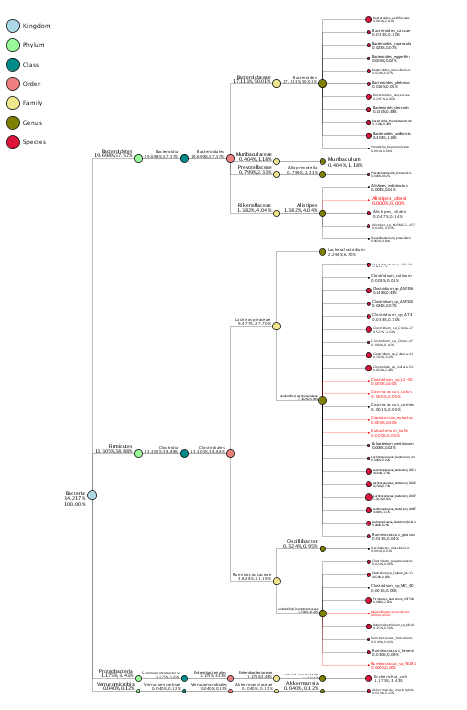

Supplement: Supplementary file 1 [file Data_Sheet_1.zip › P101SC18090073-01-B1-3-4_result/02.OTUanalysis/taxa_tree/MD5.taxtree.png]

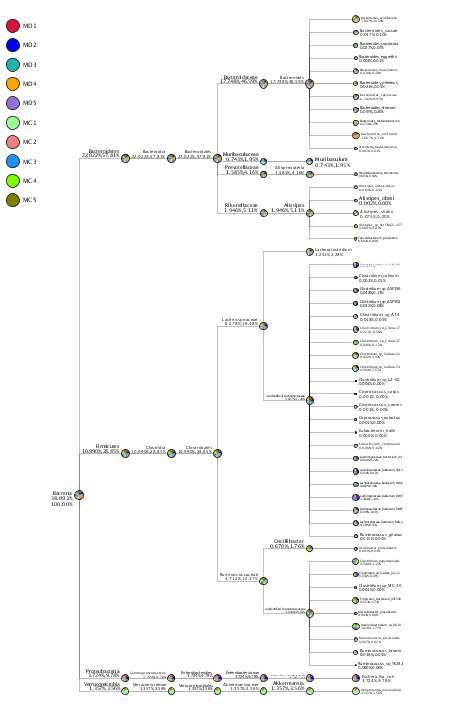

Supplement: Supplementary file 1 [file Data_Sheet_1.zip › P101SC18090073-01-B1-3-4_result/02.OTUanalysis/taxa_tree/all.taxtree.png]

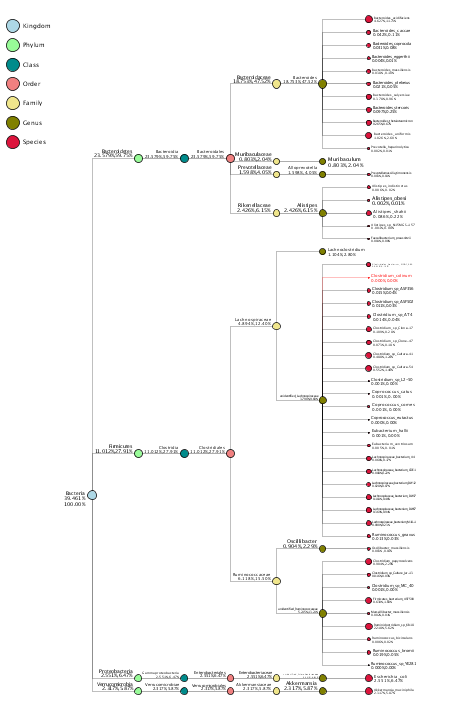

Supplement: Supplementary file 1 [file Data_Sheet_1.zip › P101SC18090073-01-B1-3-4_result/02.OTUanalysis/taxa_tree_group/MC.taxtree.png]

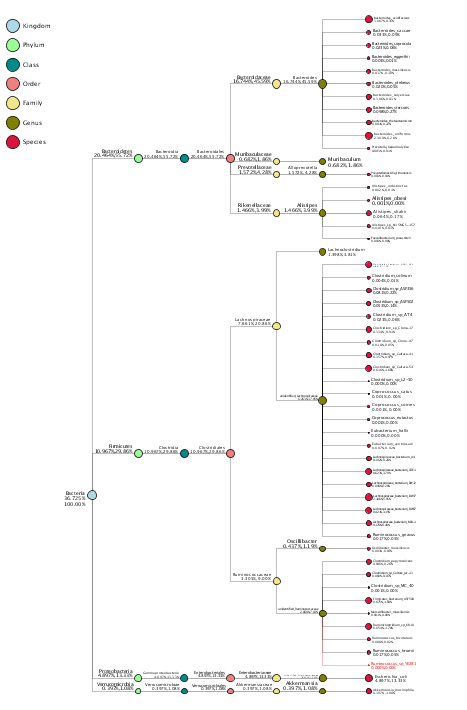

Supplement: Supplementary file 1 [file Data_Sheet_1.zip › P101SC18090073-01-B1-3-4_result/02.OTUanalysis/taxa_tree_group/MD.taxtree.png]

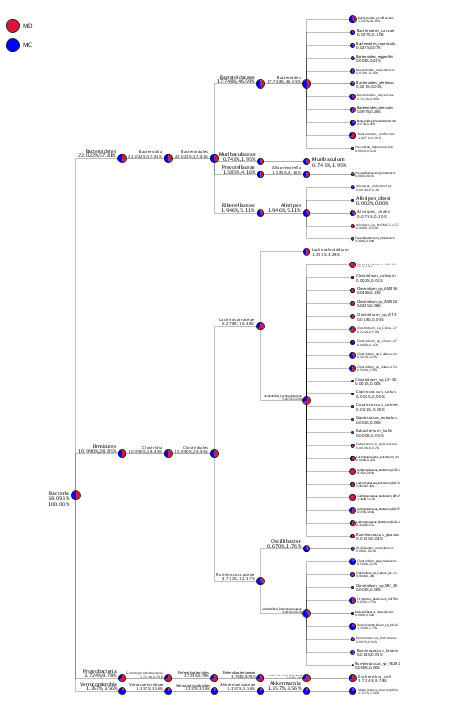

Supplement: Supplementary file 1 [file Data_Sheet_1.zip › P101SC18090073-01-B1-3-4_result/02.OTUanalysis/taxa_tree_group/all.taxtree.png]

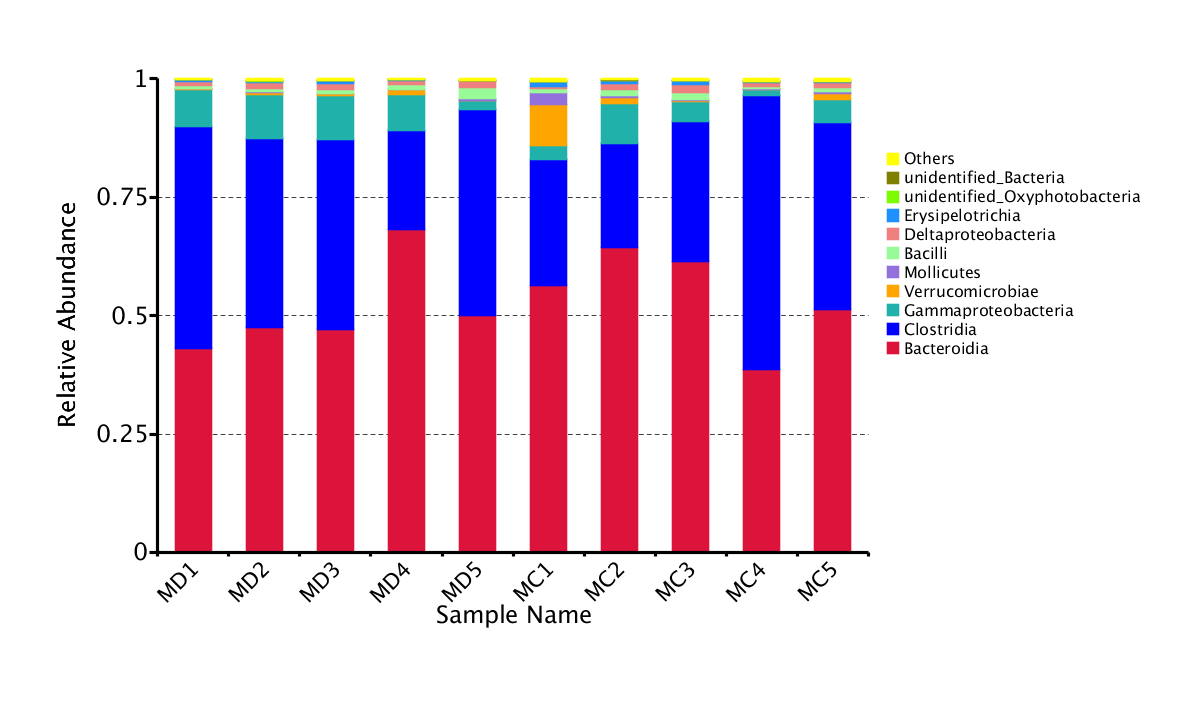

Supplement: Supplementary file 1 [file Data_Sheet_1.zip › P101SC18090073-01-B1-3-4_result/02.OTUanalysis/top10/class/c10.relative.dis.png]

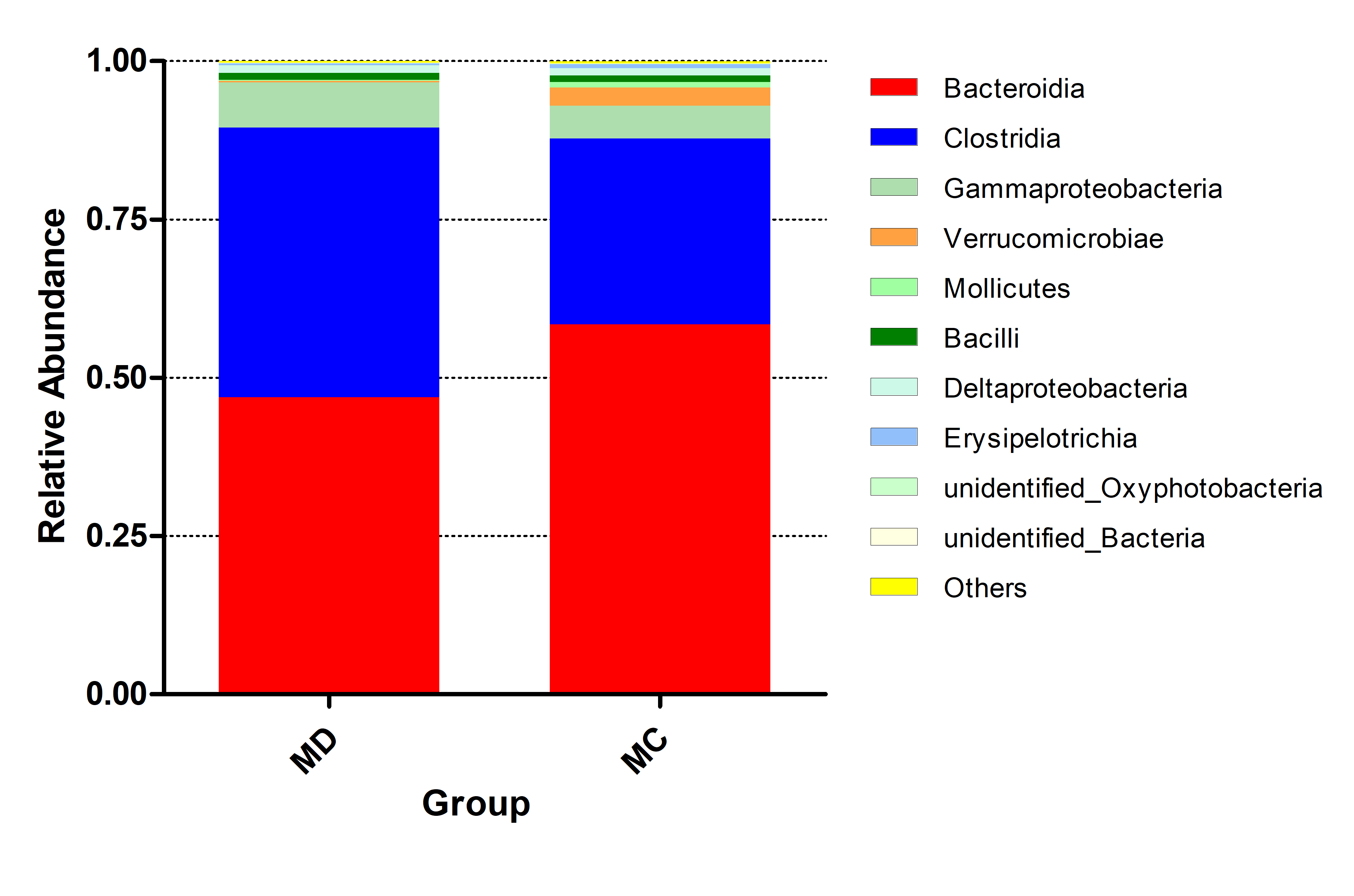

Supplement: Supplementary file 1 [file Data_Sheet_1.zip › P101SC18090073-01-B1-3-4_result/02.OTUanalysis/top10/class/class top10-20181123.jpg]

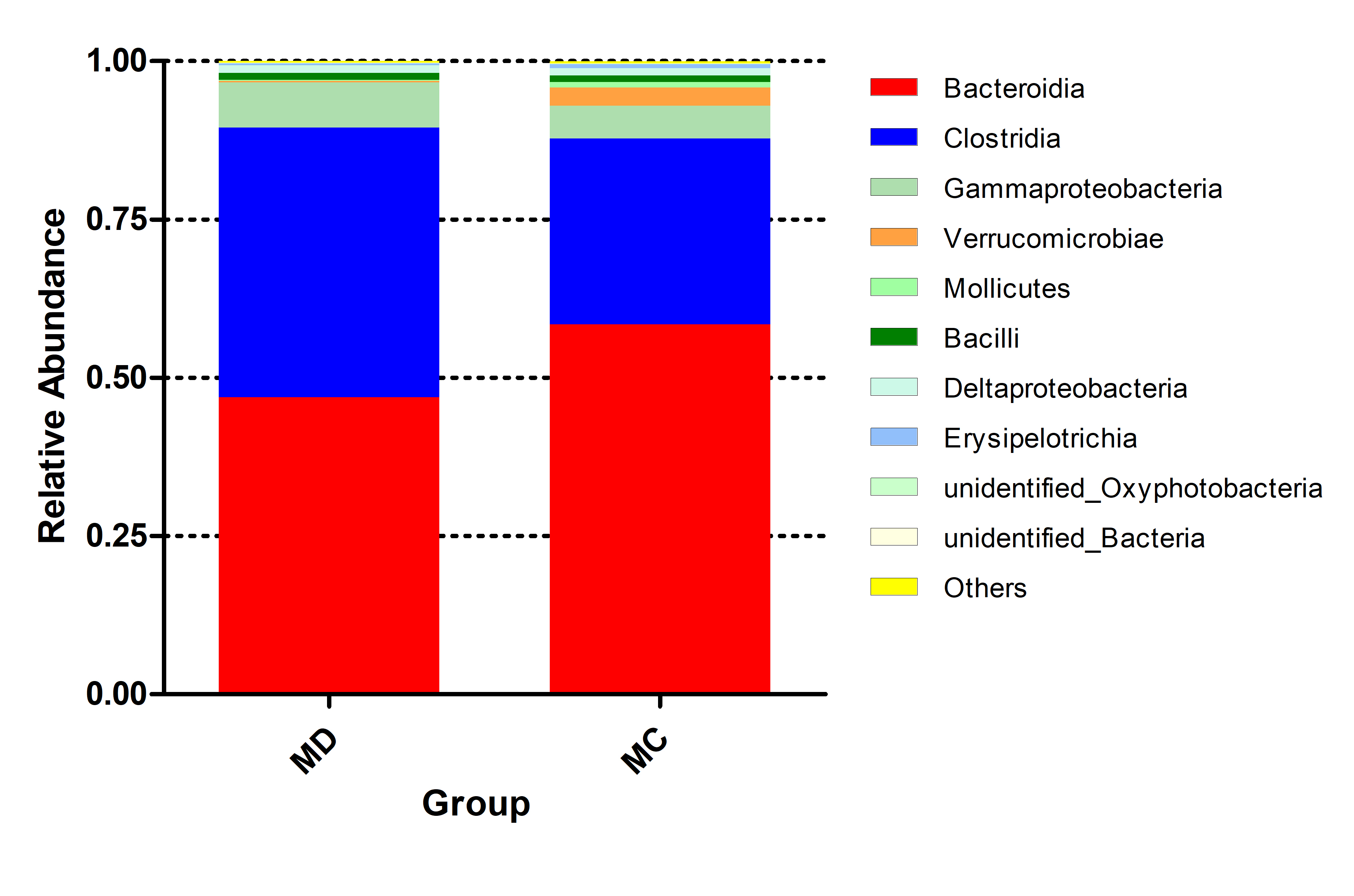

Supplement: Supplementary file 1 [file Data_Sheet_1.zip › P101SC18090073-01-B1-3-4_result/02.OTUanalysis/top10/class/class top10.jpg]

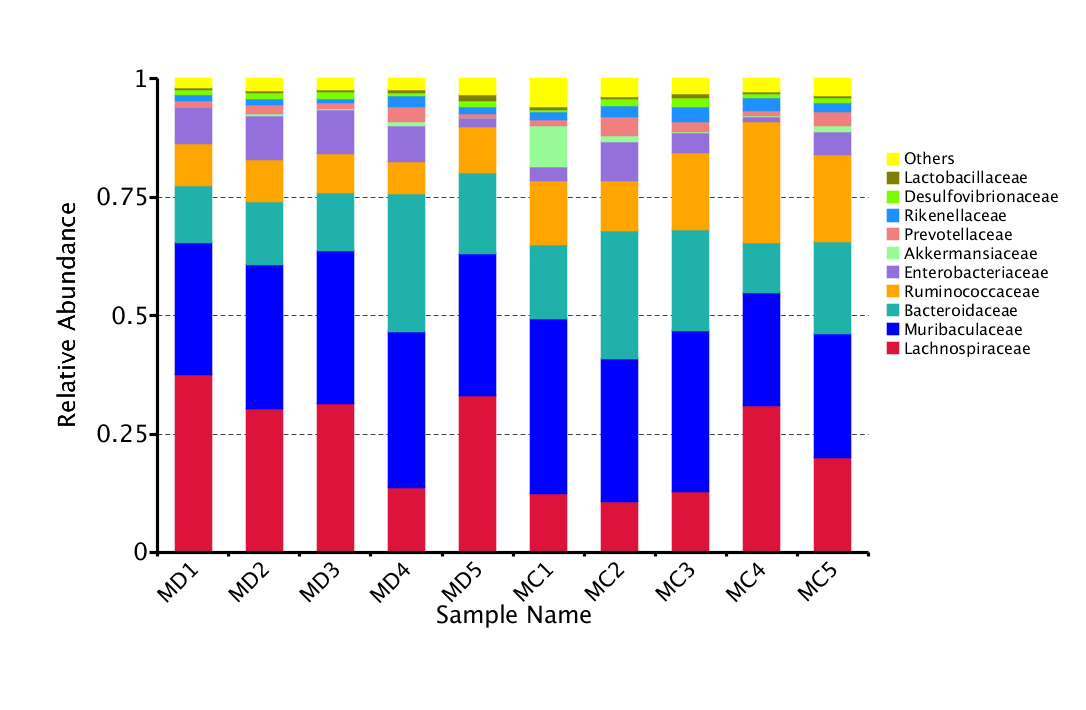

Supplement: Supplementary file 1 [file Data_Sheet_1.zip › P101SC18090073-01-B1-3-4_result/02.OTUanalysis/top10/family/f10.relative.dis.png]

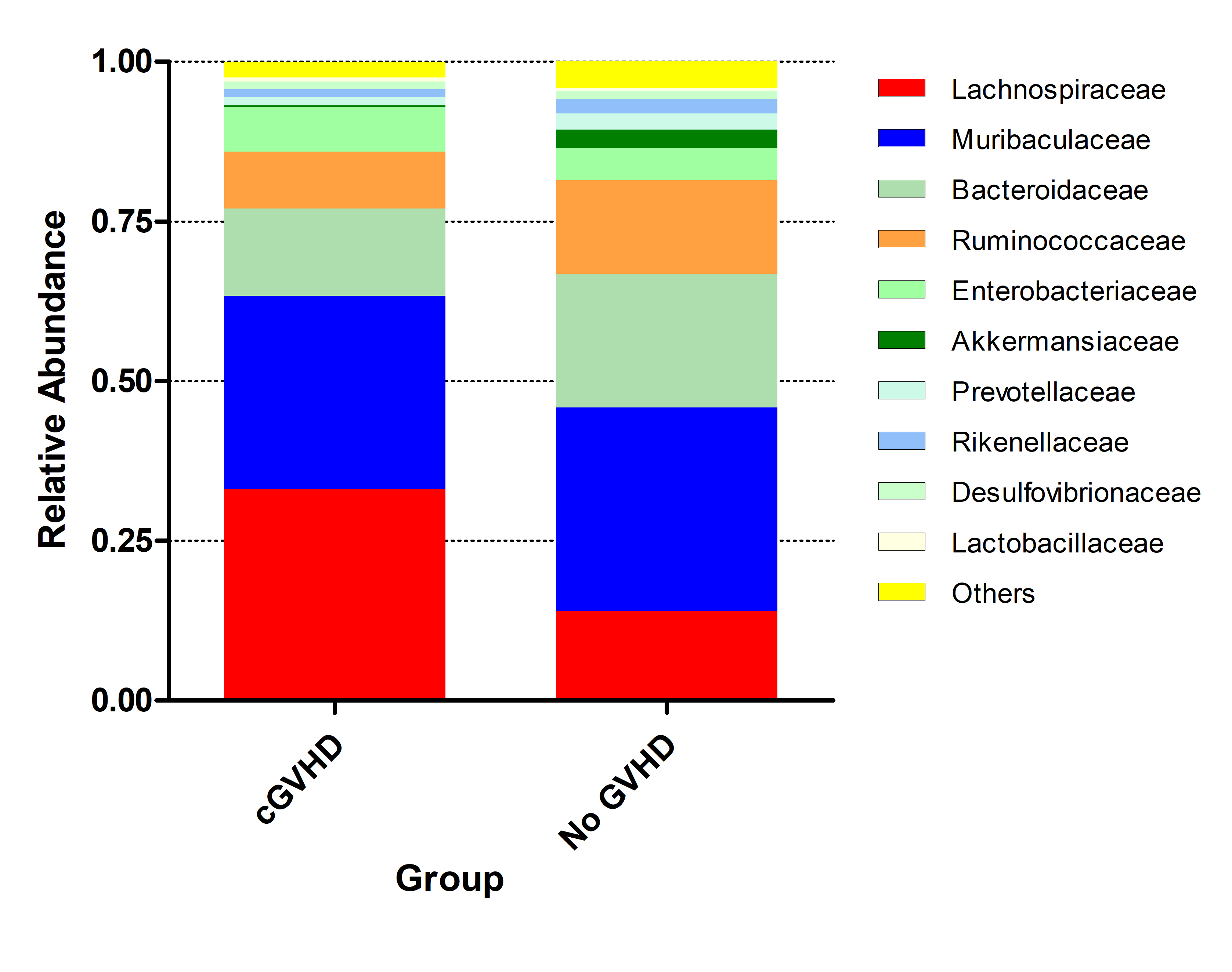

Supplement: Supplementary file 1 [file Data_Sheet_1.zip › P101SC18090073-01-B1-3-4_result/02.OTUanalysis/top10/family/family top 10.jpg]

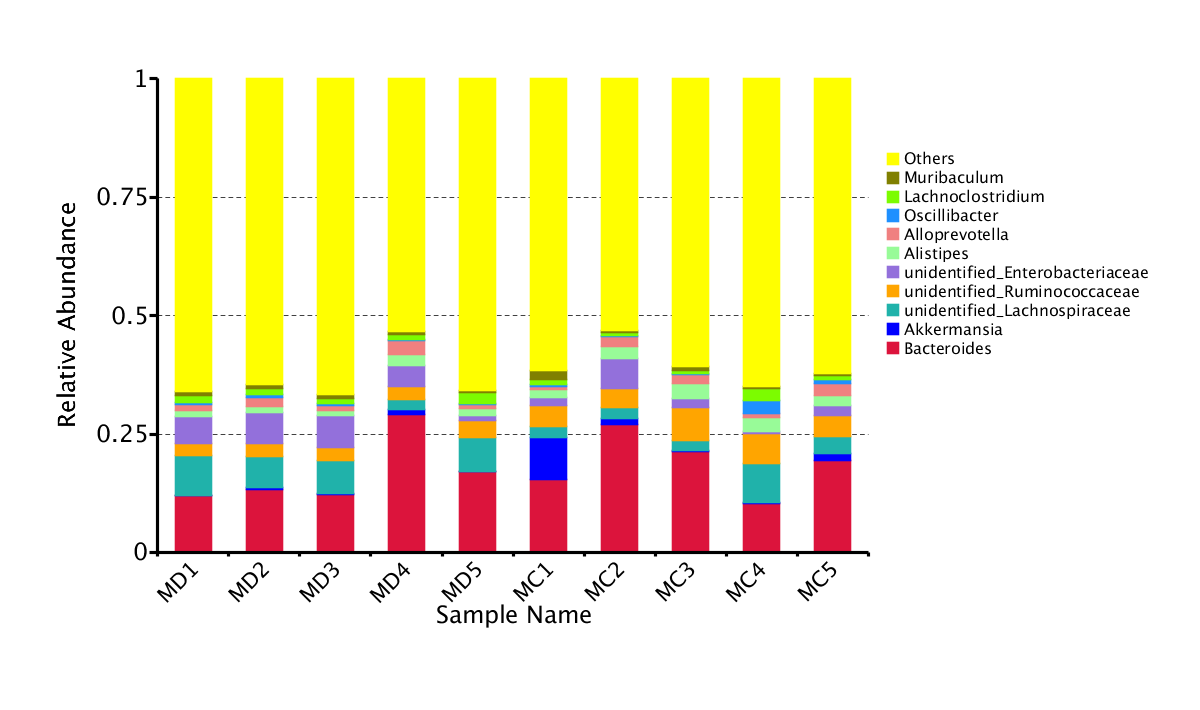

Supplement: Supplementary file 1 [file Data_Sheet_1.zip › P101SC18090073-01-B1-3-4_result/02.OTUanalysis/top10/genus/g10.relative.dis.png]

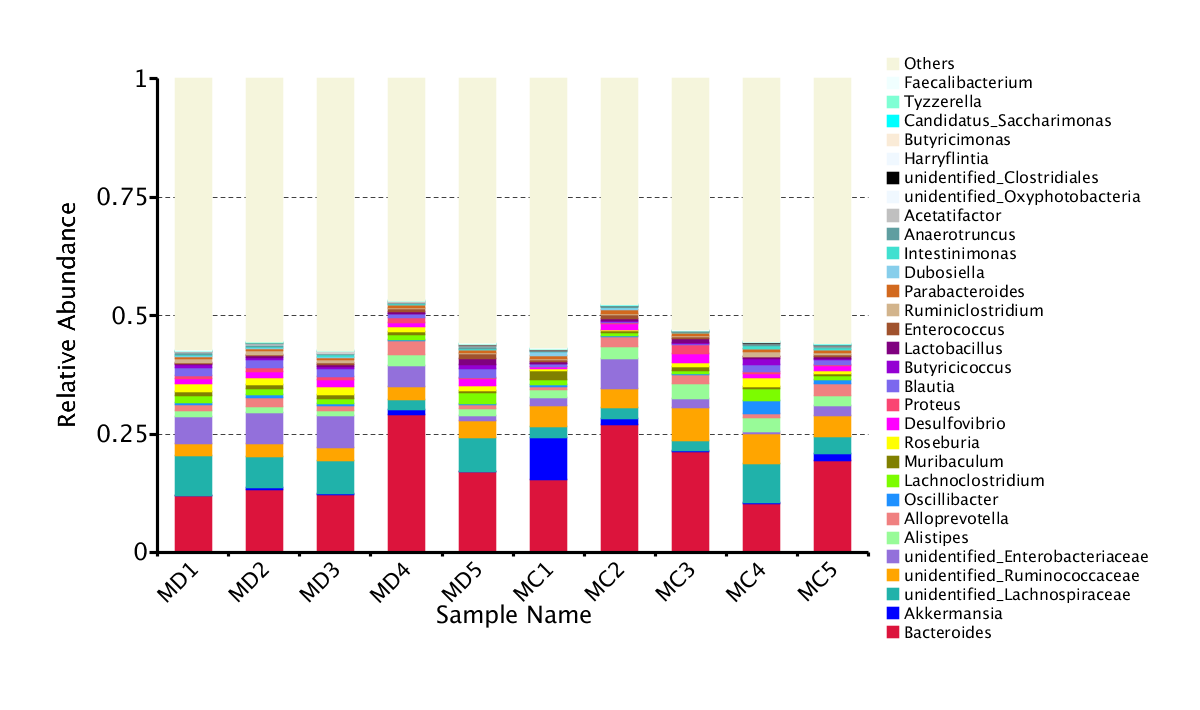

Supplement: Supplementary file 1 [file Data_Sheet_1.zip › P101SC18090073-01-B1-3-4_result/02.OTUanalysis/top10/genus/g30.relative.dis.png]

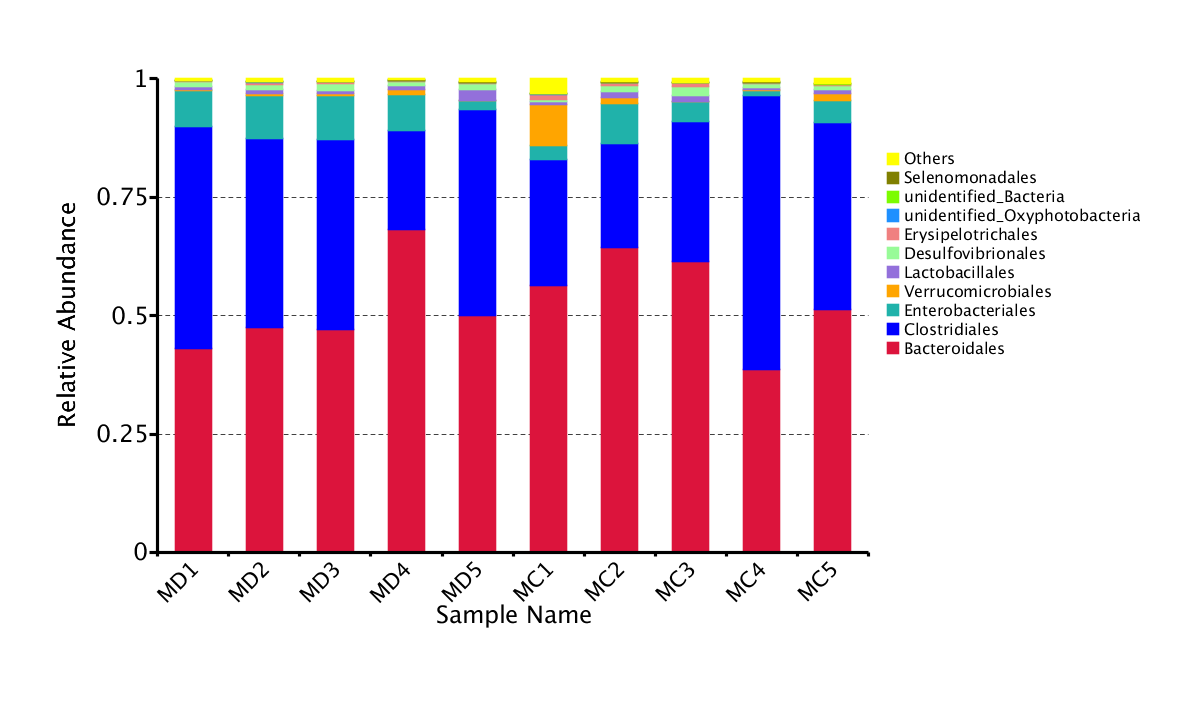

Supplement: Supplementary file 1 [file Data_Sheet_1.zip › P101SC18090073-01-B1-3-4_result/02.OTUanalysis/top10/order/o10.relative.dis.png]

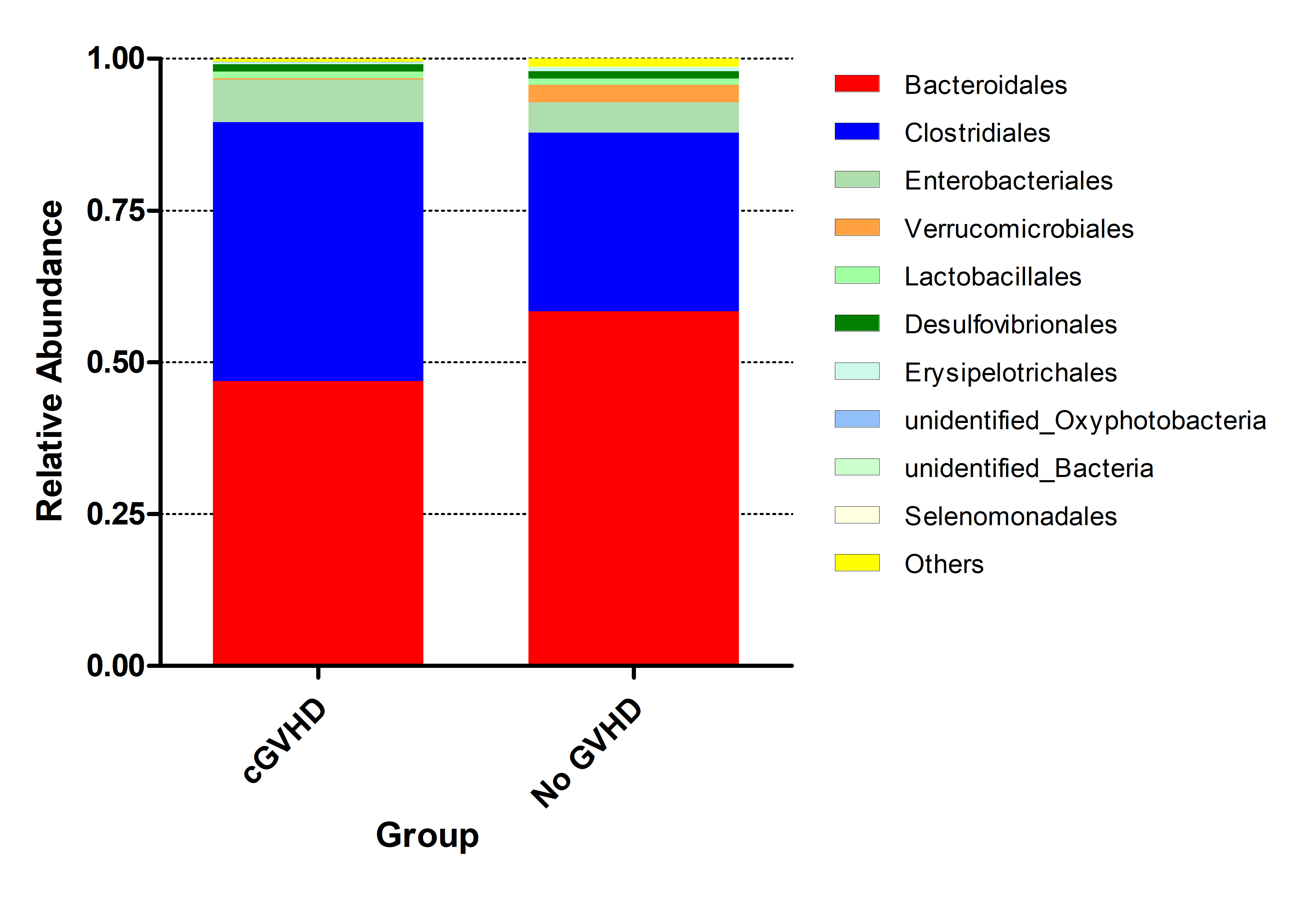

Supplement: Supplementary file 1 [file Data_Sheet_1.zip › P101SC18090073-01-B1-3-4_result/02.OTUanalysis/top10/order/order top10 20181124 group.jpg]

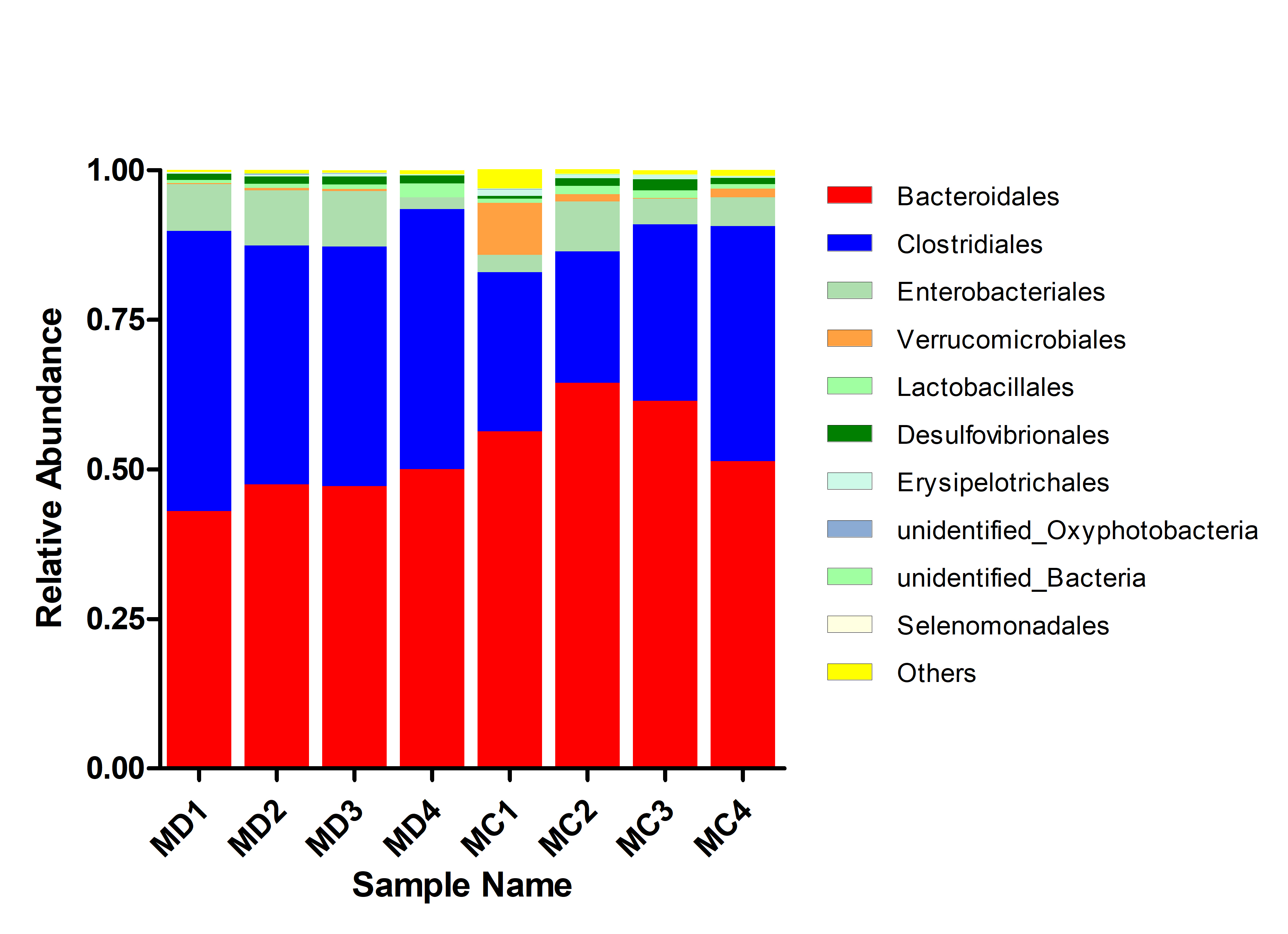

Supplement: Supplementary file 1 [file Data_Sheet_1.zip › P101SC18090073-01-B1-3-4_result/02.OTUanalysis/top10/order/order top10 20181124.jpg]

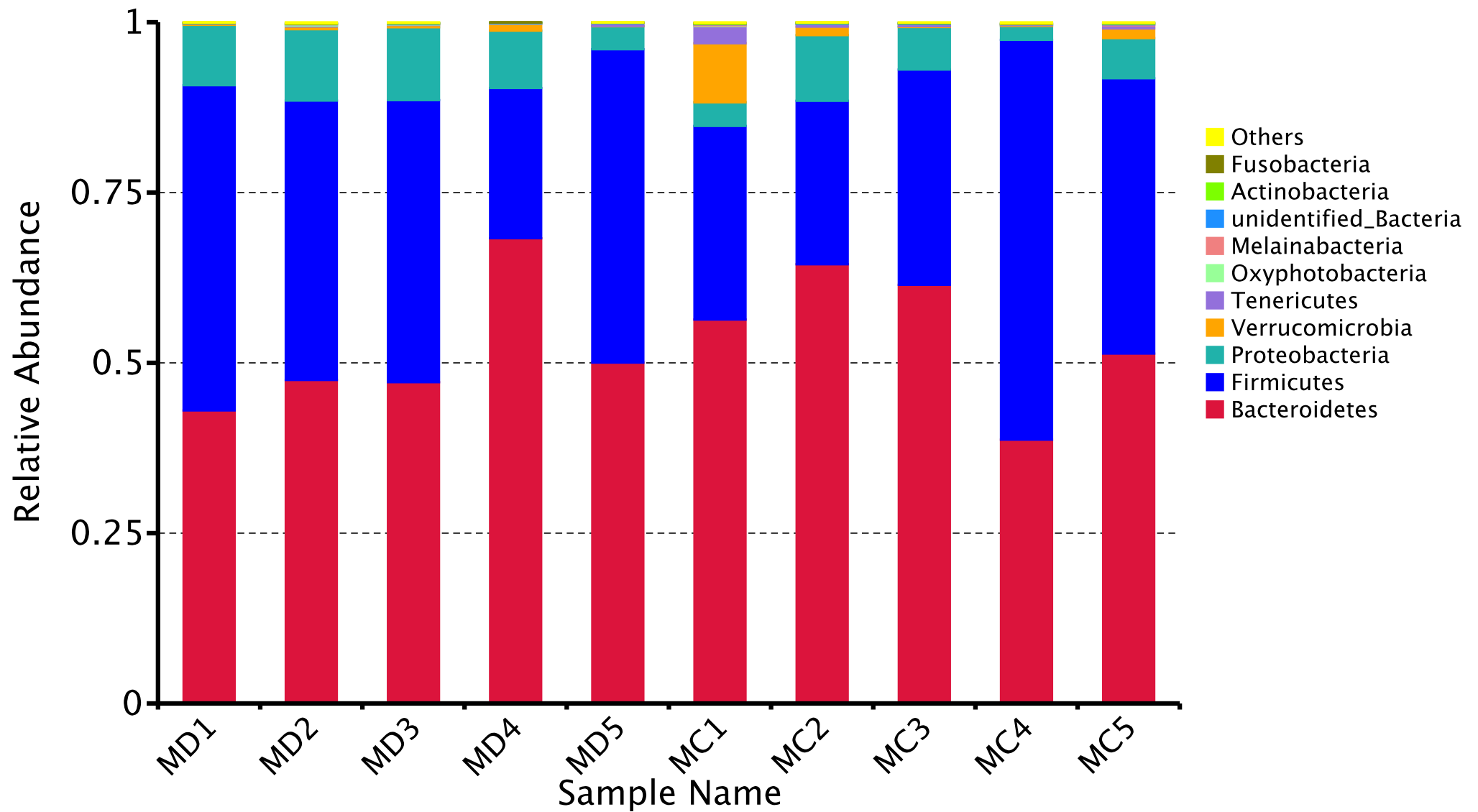

Supplement: Supplementary file 1 [file Data_Sheet_1.zip › P101SC18090073-01-B1-3-4_result/02.OTUanalysis/top10/phylum/p10.relative.dis.pdf]

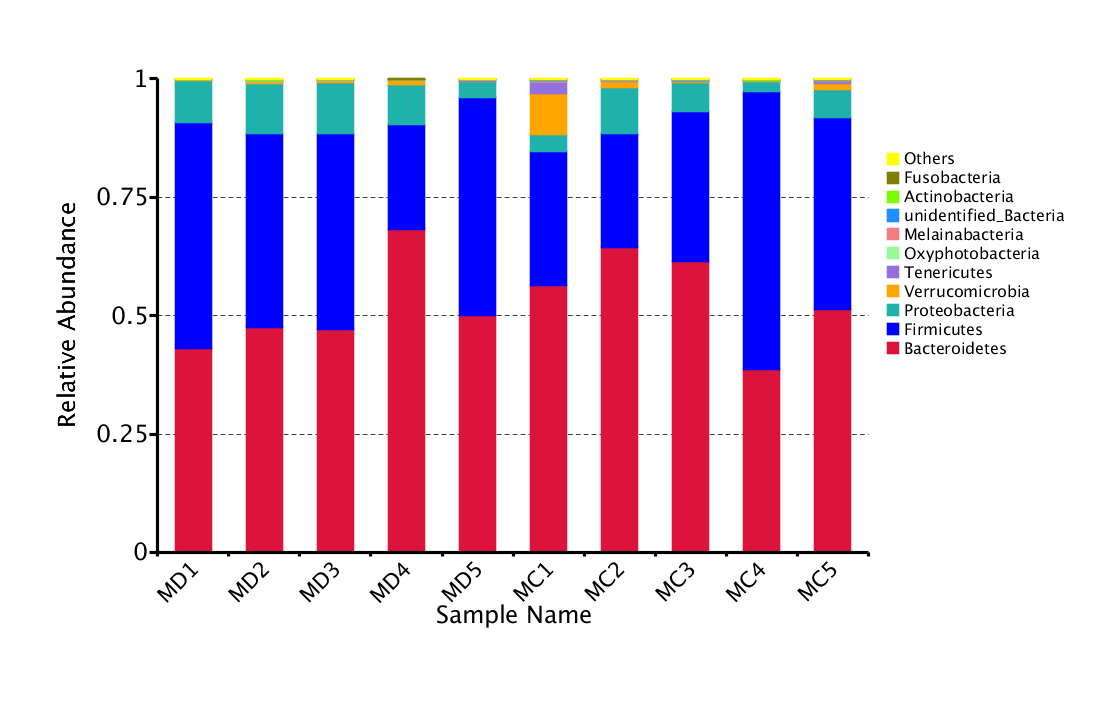

Supplement: Supplementary file 1 [file Data_Sheet_1.zip › P101SC18090073-01-B1-3-4_result/02.OTUanalysis/top10/phylum/p10.relative.dis.png]

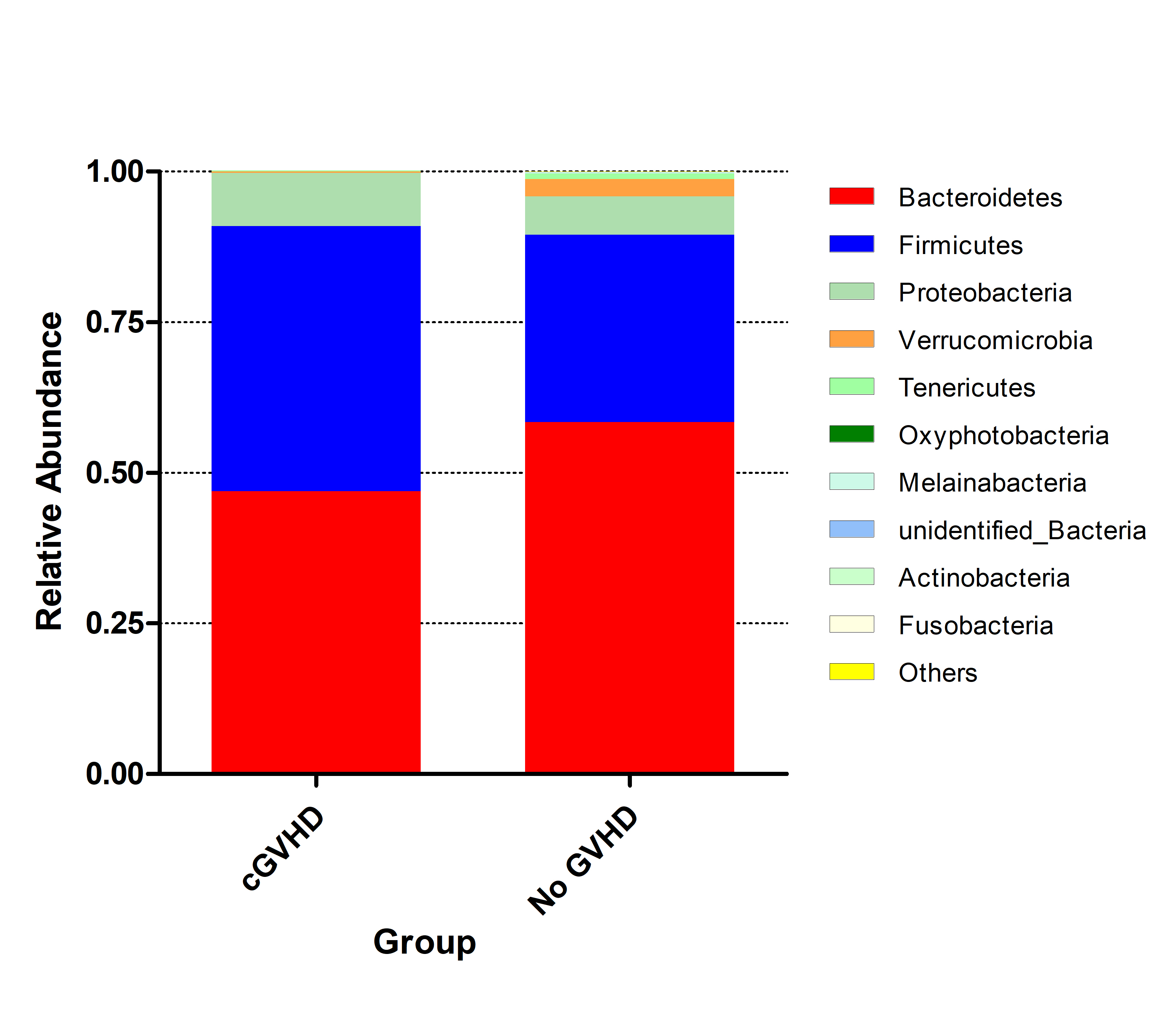

Supplement: Supplementary file 1 [file Data_Sheet_1.zip › P101SC18090073-01-B1-3-4_result/02.OTUanalysis/top10/phylum/phylm top10 group 20181123.jpg]

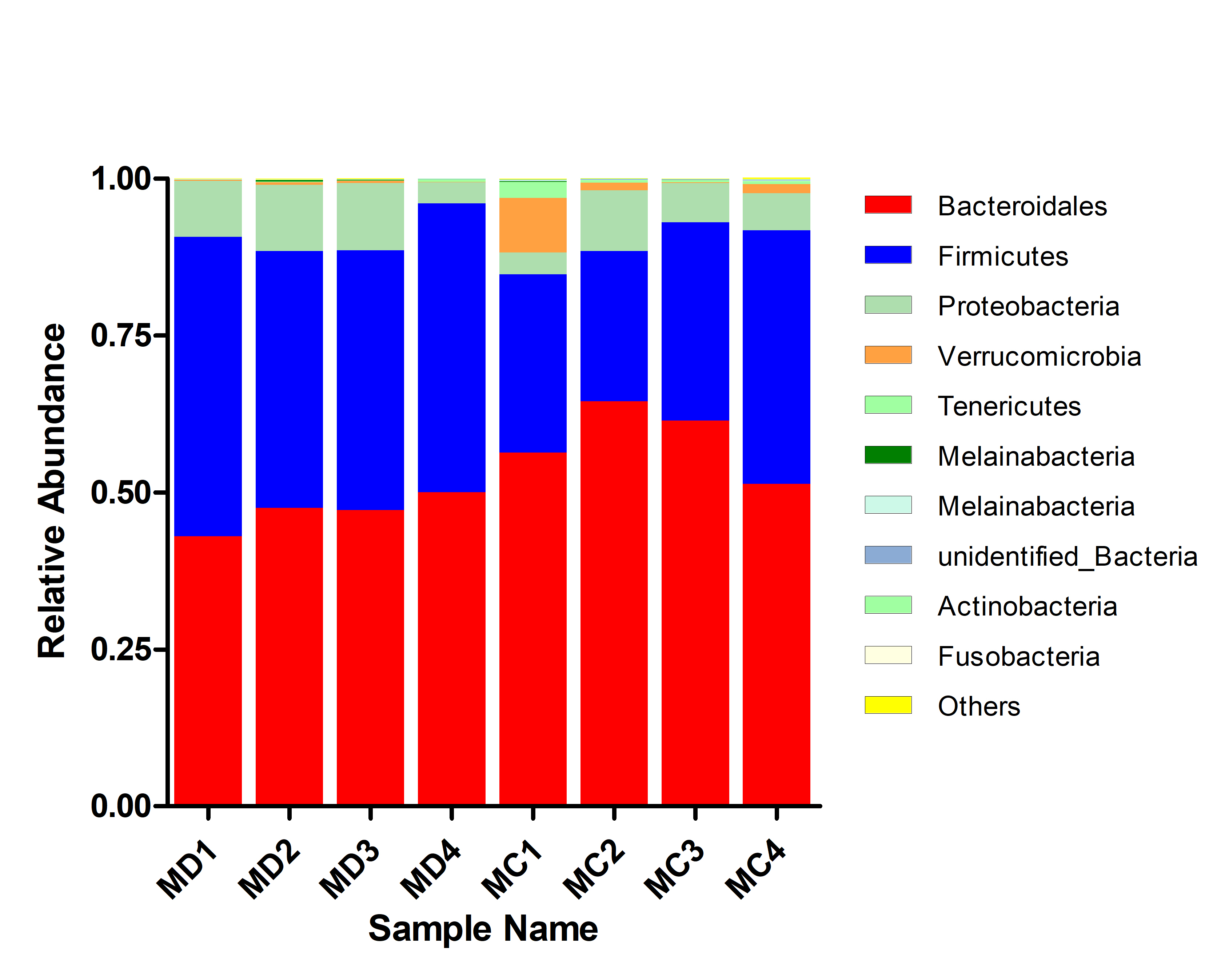

Supplement: Supplementary file 1 [file Data_Sheet_1.zip › P101SC18090073-01-B1-3-4_result/02.OTUanalysis/top10/phylum/phylum top10 20181124 .jpg]

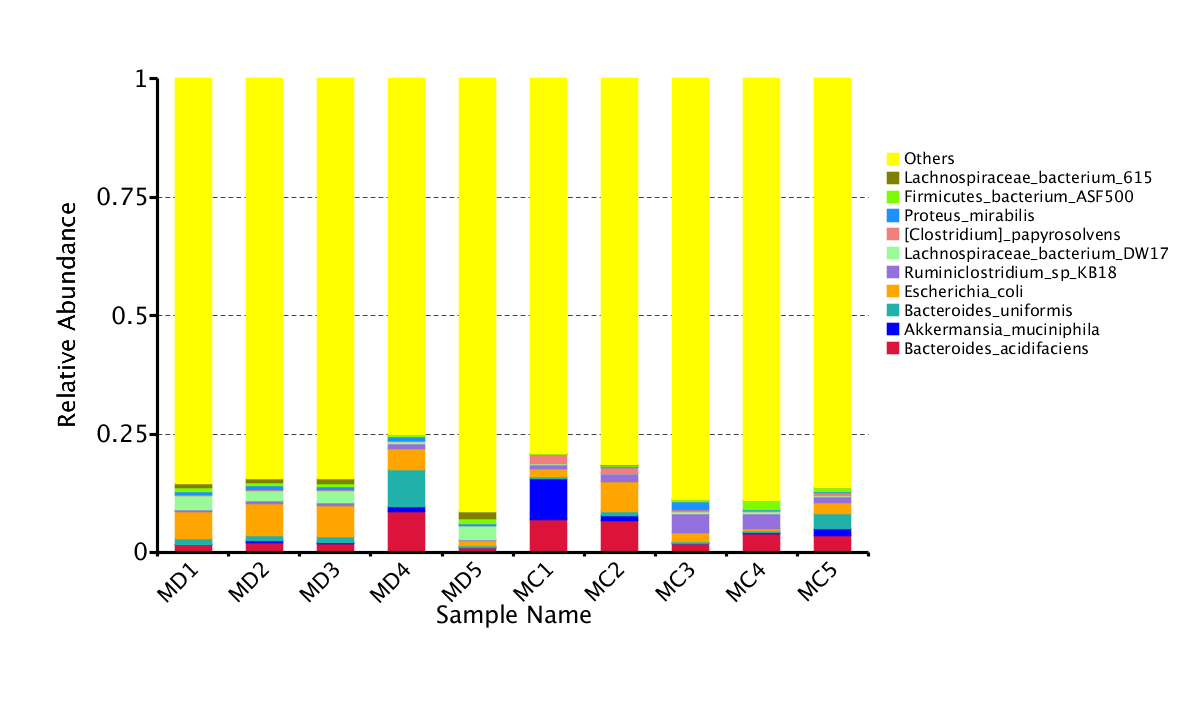

Supplement: Supplementary file 1 [file Data_Sheet_1.zip › P101SC18090073-01-B1-3-4_result/02.OTUanalysis/top10/species/s10.relative.dis.png]

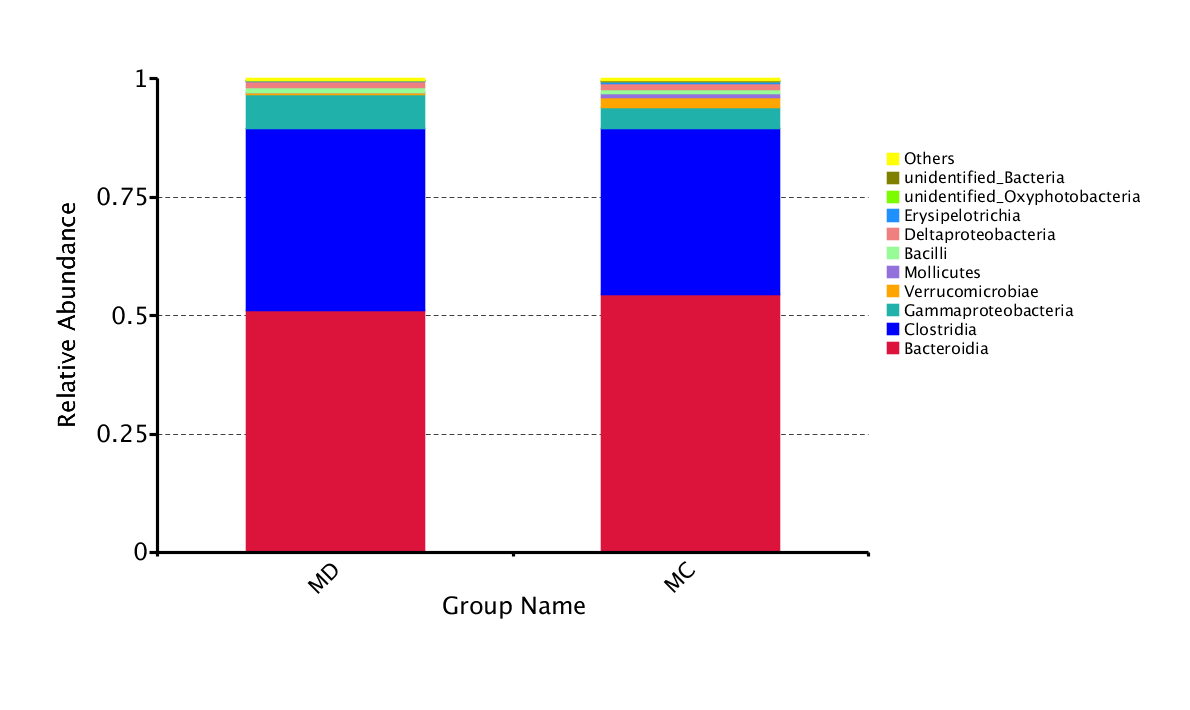

Supplement: Supplementary file 1 [file Data_Sheet_1.zip › P101SC18090073-01-B1-3-4_result/02.OTUanalysis/top10_group/class/c10.group.relative.dis.png]

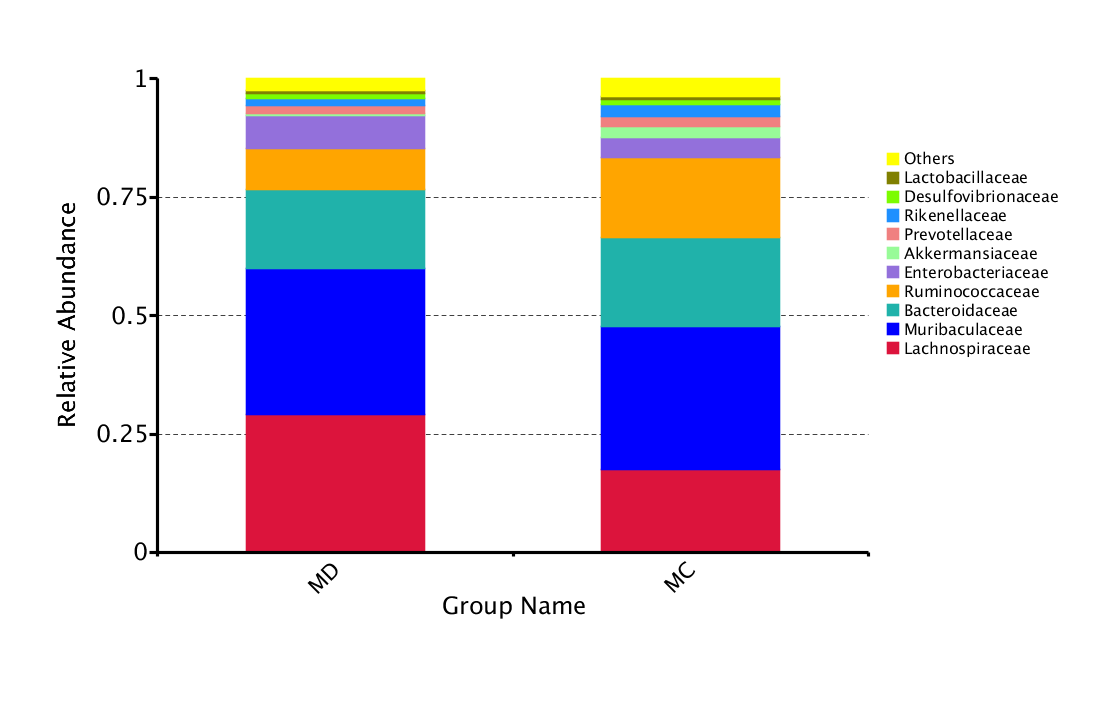

Supplement: Supplementary file 1 [file Data_Sheet_1.zip › P101SC18090073-01-B1-3-4_result/02.OTUanalysis/top10_group/family/f10.group.relative.dis.png]

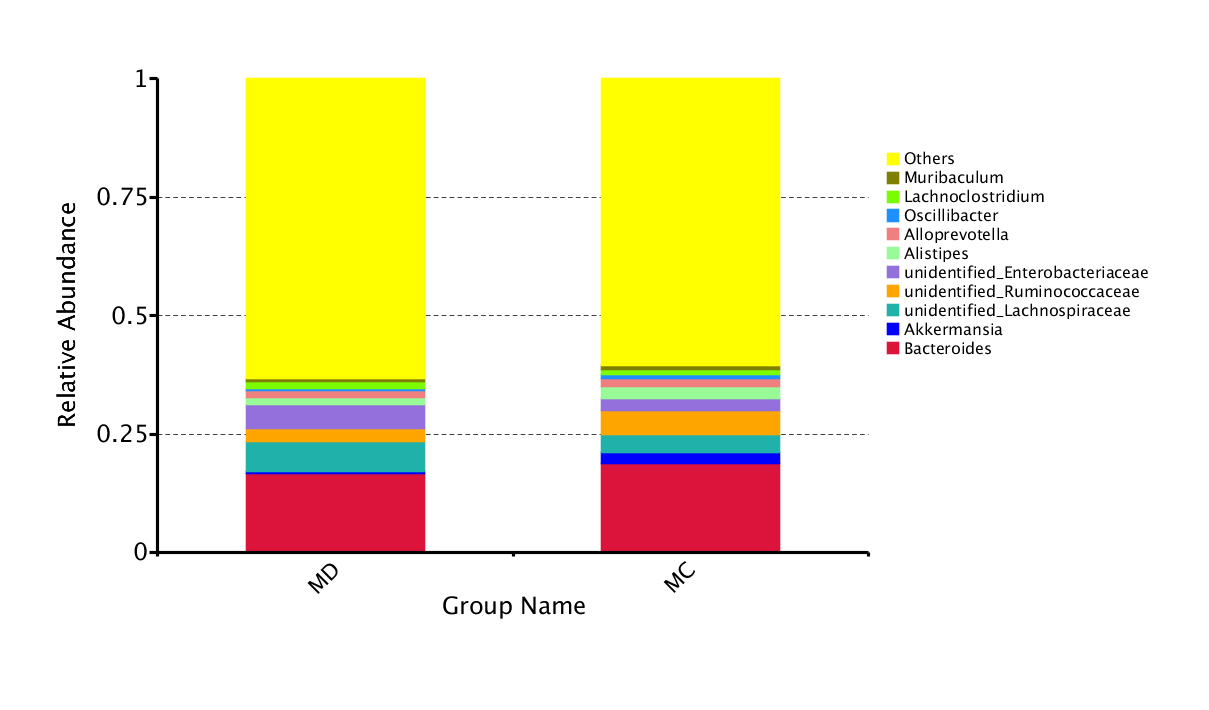

Supplement: Supplementary file 1 [file Data_Sheet_1.zip › P101SC18090073-01-B1-3-4_result/02.OTUanalysis/top10_group/genus/g10.group.relative.dis.png]

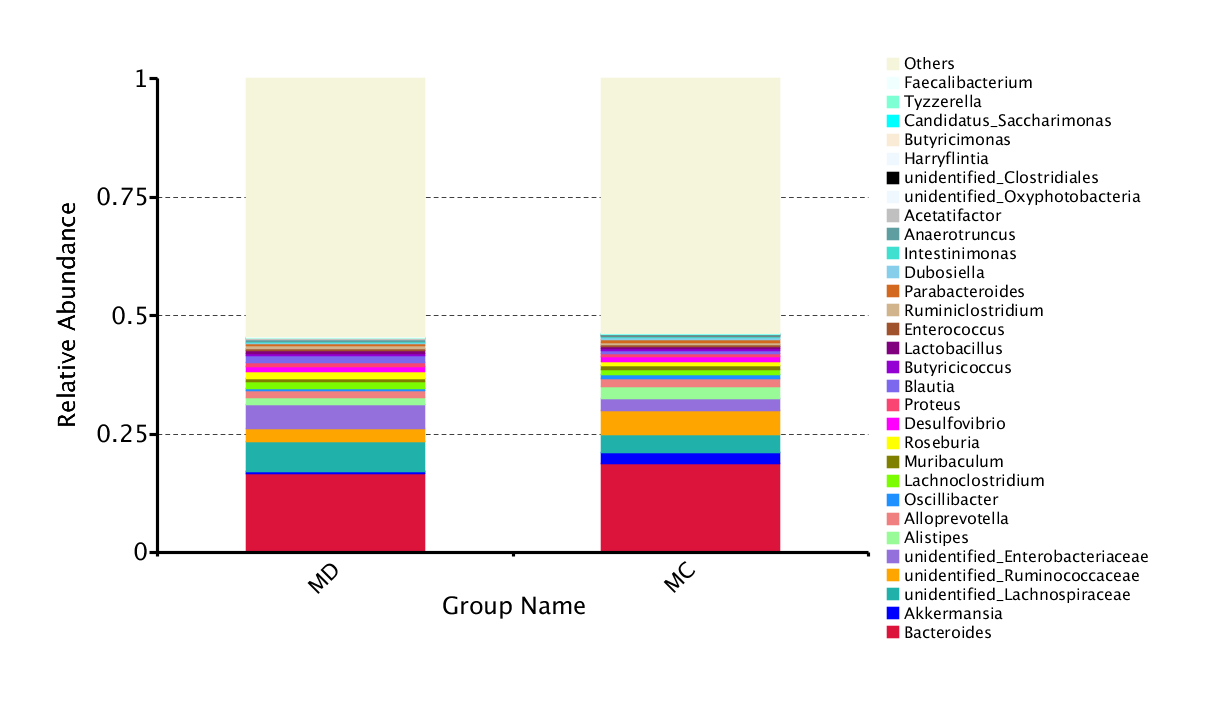

Supplement: Supplementary file 1 [file Data_Sheet_1.zip › P101SC18090073-01-B1-3-4_result/02.OTUanalysis/top10_group/genus/g30.group.relative.dis.png]

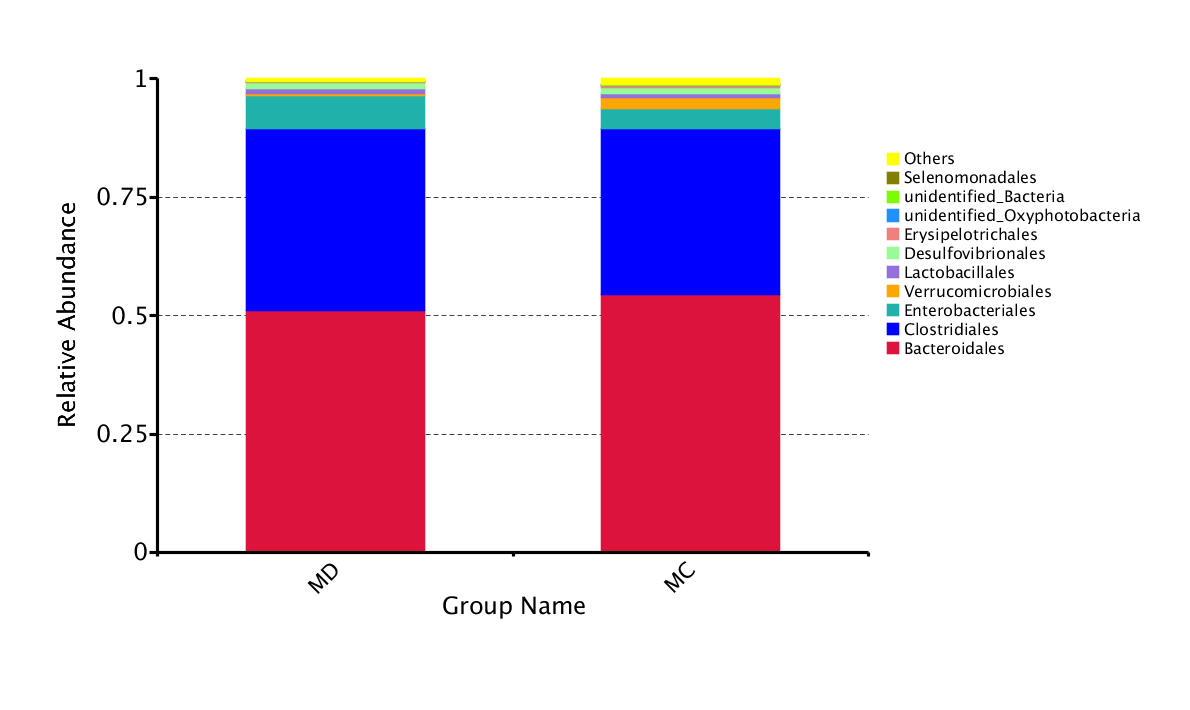

Supplement: Supplementary file 1 [file Data_Sheet_1.zip › P101SC18090073-01-B1-3-4_result/02.OTUanalysis/top10_group/order/o10.group.relative.dis.png]

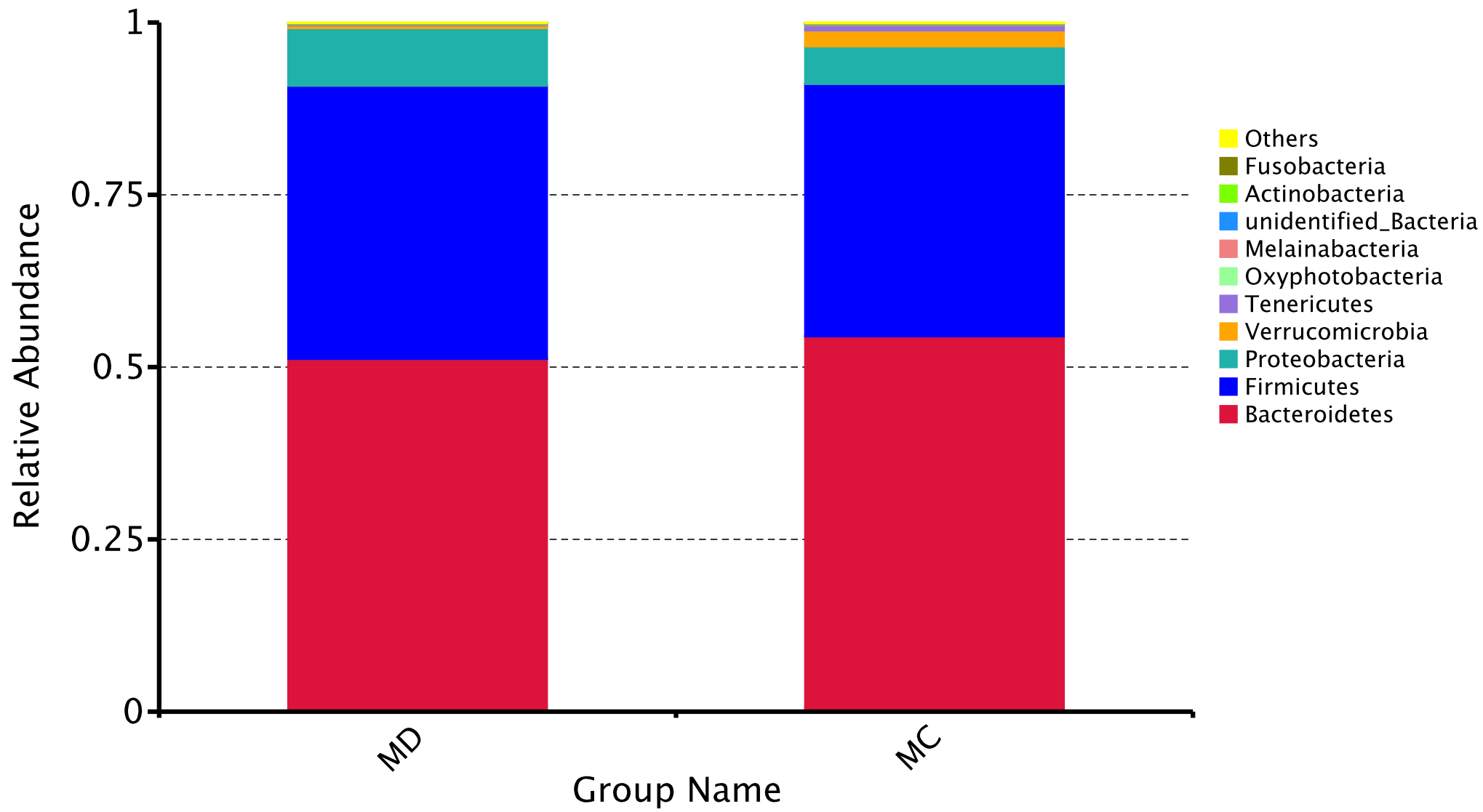

Supplement: Supplementary file 1 [file Data_Sheet_1.zip › P101SC18090073-01-B1-3-4_result/02.OTUanalysis/top10_group/phylum/p10.group.relative.dis.pdf]

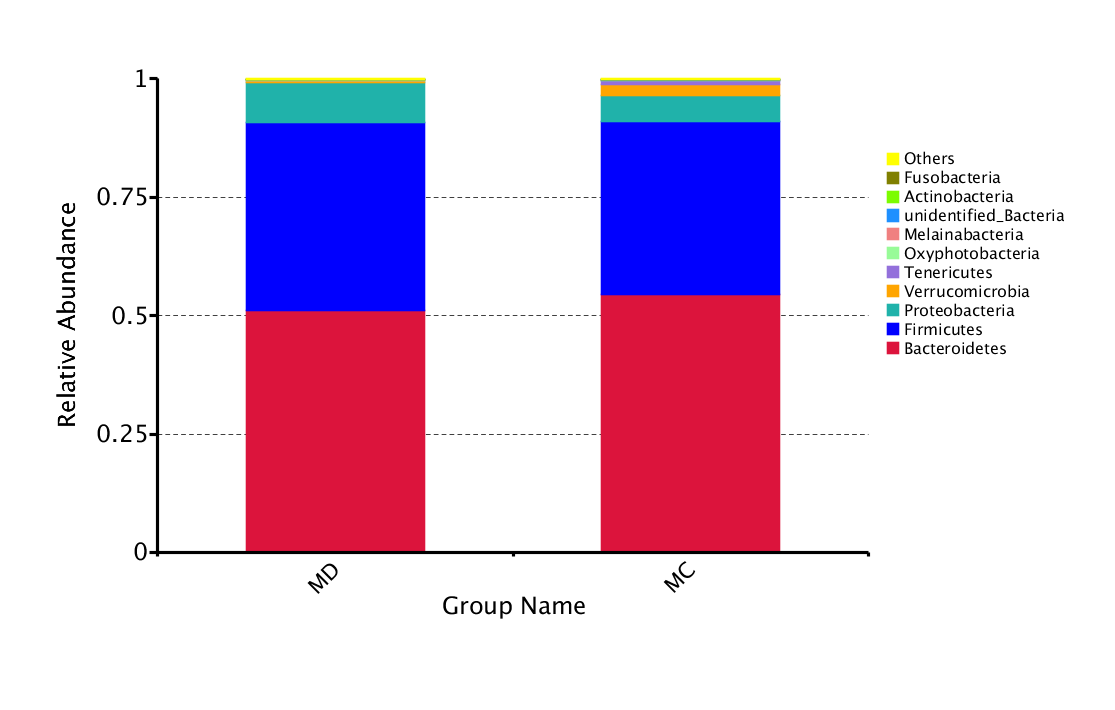

Supplement: Supplementary file 1 [file Data_Sheet_1.zip › P101SC18090073-01-B1-3-4_result/02.OTUanalysis/top10_group/phylum/p10.group.relative.dis.png]

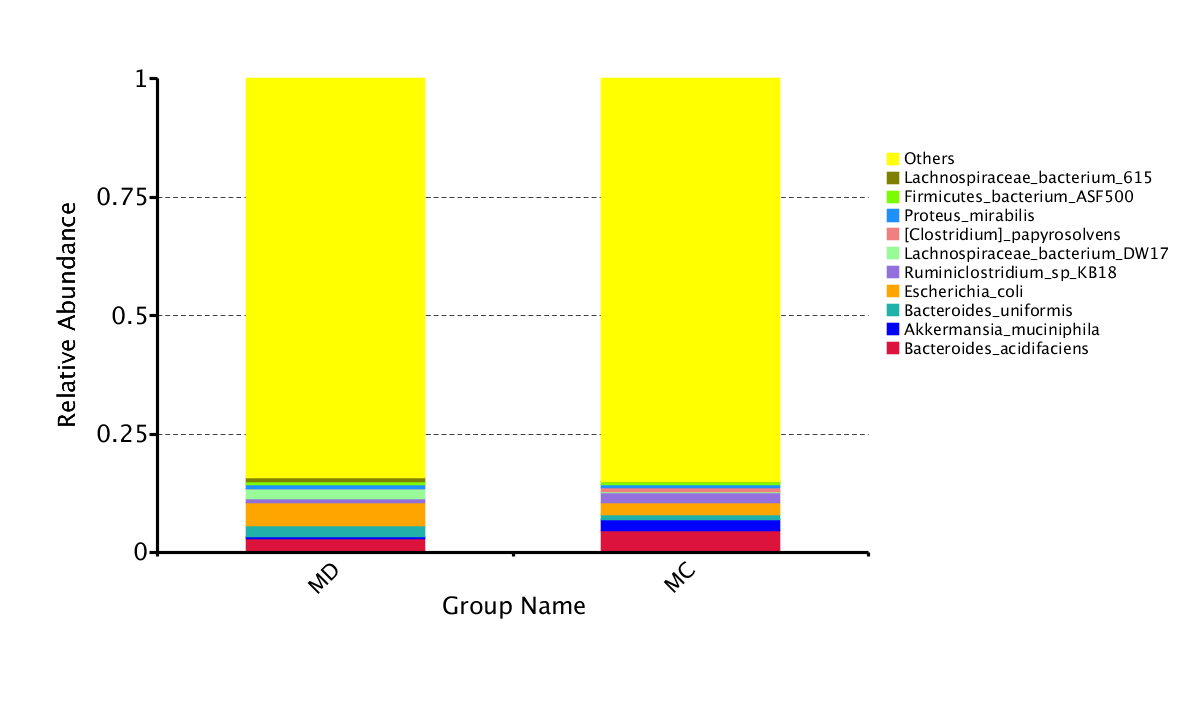

Supplement: Supplementary file 1 [file Data_Sheet_1.zip › P101SC18090073-01-B1-3-4_result/02.OTUanalysis/top10_group/species/s10.group.relative.dis.png]

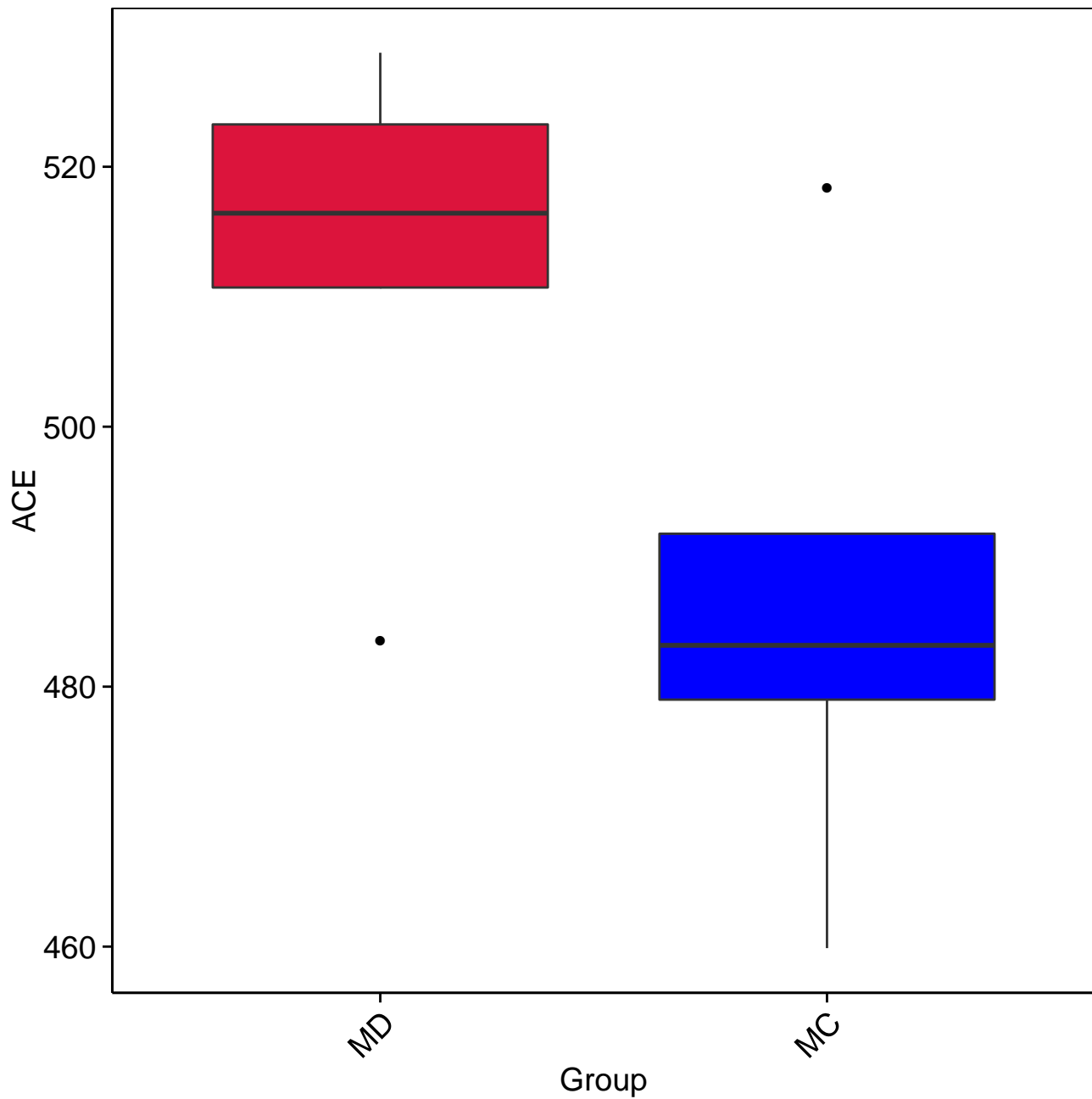

Supplement: Supplementary file 1 [file Data_Sheet_1.zip › P101SC18090073-01-B1-3-4_result/03.AlphaDiversity/Alpha_div/ACE/ACE.pdf]

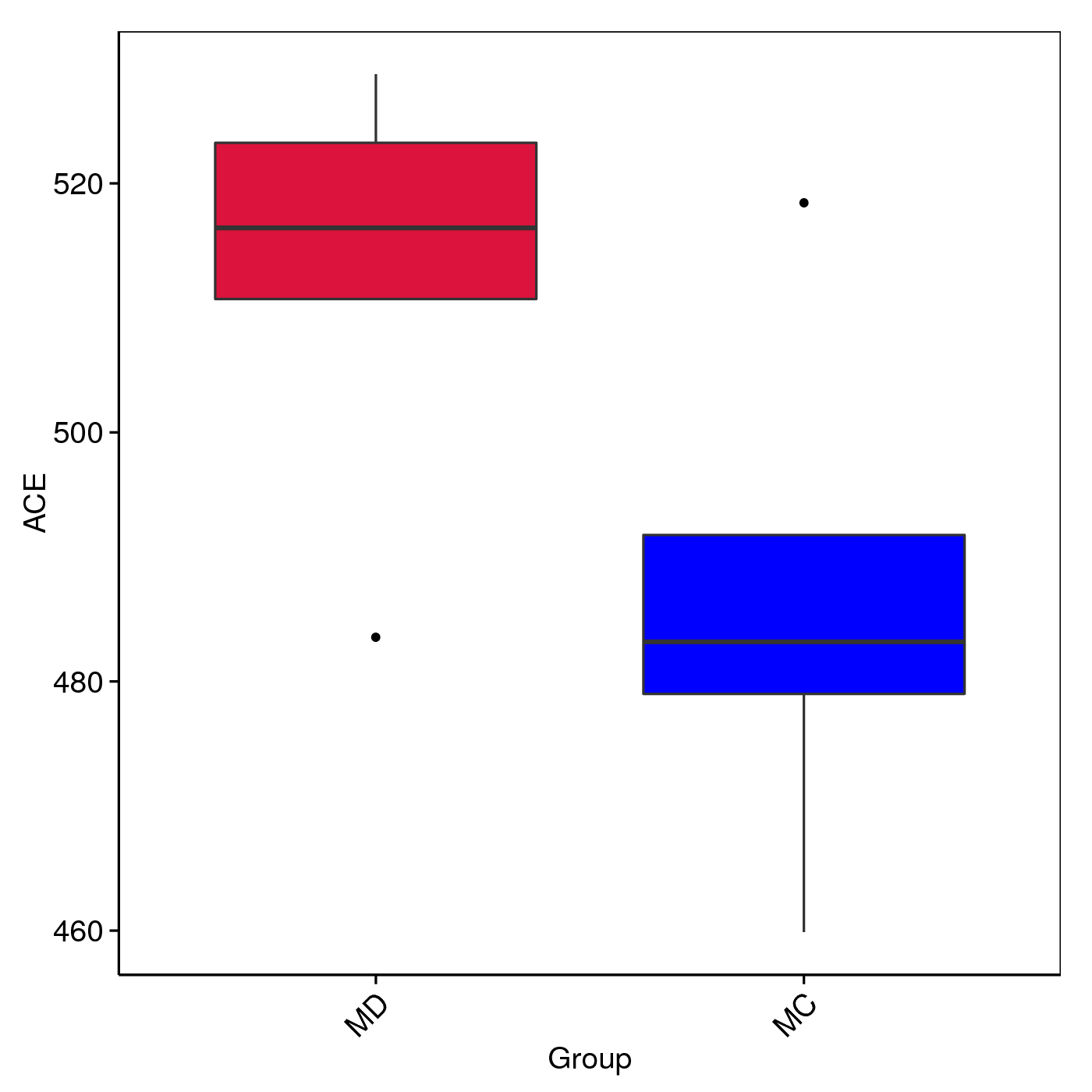

Supplement: Supplementary file 1 [file Data_Sheet_1.zip › P101SC18090073-01-B1-3-4_result/03.AlphaDiversity/Alpha_div/ACE/ACE.png]

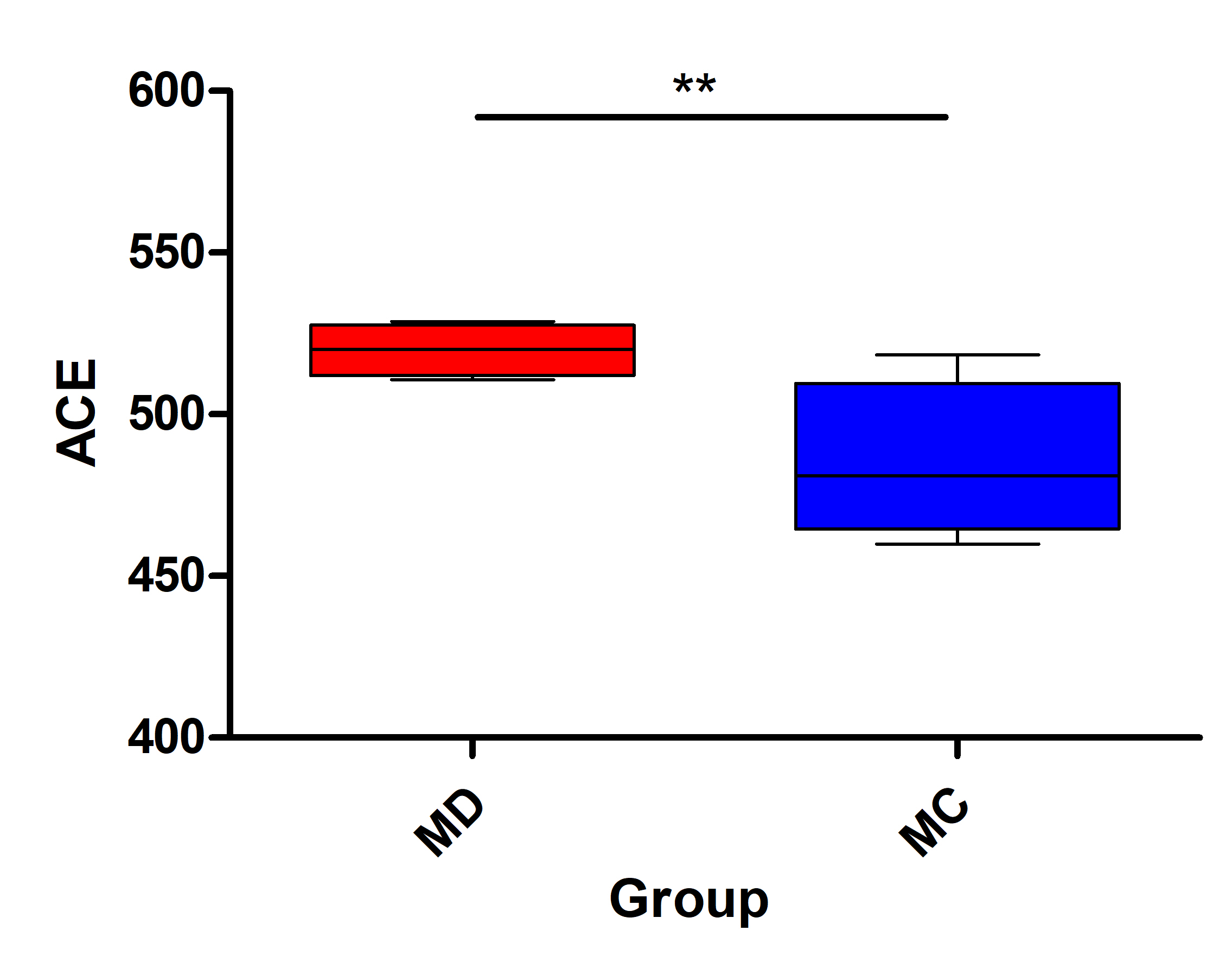

Supplement: Supplementary file 1 [file Data_Sheet_1.zip › P101SC18090073-01-B1-3-4_result/03.AlphaDiversity/Alpha_div/Alpha analysis/ACE.jpg]

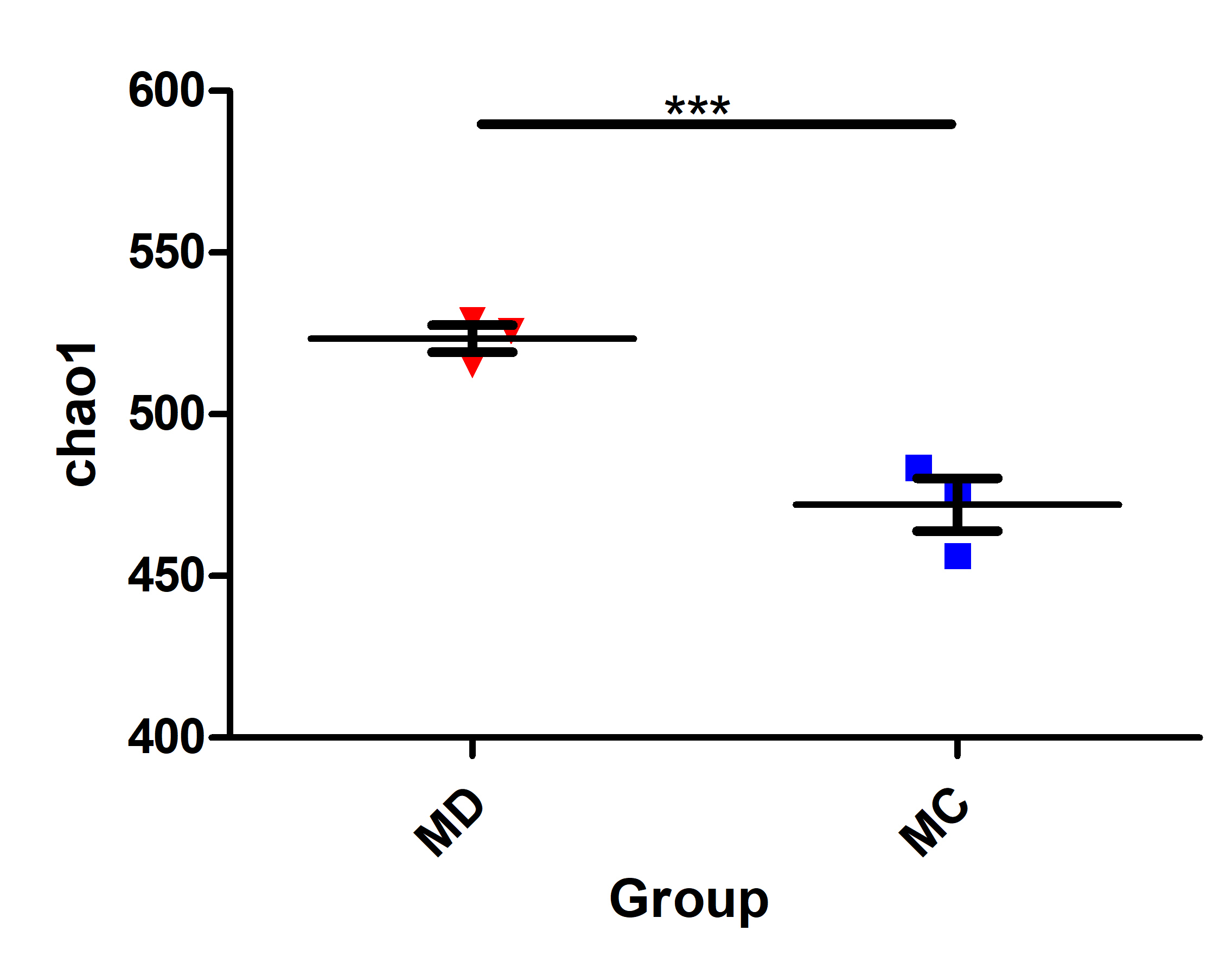

Supplement: Supplementary file 1 [file Data_Sheet_1.zip › P101SC18090073-01-B1-3-4_result/03.AlphaDiversity/Alpha_div/Alpha analysis/chao1-6.jpg]

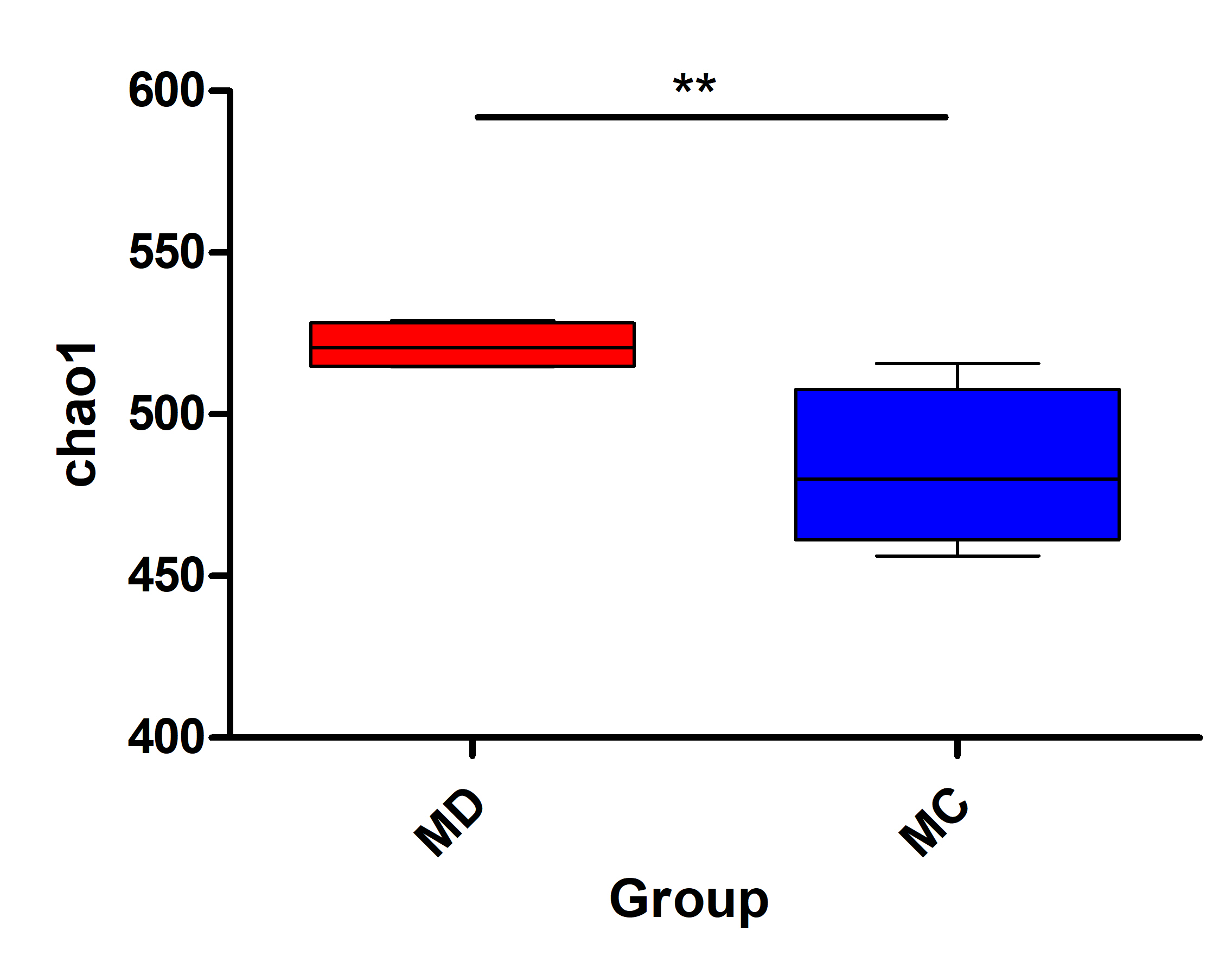

Supplement: Supplementary file 1 [file Data_Sheet_1.zip › P101SC18090073-01-B1-3-4_result/03.AlphaDiversity/Alpha_div/Alpha analysis/chao1.jpg]

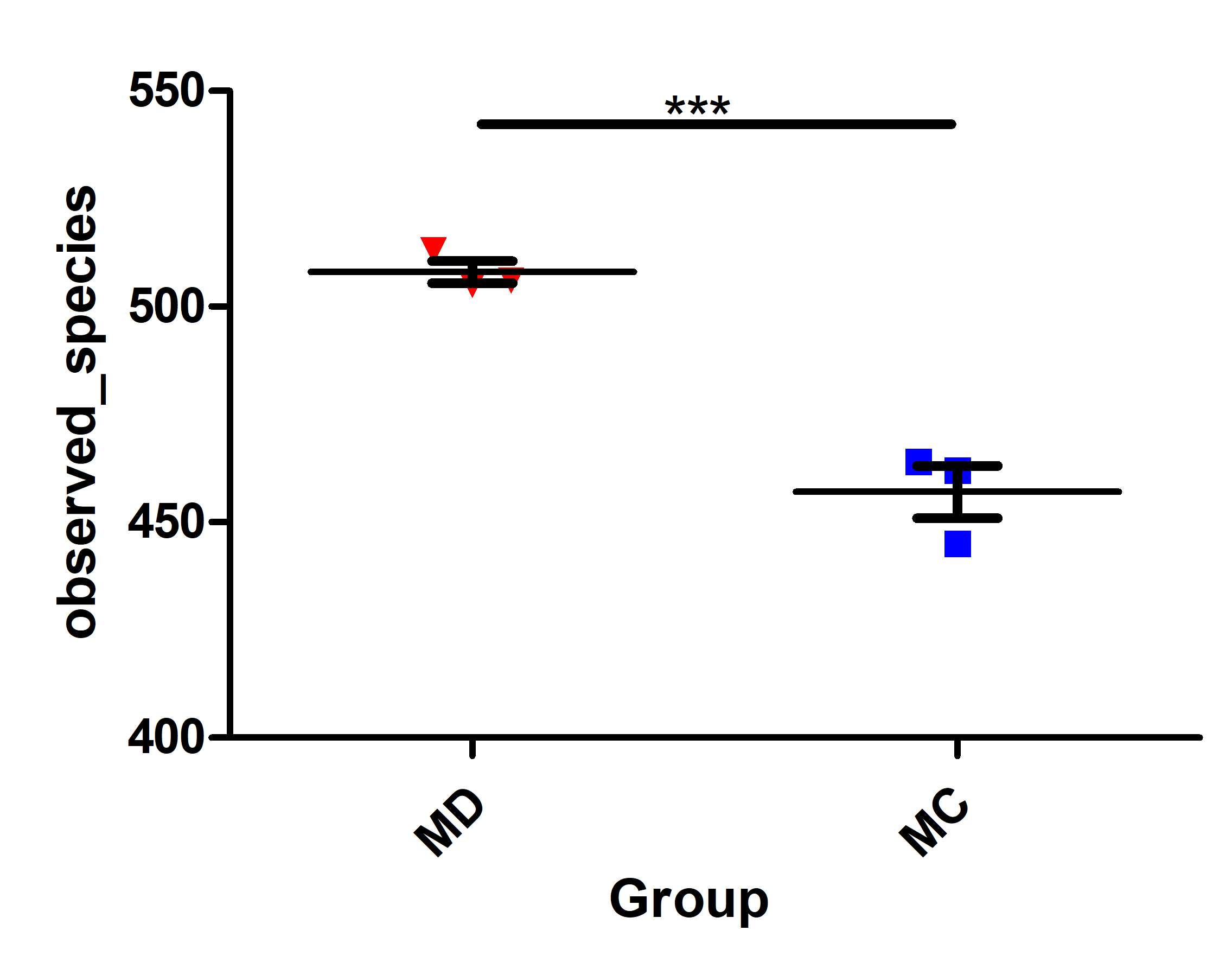

Supplement: Supplementary file 1 [file Data_Sheet_1.zip › P101SC18090073-01-B1-3-4_result/03.AlphaDiversity/Alpha_div/Alpha analysis/observe_species.jpg]

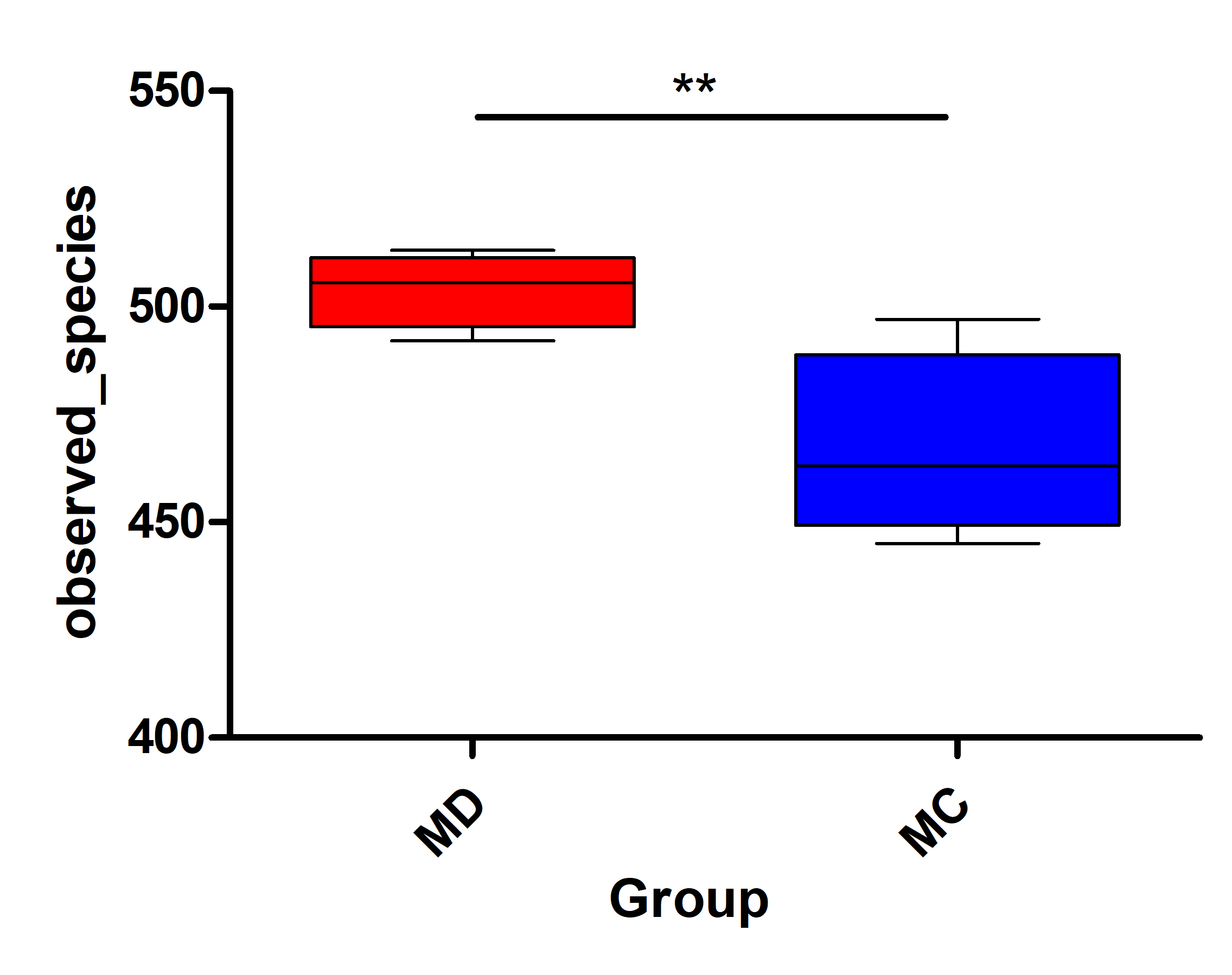

Supplement: Supplementary file 1 [file Data_Sheet_1.zip › P101SC18090073-01-B1-3-4_result/03.AlphaDiversity/Alpha_div/Alpha analysis/observed_species.jpg]

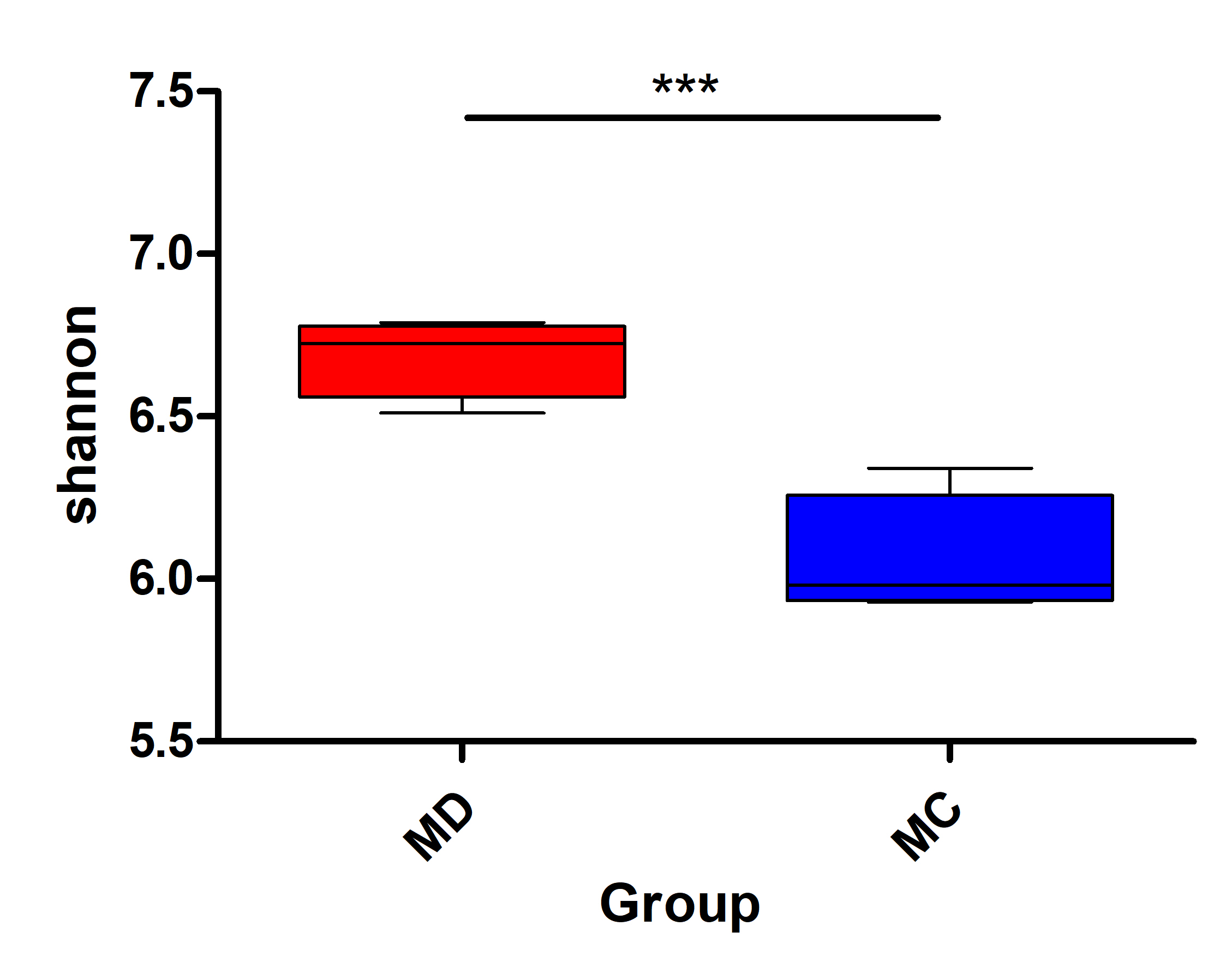

Supplement: Supplementary file 1 [file Data_Sheet_1.zip › P101SC18090073-01-B1-3-4_result/03.AlphaDiversity/Alpha_div/Alpha analysis/shannon.jpg]

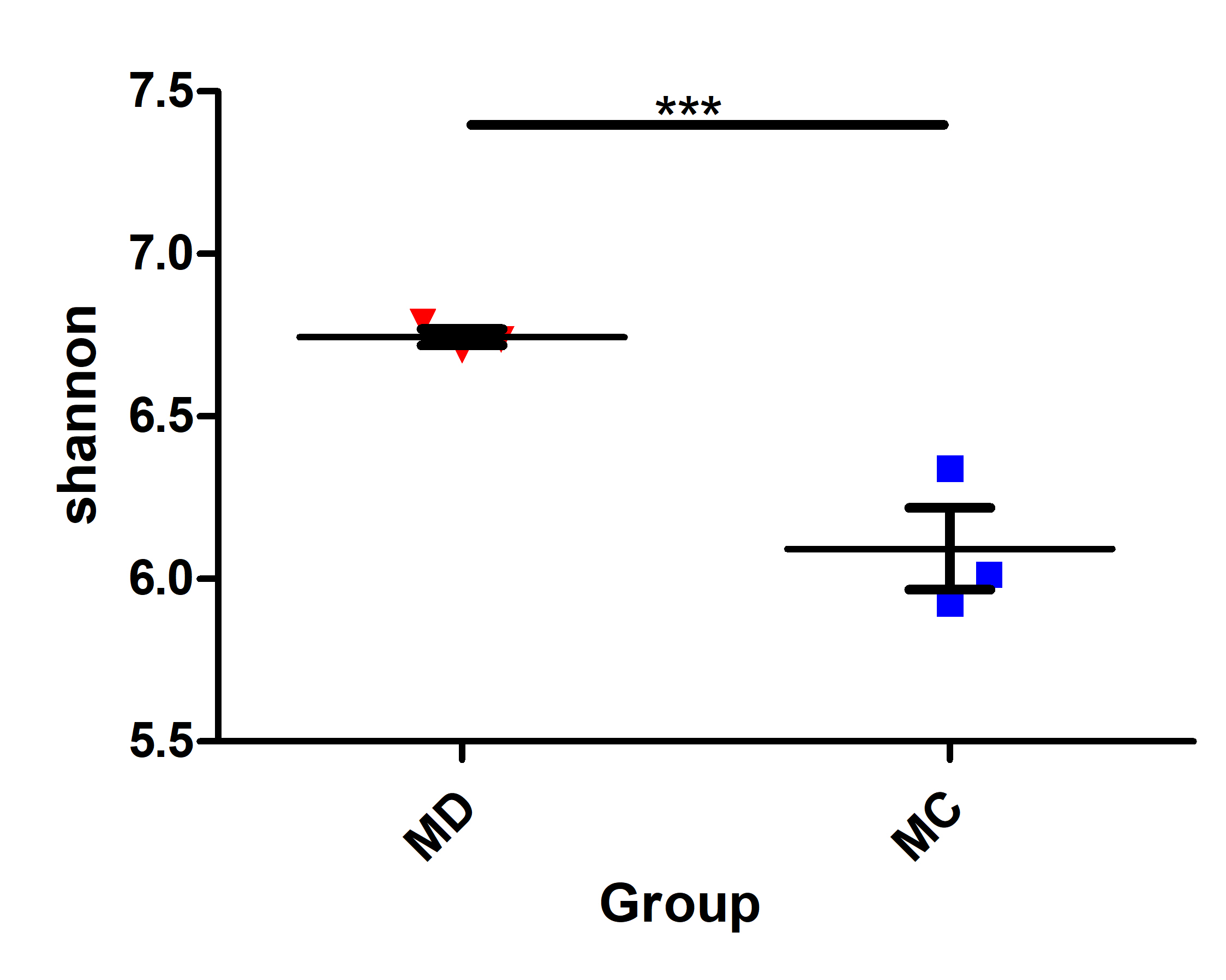

Supplement: Supplementary file 1 [file Data_Sheet_1.zip › P101SC18090073-01-B1-3-4_result/03.AlphaDiversity/Alpha_div/Alpha analysis/shannon6.jpg]

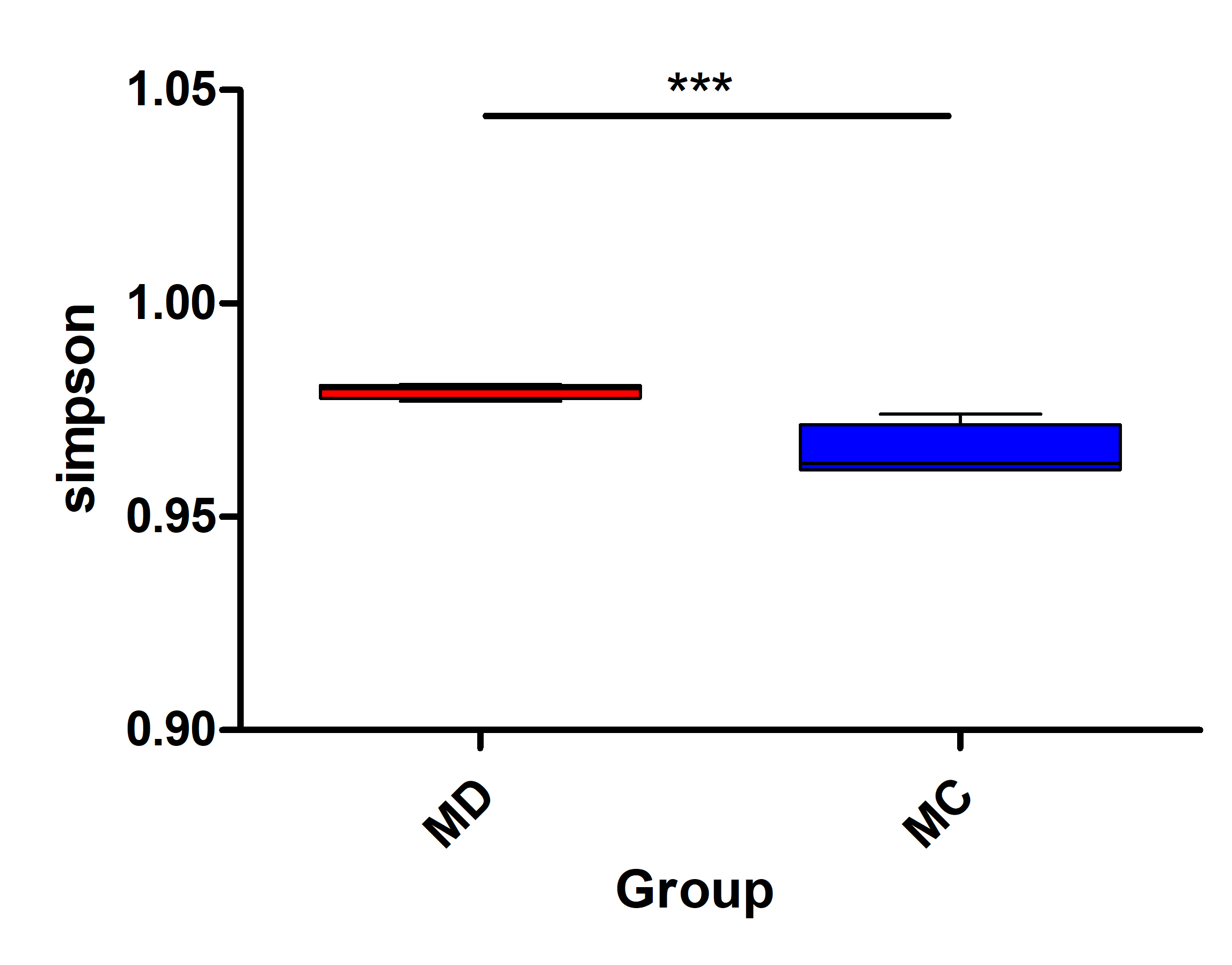

Supplement: Supplementary file 1 [file Data_Sheet_1.zip › P101SC18090073-01-B1-3-4_result/03.AlphaDiversity/Alpha_div/Alpha analysis/simpson.jpg]

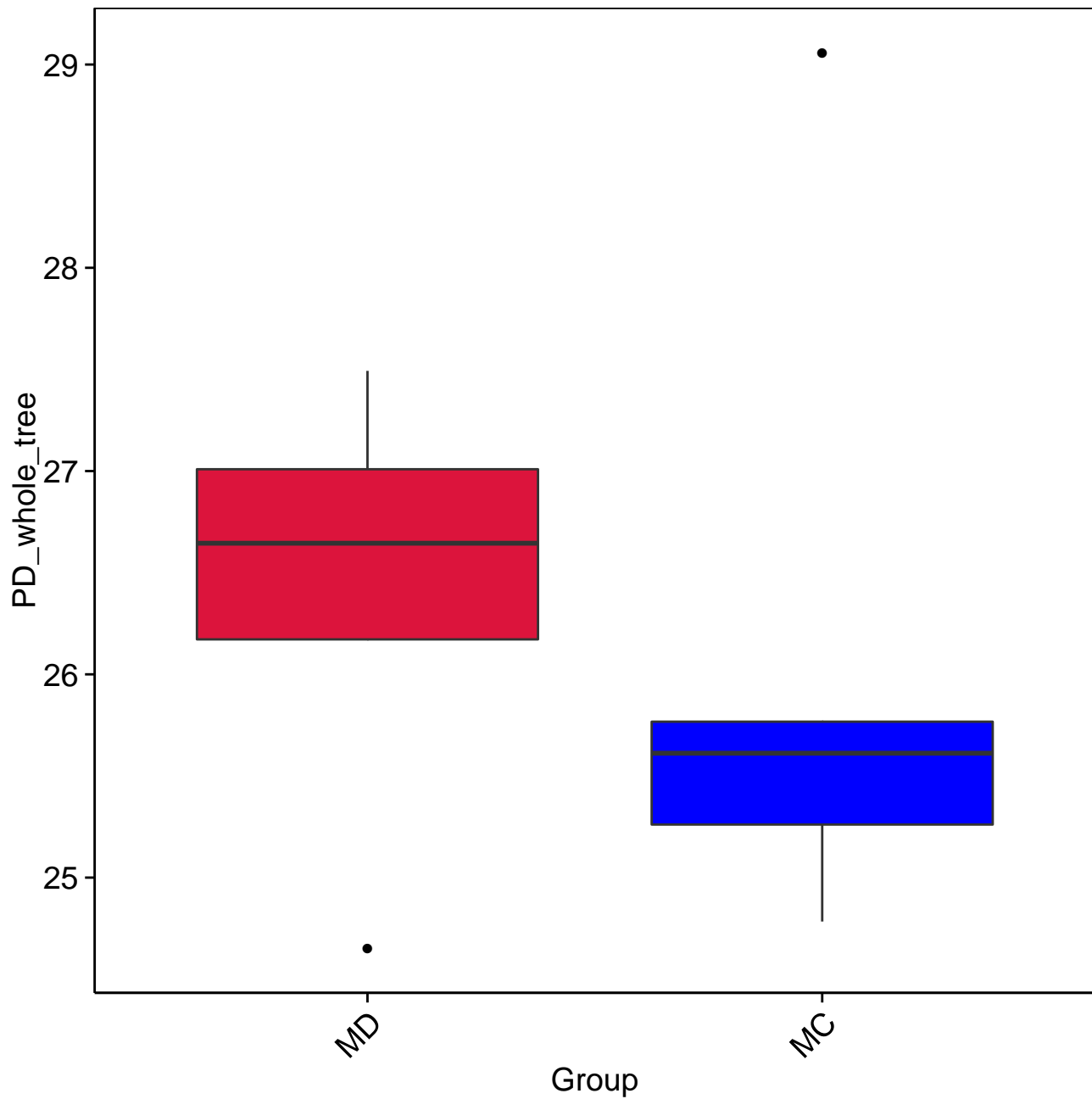

Supplement: Supplementary file 1 [file Data_Sheet_1.zip › P101SC18090073-01-B1-3-4_result/03.AlphaDiversity/Alpha_div/PD_whole_tree/PD_whole_tree.pdf]

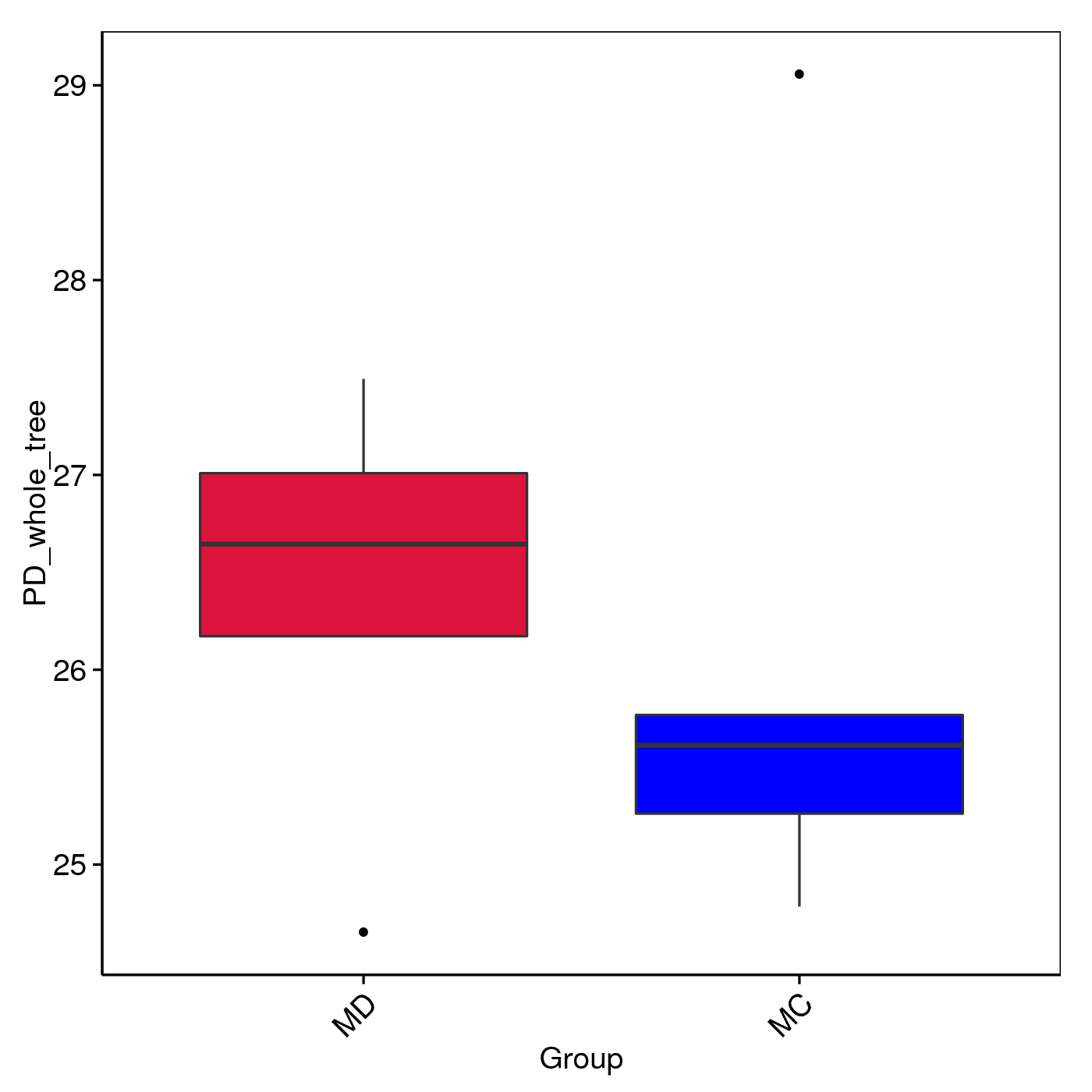

Supplement: Supplementary file 1 [file Data_Sheet_1.zip › P101SC18090073-01-B1-3-4_result/03.AlphaDiversity/Alpha_div/PD_whole_tree/PD_whole_tree.png]

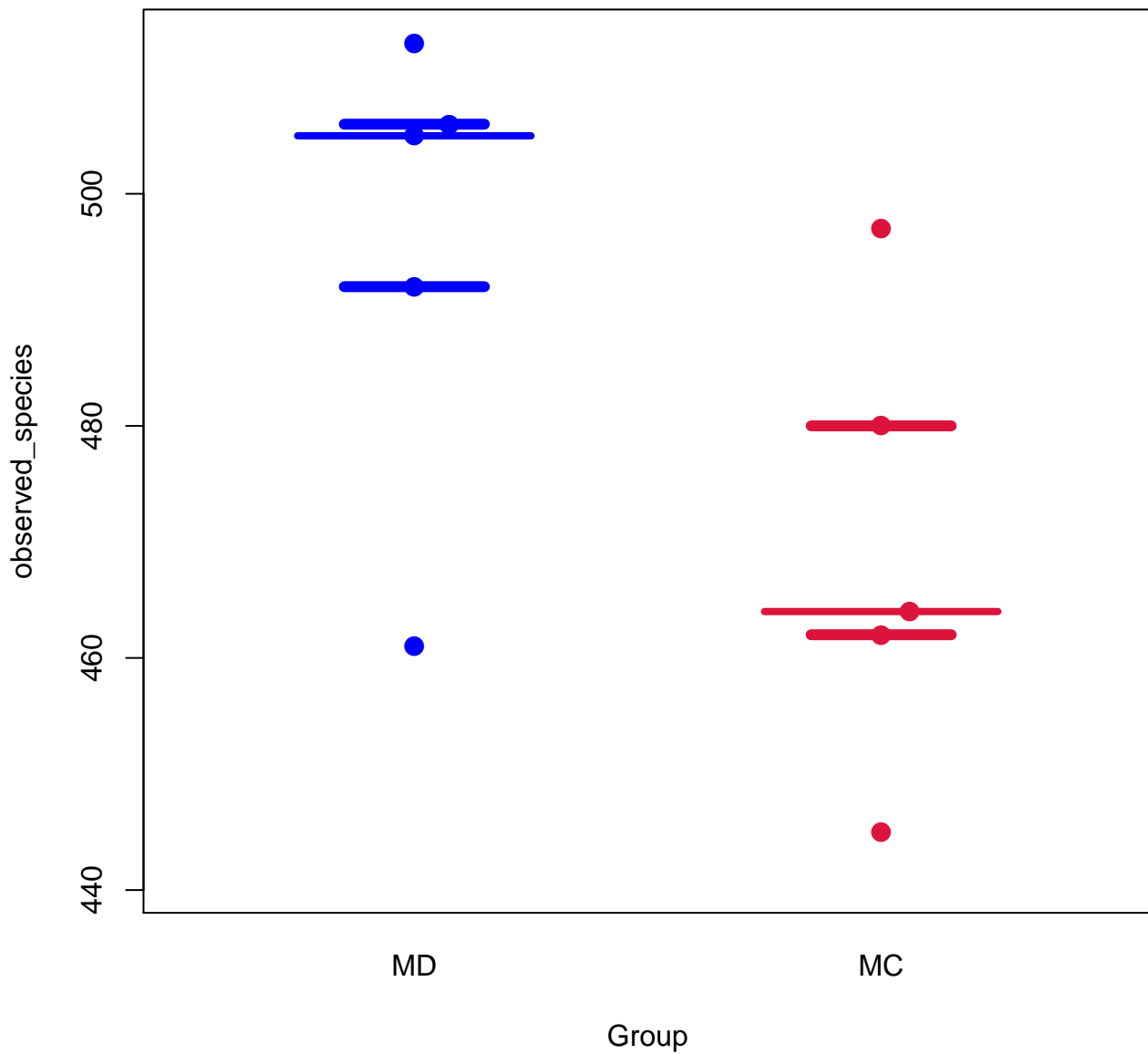

Supplement: Supplementary file 1 [file Data_Sheet_1.zip › P101SC18090073-01-B1-3-4_result/03.AlphaDiversity/Alpha_div/beewarm/observed_species_beeswarm.pdf]

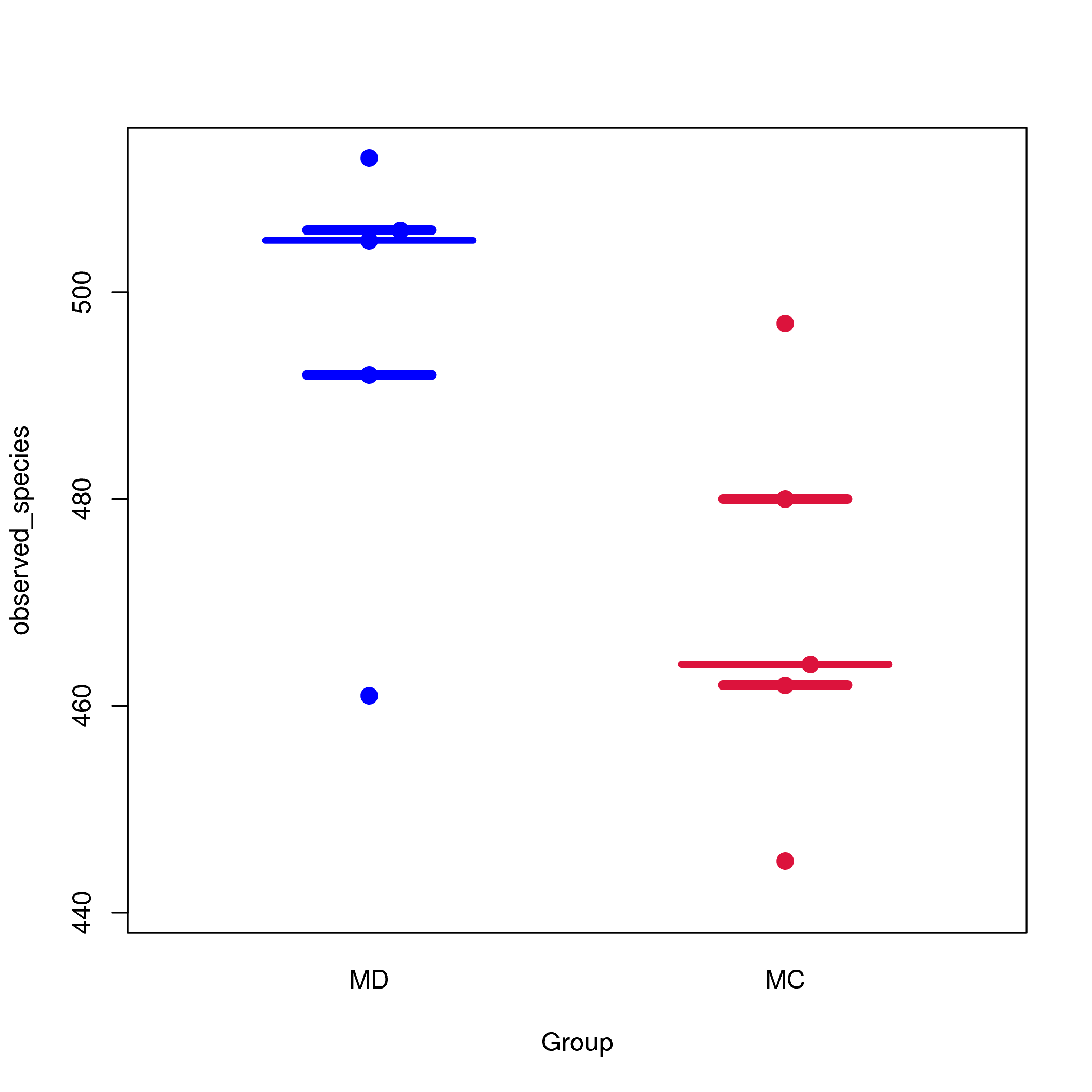

Supplement: Supplementary file 1 [file Data_Sheet_1.zip › P101SC18090073-01-B1-3-4_result/03.AlphaDiversity/Alpha_div/beewarm/observed_species_beeswarm.png]

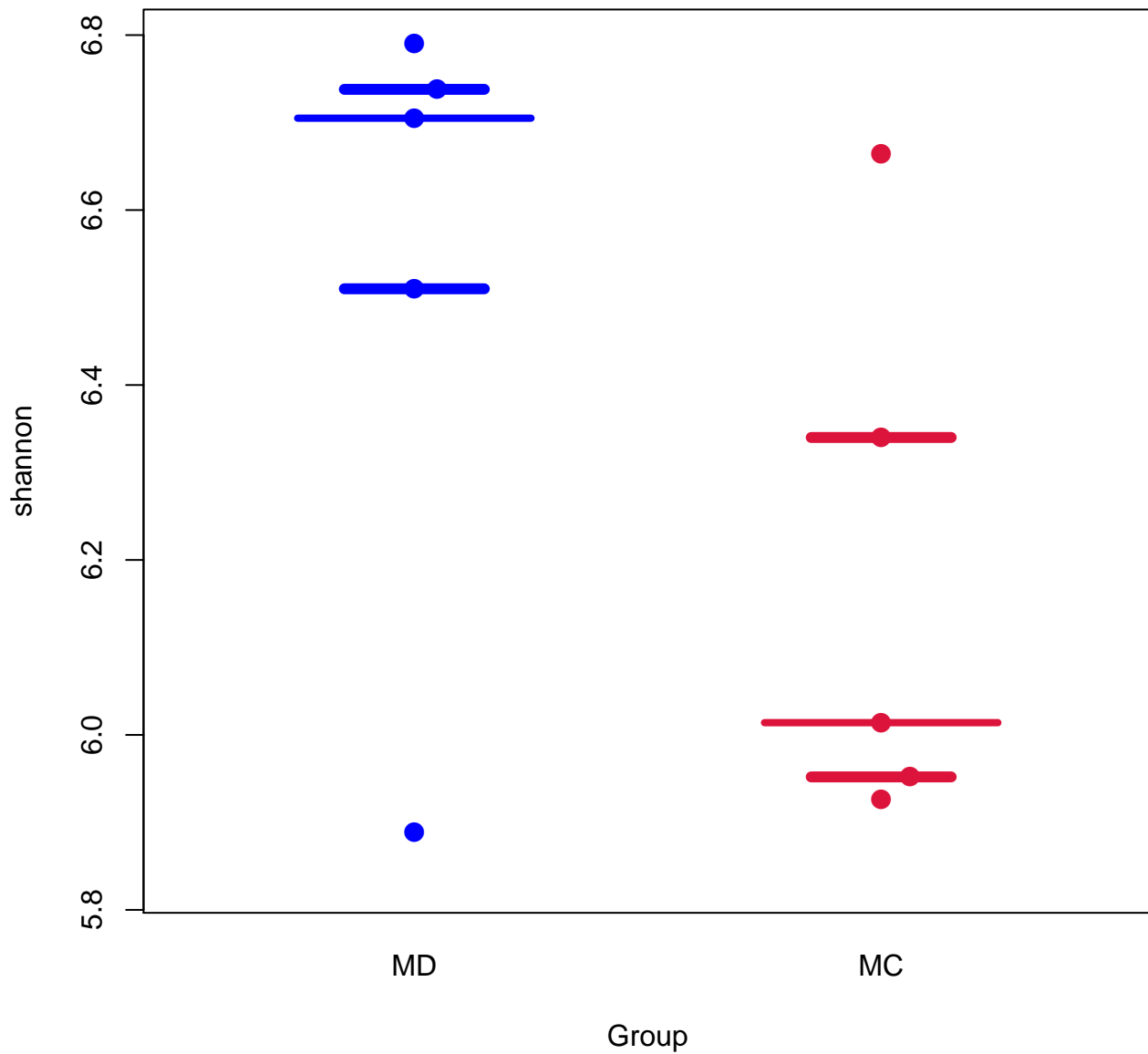

Supplement: Supplementary file 1 [file Data_Sheet_1.zip › P101SC18090073-01-B1-3-4_result/03.AlphaDiversity/Alpha_div/beewarm/shannon_beeswarm.pdf]

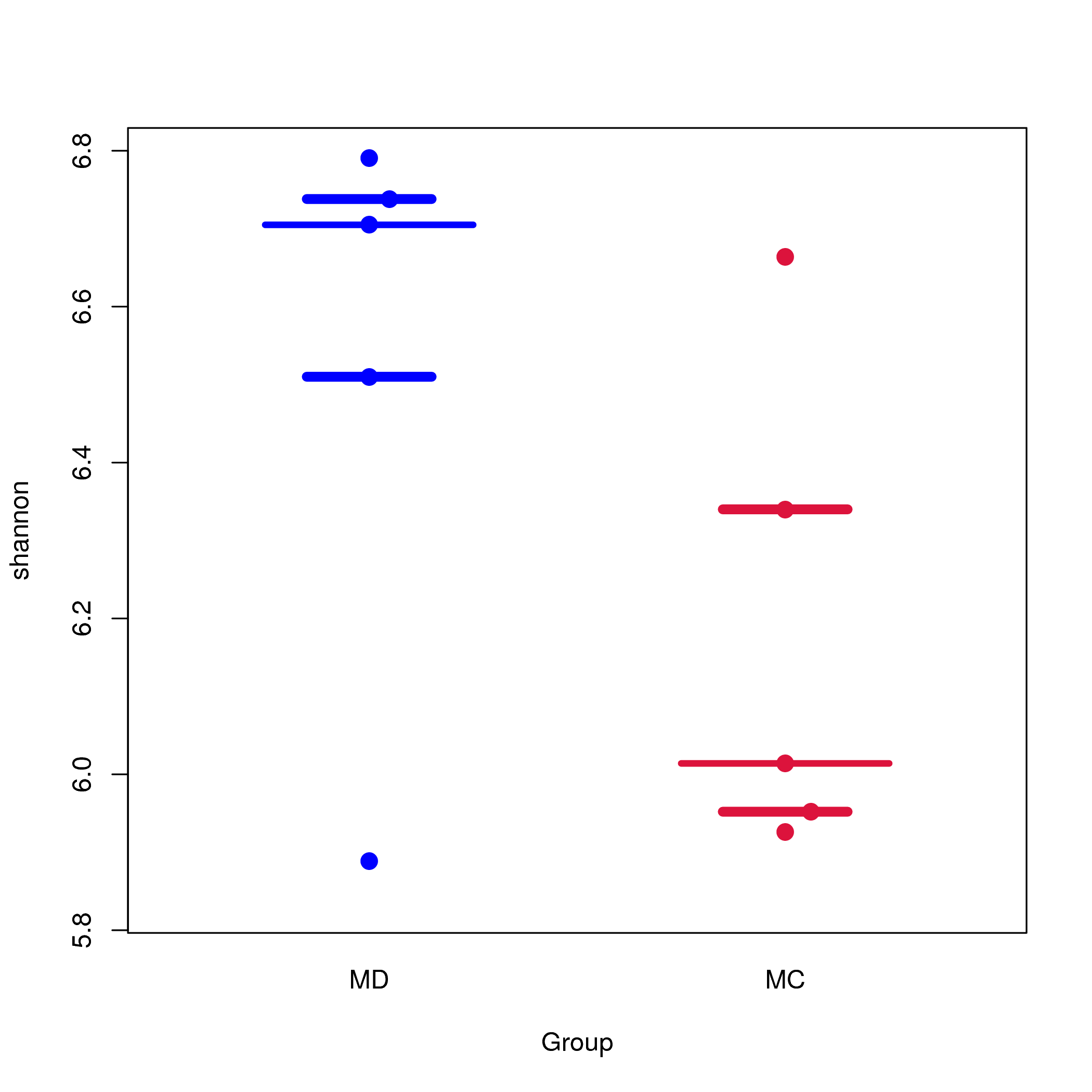

Supplement: Supplementary file 1 [file Data_Sheet_1.zip › P101SC18090073-01-B1-3-4_result/03.AlphaDiversity/Alpha_div/beewarm/shannon_beeswarm.png]

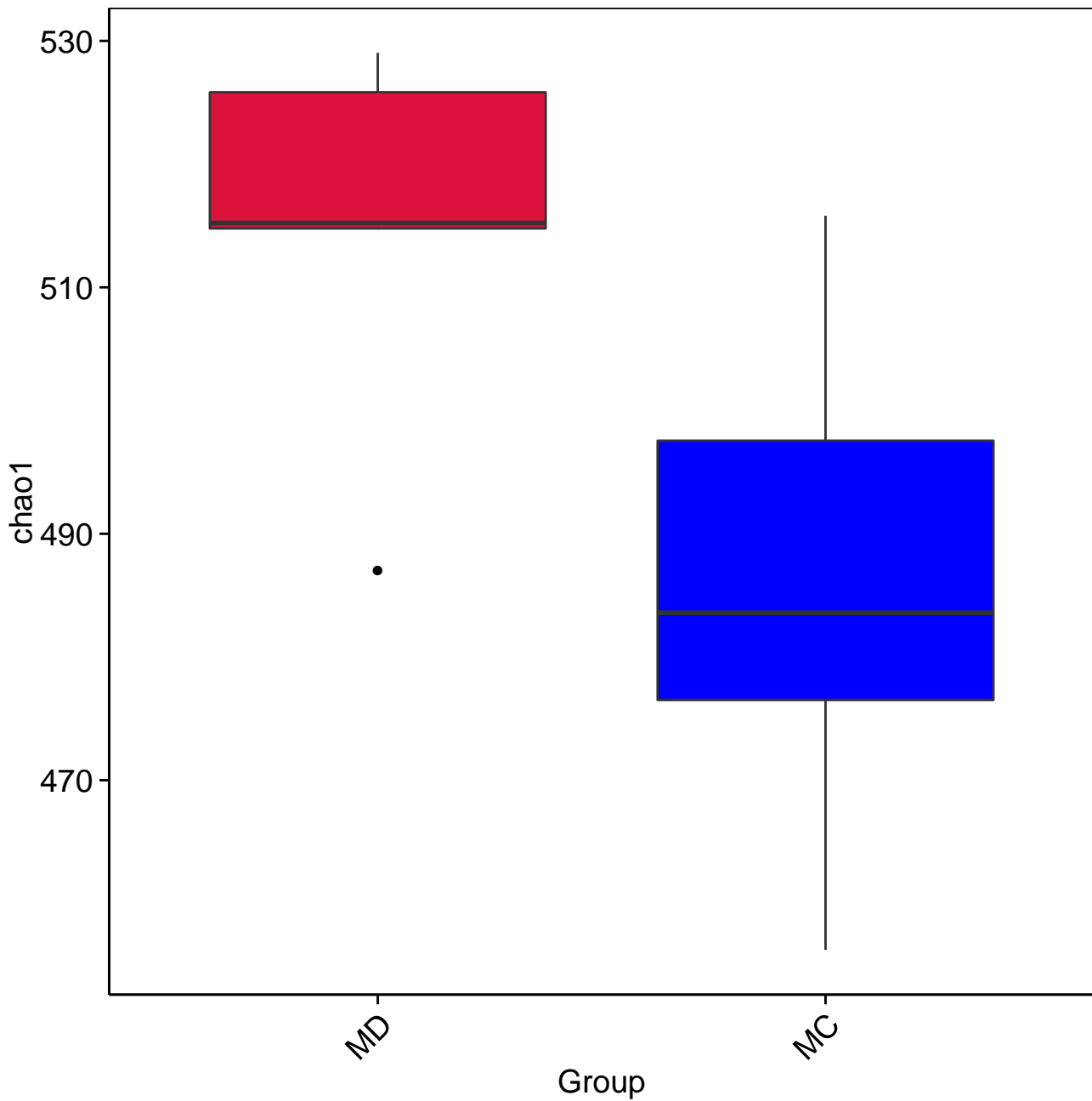

Supplement: Supplementary file 1 [file Data_Sheet_1.zip › P101SC18090073-01-B1-3-4_result/03.AlphaDiversity/Alpha_div/chao1/chao1.pdf]

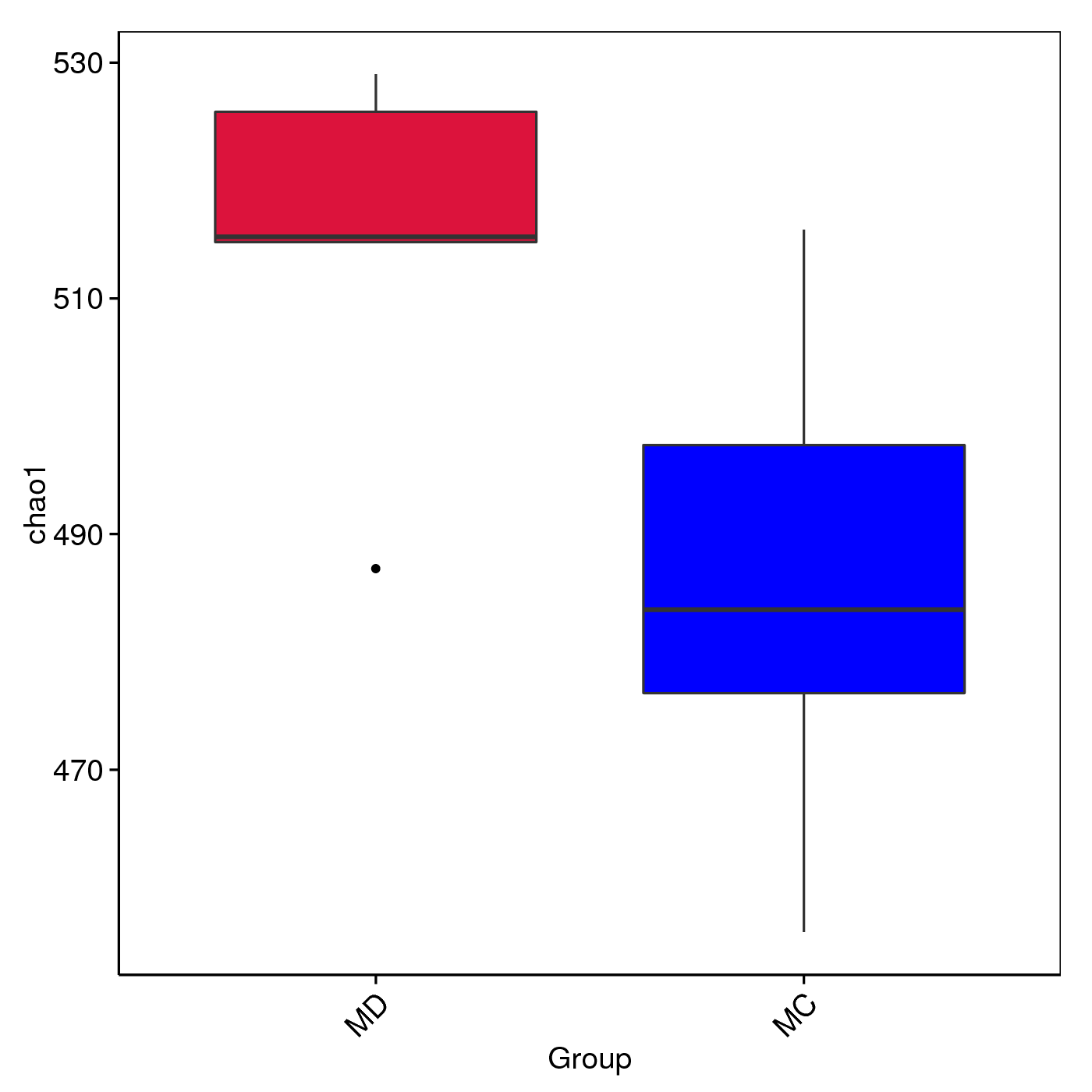

Supplement: Supplementary file 1 [file Data_Sheet_1.zip › P101SC18090073-01-B1-3-4_result/03.AlphaDiversity/Alpha_div/chao1/chao1.png]

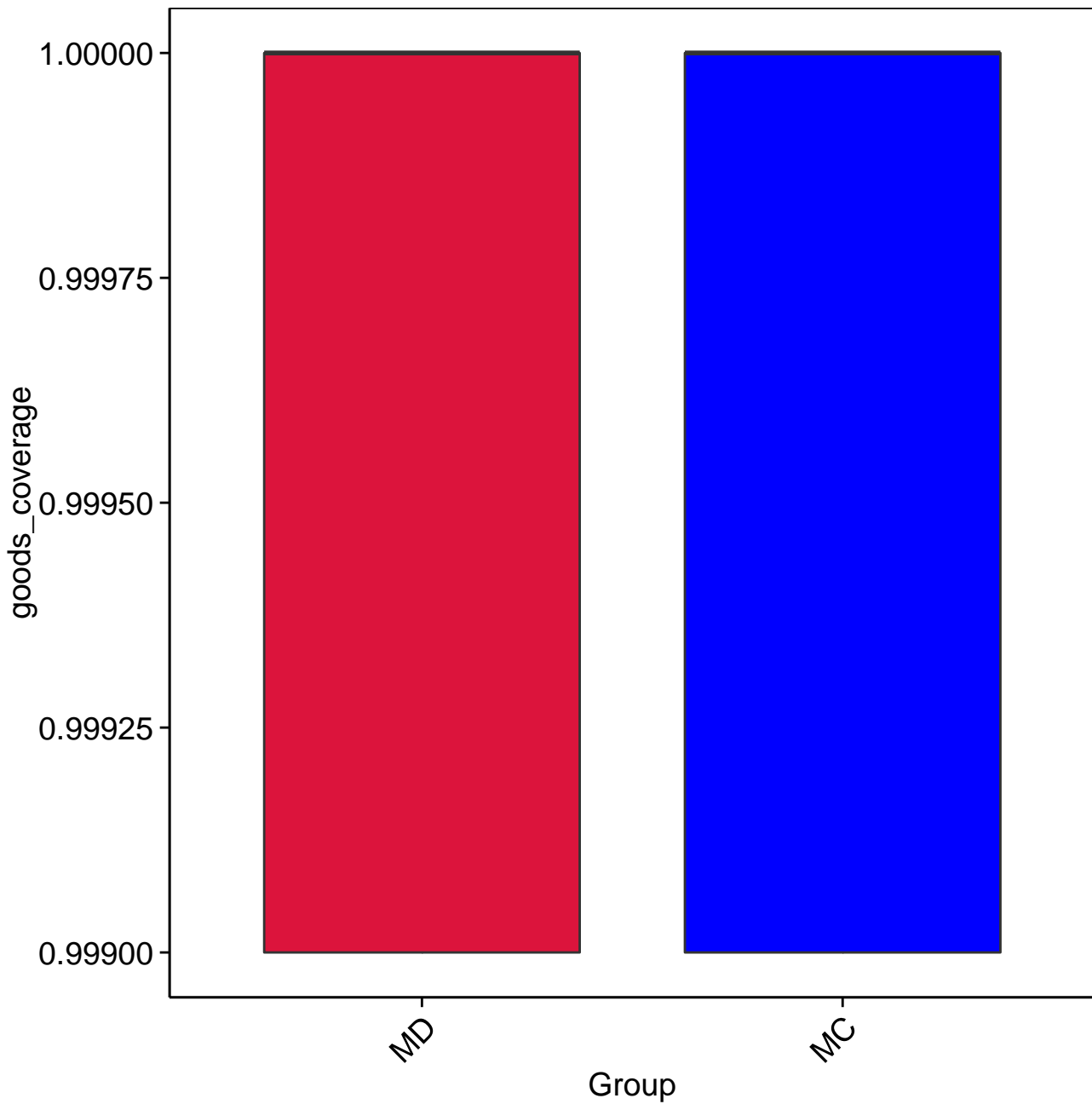

Supplement: Supplementary file 1 [file Data_Sheet_1.zip › P101SC18090073-01-B1-3-4_result/03.AlphaDiversity/Alpha_div/goods_coverage/goods_coverage.pdf]

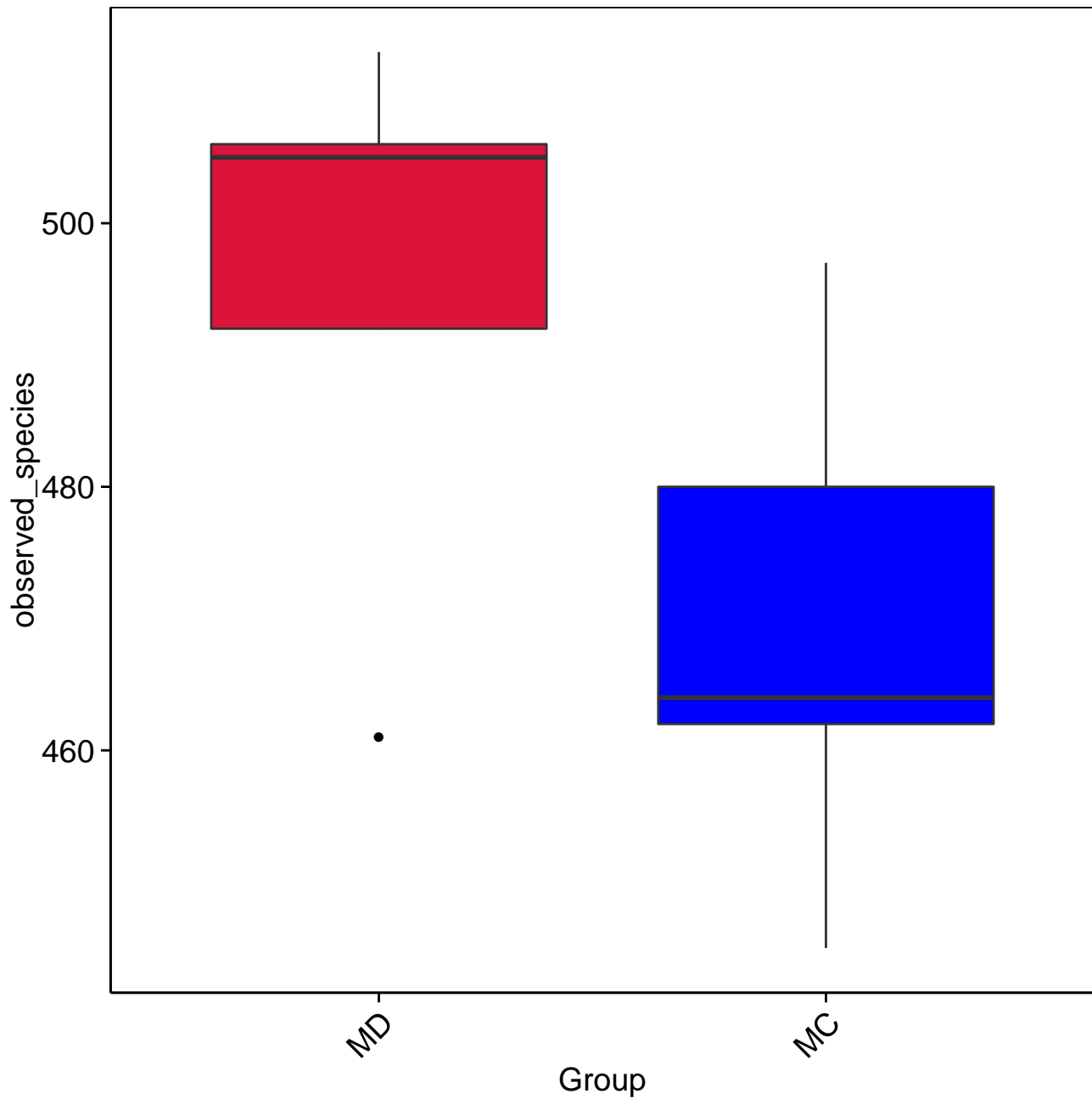

Supplement: Supplementary file 1 [file Data_Sheet_1.zip › P101SC18090073-01-B1-3-4_result/03.AlphaDiversity/Alpha_div/observed_species/observed_species.pdf]

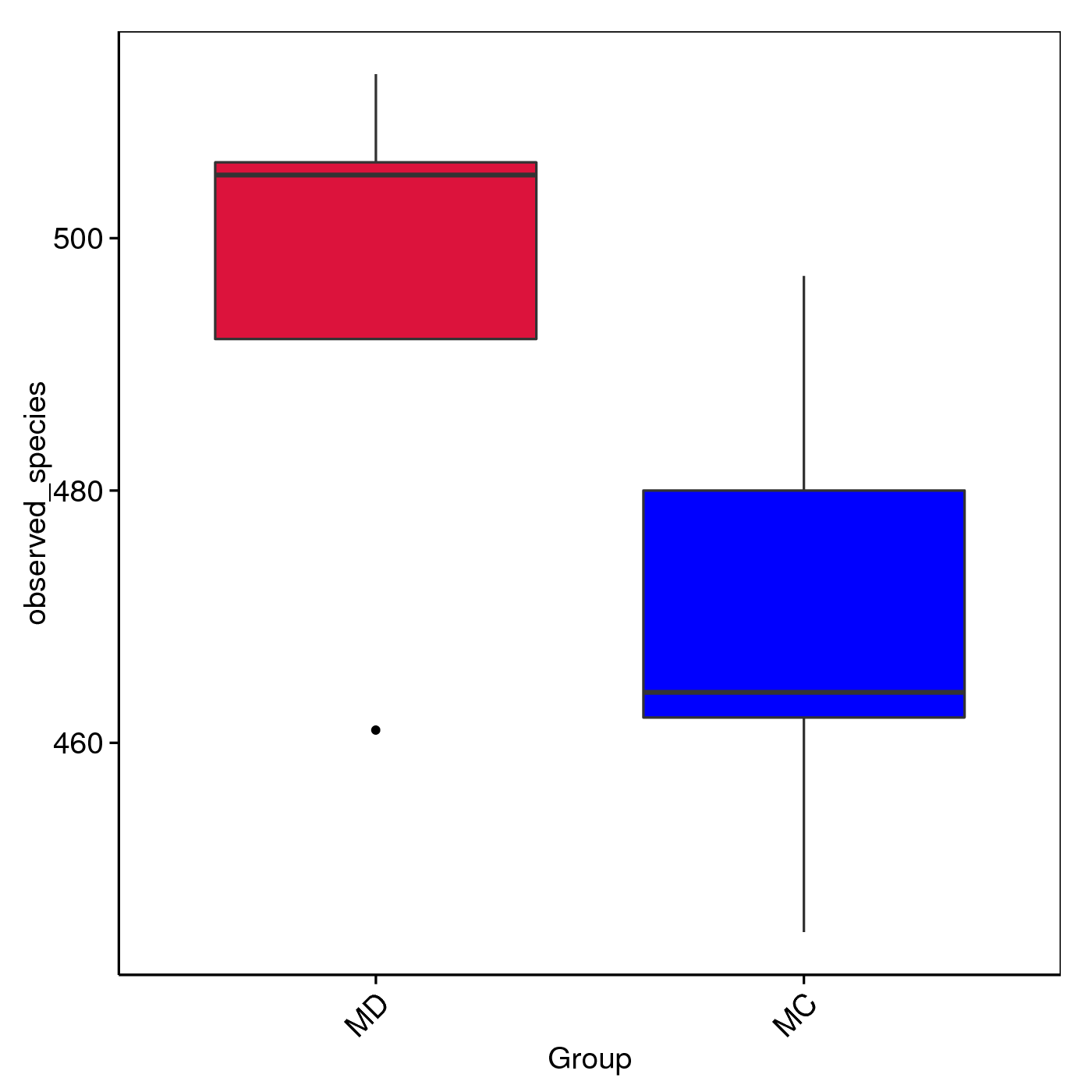

Supplement: Supplementary file 1 [file Data_Sheet_1.zip › P101SC18090073-01-B1-3-4_result/03.AlphaDiversity/Alpha_div/observed_species/observed_species.png]

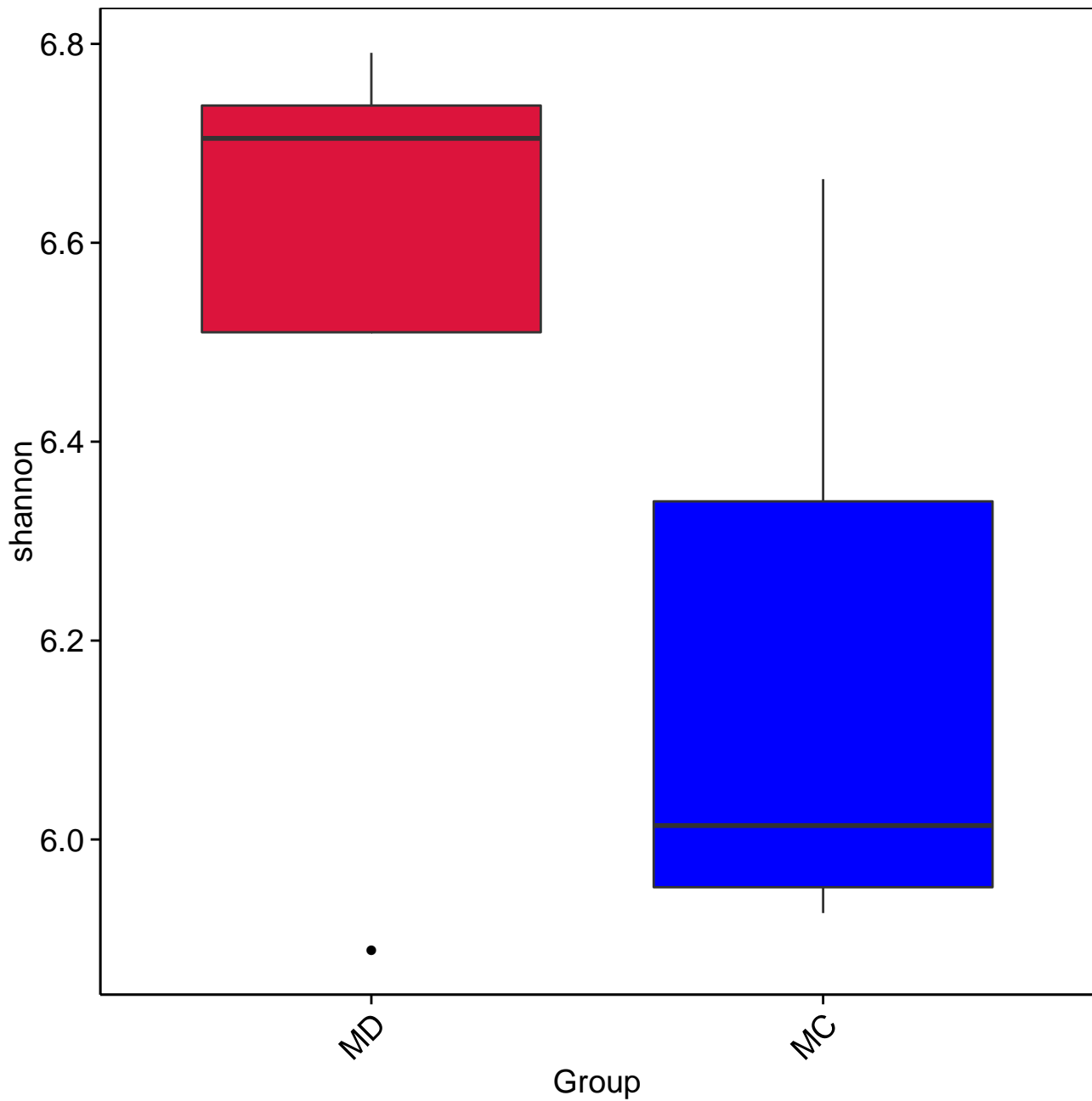

Supplement: Supplementary file 1 [file Data_Sheet_1.zip › P101SC18090073-01-B1-3-4_result/03.AlphaDiversity/Alpha_div/shannon/shannon.pdf]

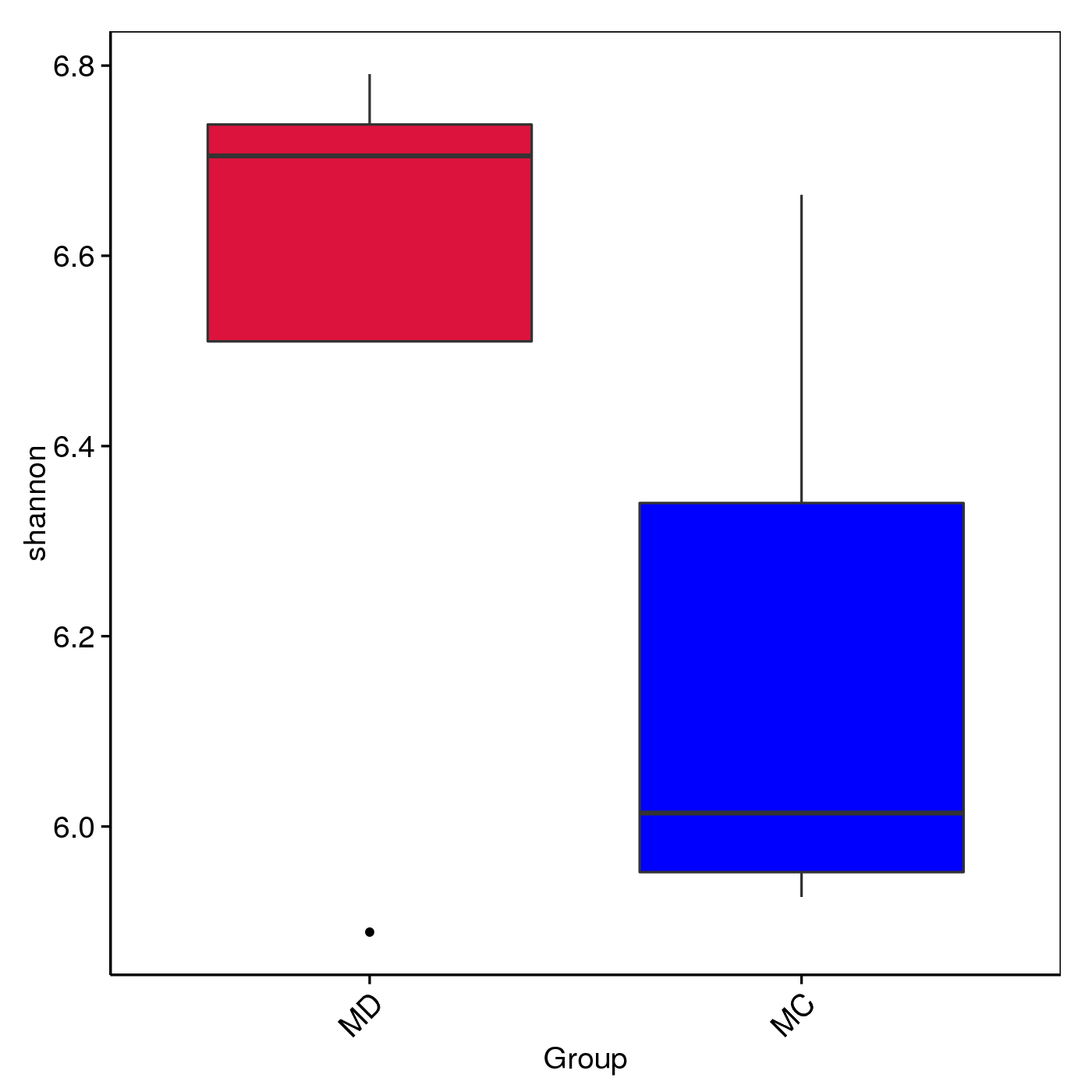

Supplement: Supplementary file 1 [file Data_Sheet_1.zip › P101SC18090073-01-B1-3-4_result/03.AlphaDiversity/Alpha_div/shannon/shannon.png]

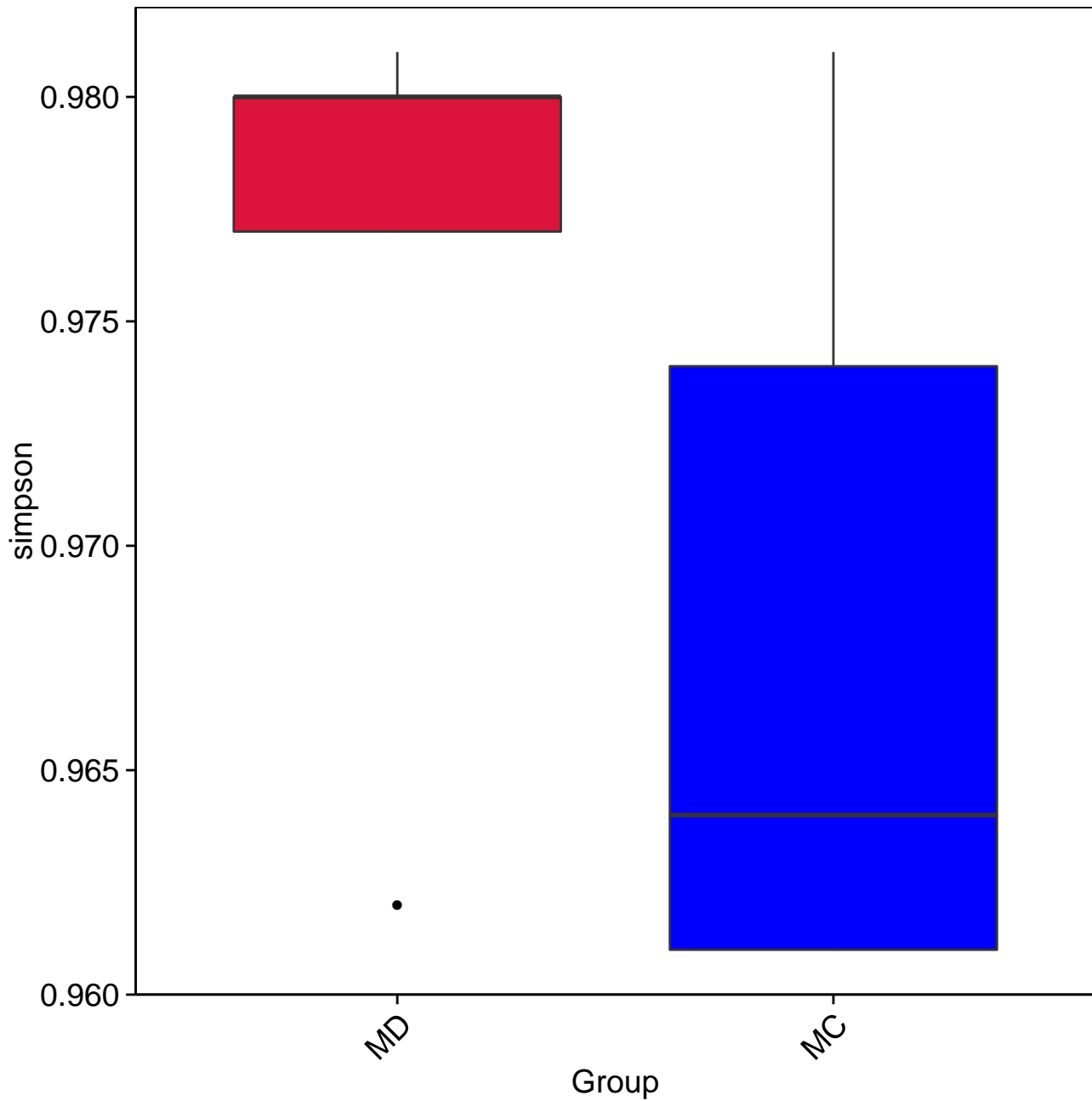

Supplement: Supplementary file 1 [file Data_Sheet_1.zip › P101SC18090073-01-B1-3-4_result/03.AlphaDiversity/Alpha_div/simpson/simpson.pdf]

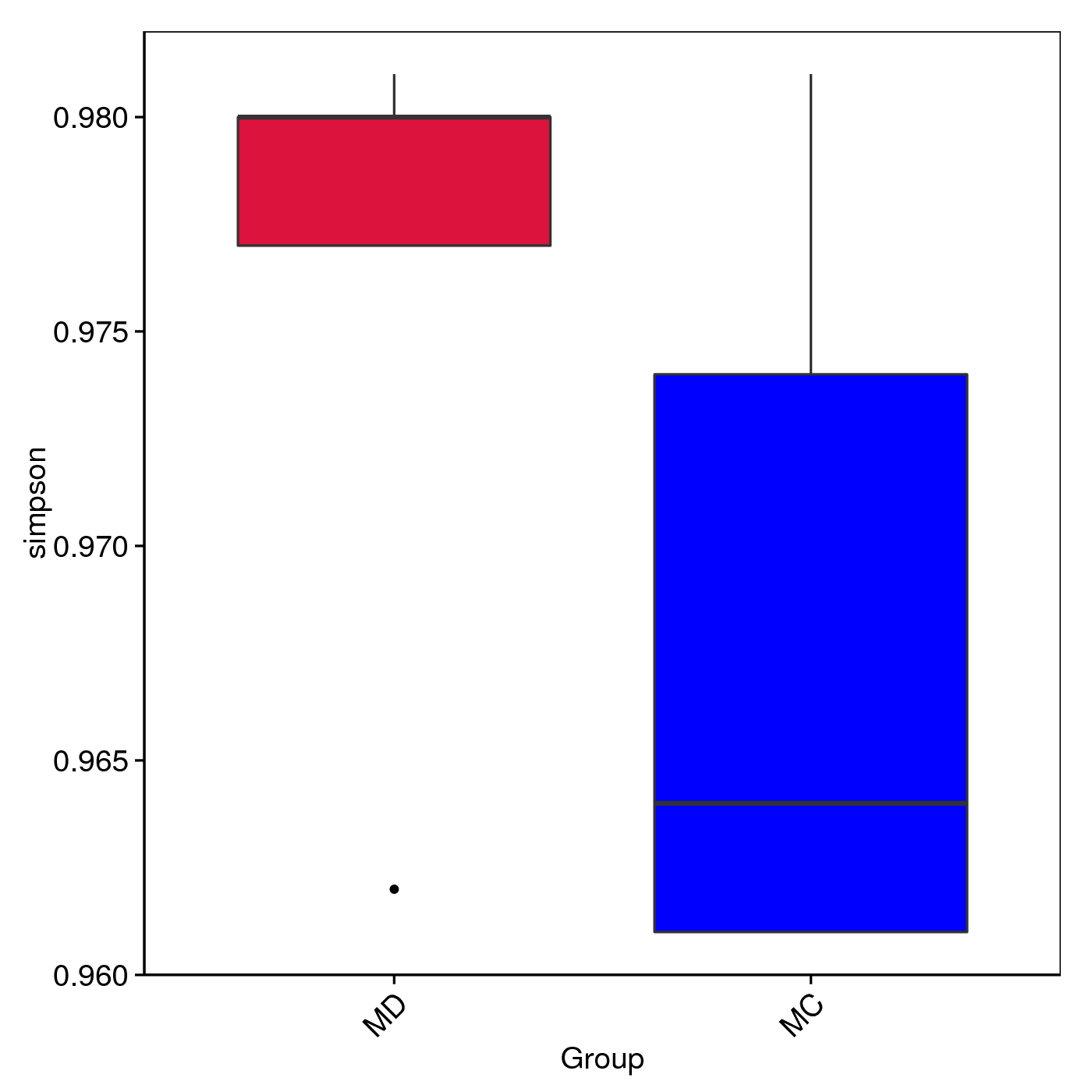

Supplement: Supplementary file 1 [file Data_Sheet_1.zip › P101SC18090073-01-B1-3-4_result/03.AlphaDiversity/Alpha_div/simpson/simpson.png]

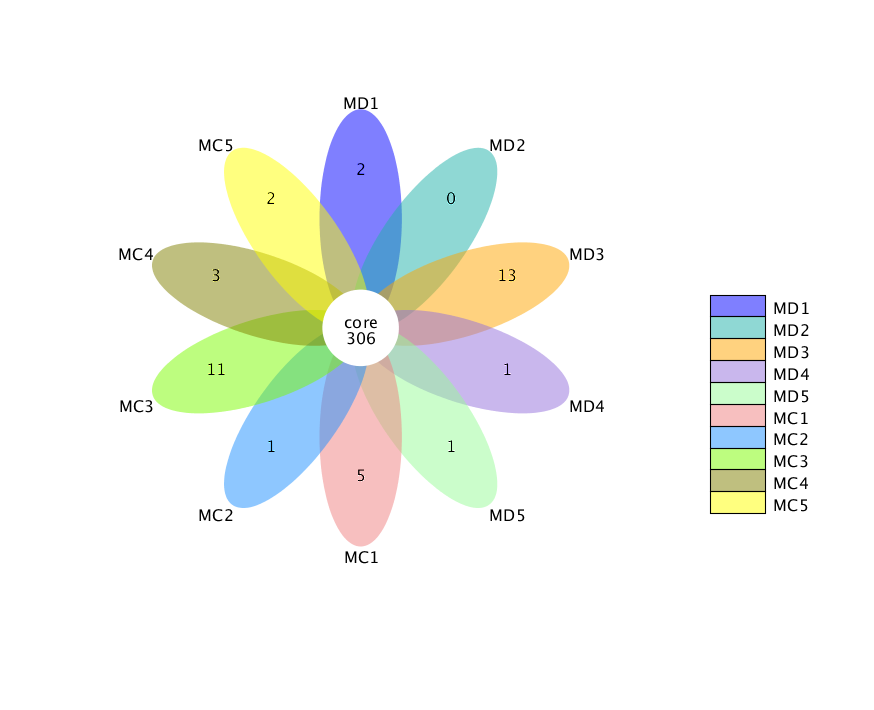

Supplement: Supplementary file 1 [file Data_Sheet_1.zip › P101SC18090073-01-B1-3-4_result/03.AlphaDiversity/Flower_figure/1_MD1_MD2_MD3_MD4_MD5_MC1_MC2_MC3_MC4_MC5.flower.png]

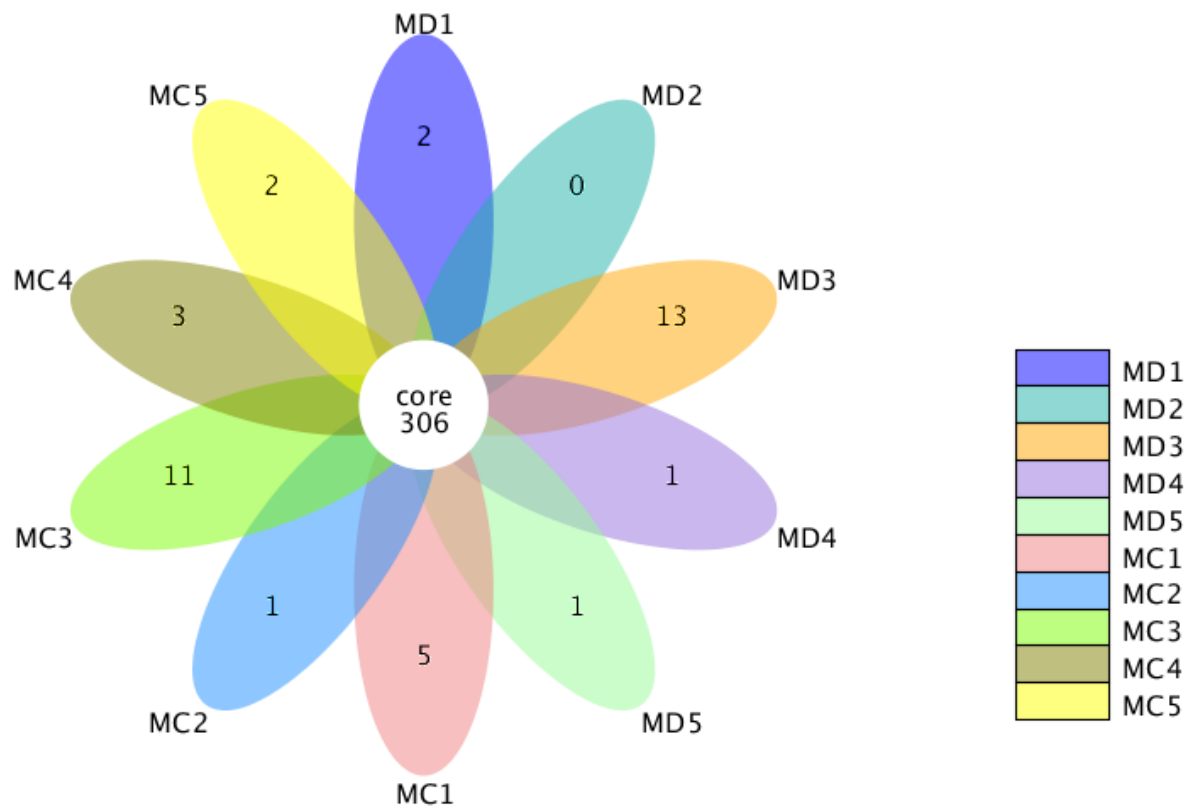

Supplement: Supplementary file 1 [file Data_Sheet_1.zip › P101SC18090073-01-B1-3-4_result/03.AlphaDiversity/Flower_figure/flower_display.pdf]

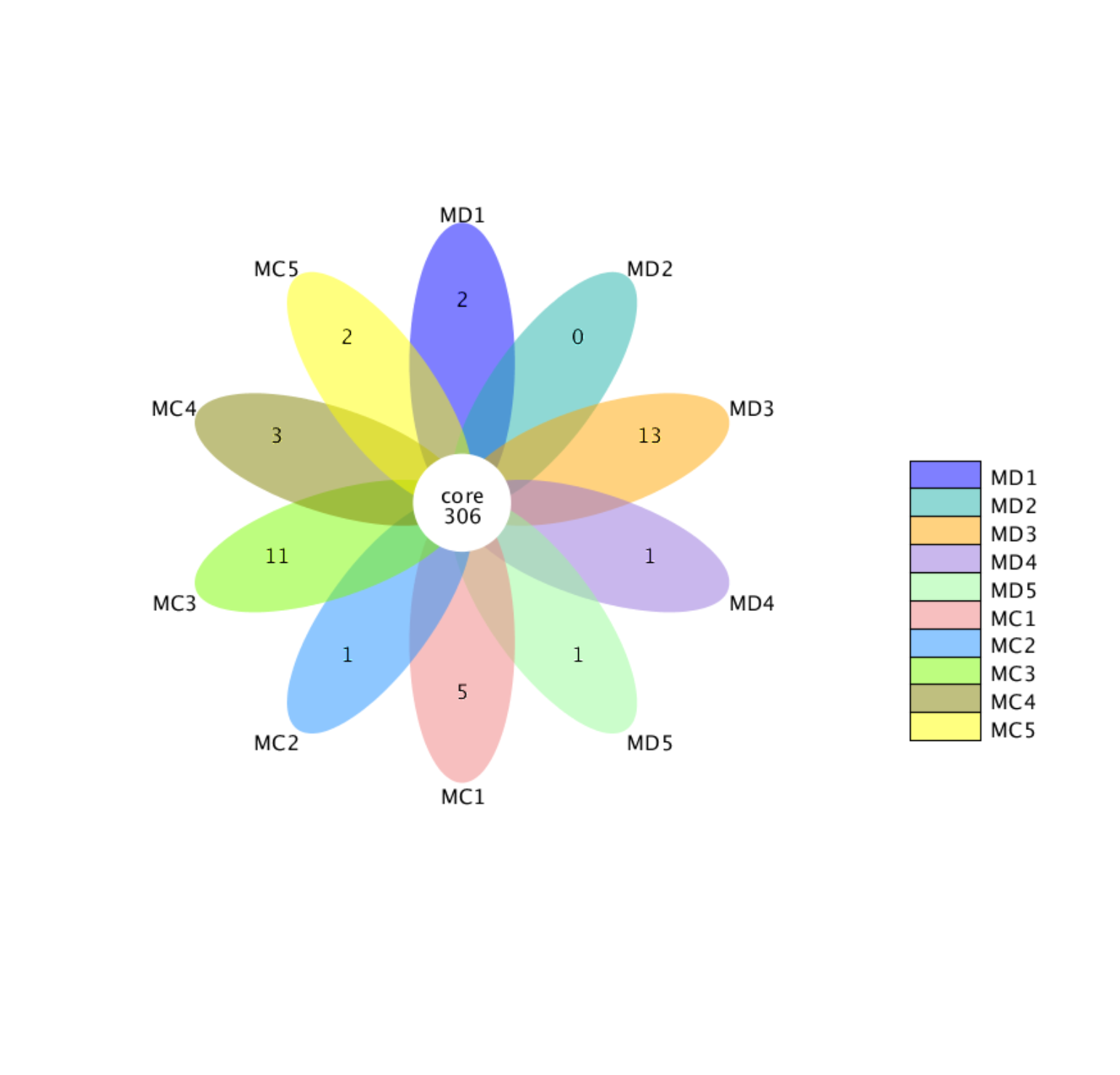

Supplement: Supplementary file 1 [file Data_Sheet_1.zip › P101SC18090073-01-B1-3-4_result/03.AlphaDiversity/Flower_figure/flower_display.png]

Observed species

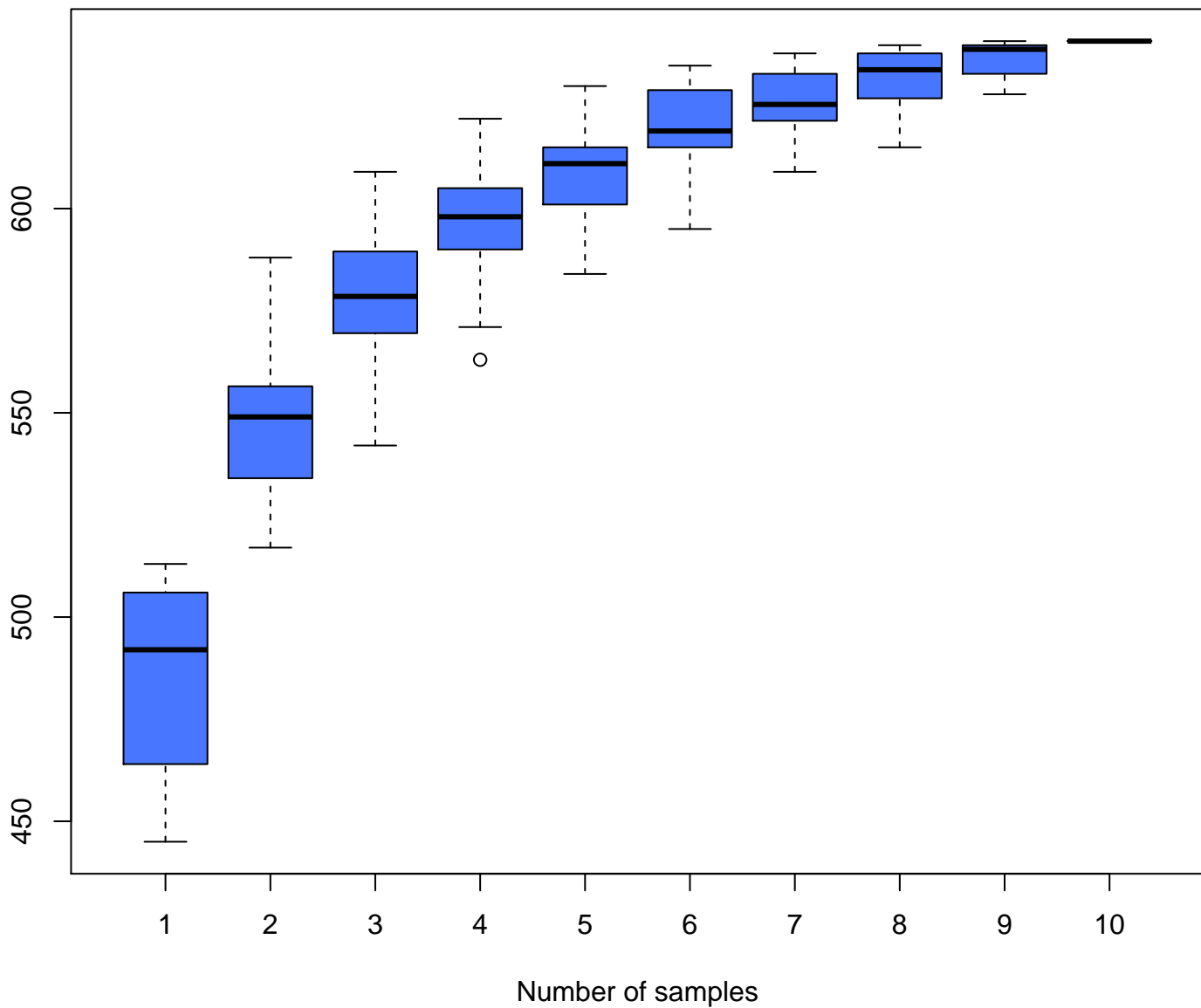

Supplement: Supplementary file 1 [file Data_Sheet_1.zip › P101SC18090073-01-B1-3-4_result/03.AlphaDiversity/Specaccum/specaccum_test.pdf]

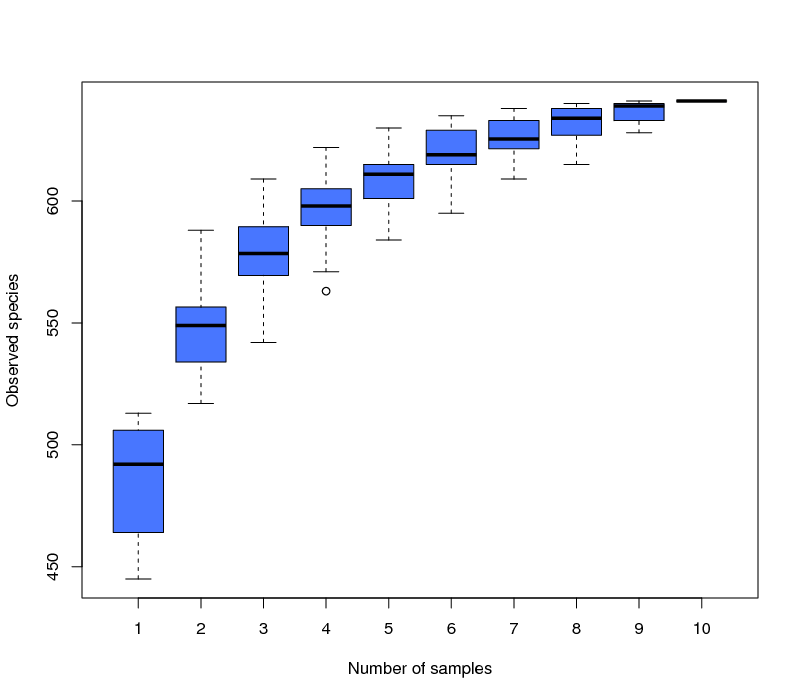

Supplement: Supplementary file 1 [file Data_Sheet_1.zip › P101SC18090073-01-B1-3-4_result/03.AlphaDiversity/Specaccum/specaccum_test.png]

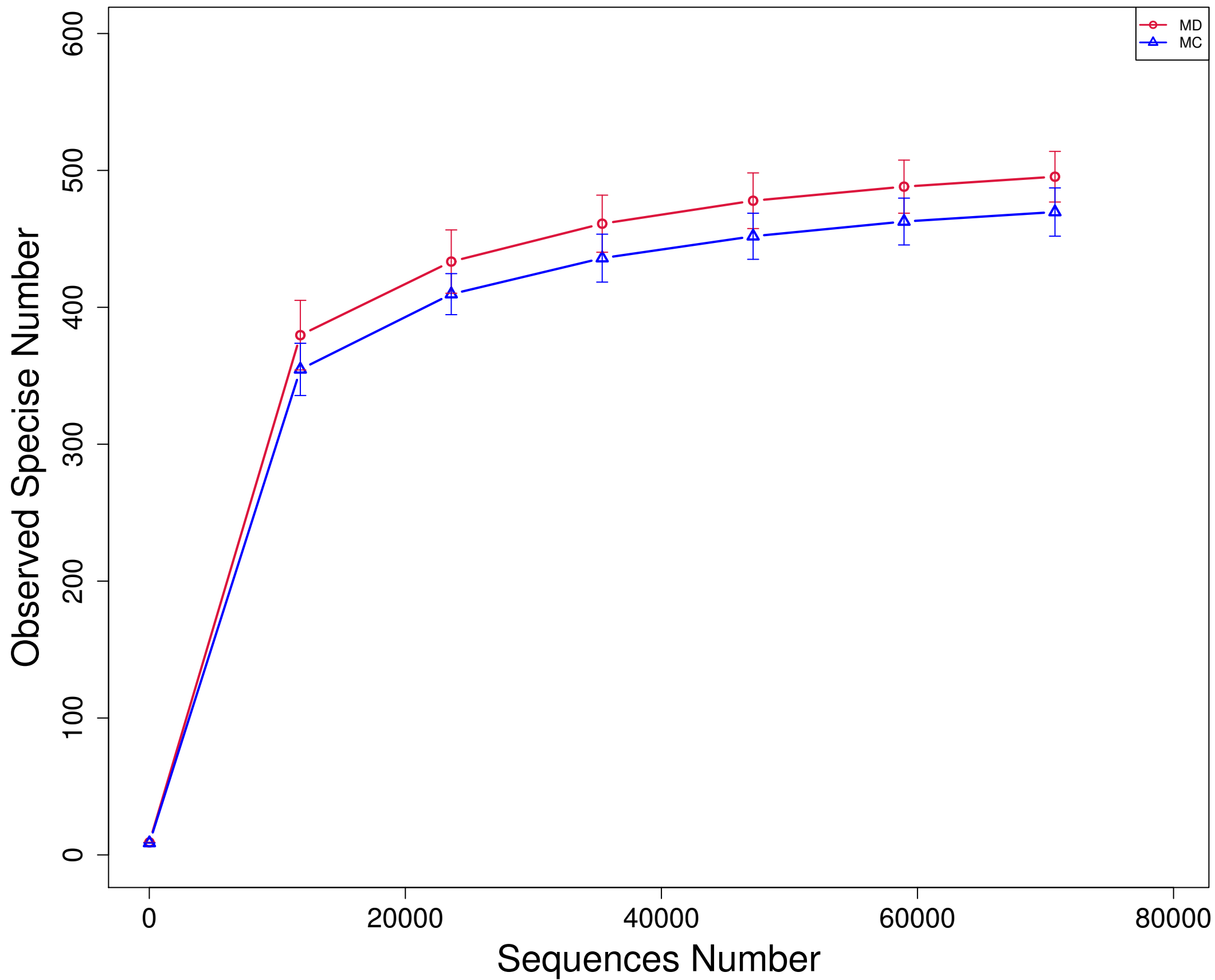

Supplement: Supplementary file 1 [file Data_Sheet_1.zip › P101SC18090073-01-B1-3-4_result/03.AlphaDiversity/group_observed_species.pdf]

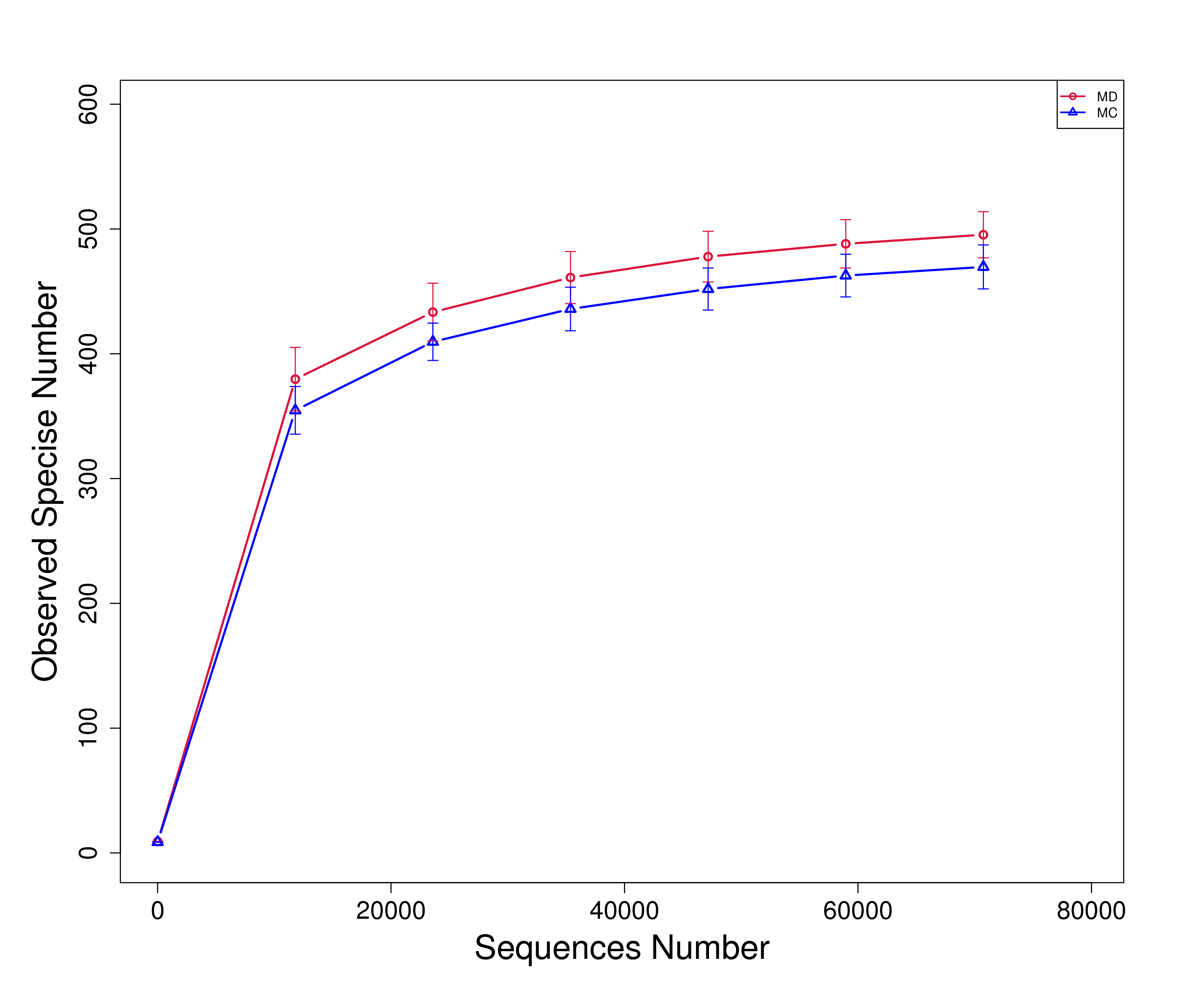

Supplement: Supplementary file 1 [file Data_Sheet_1.zip › P101SC18090073-01-B1-3-4_result/03.AlphaDiversity/group_observed_species.png]

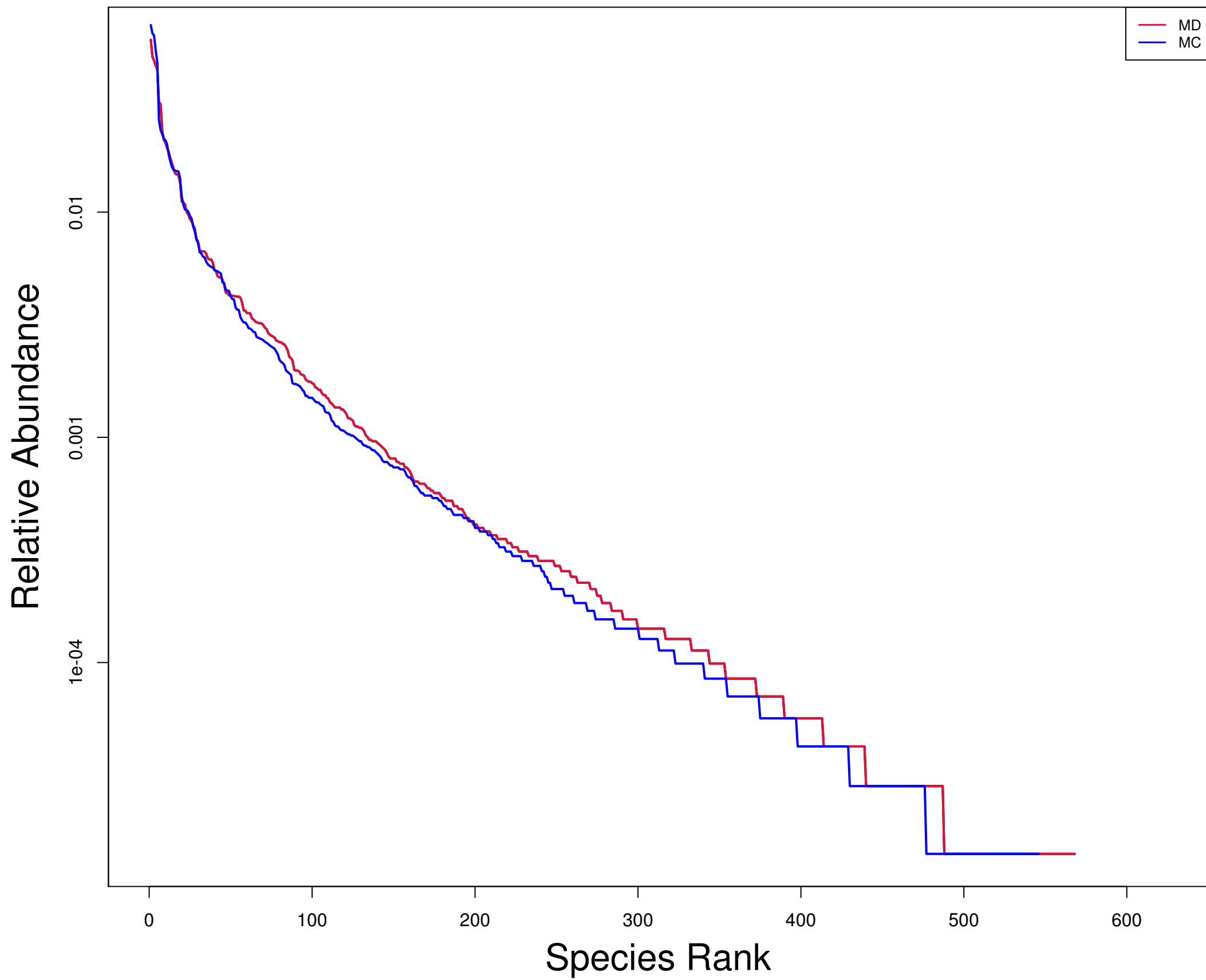

Supplement: Supplementary file 1 [file Data_Sheet_1.zip › P101SC18090073-01-B1-3-4_result/03.AlphaDiversity/group_rank_abundance.pdf]

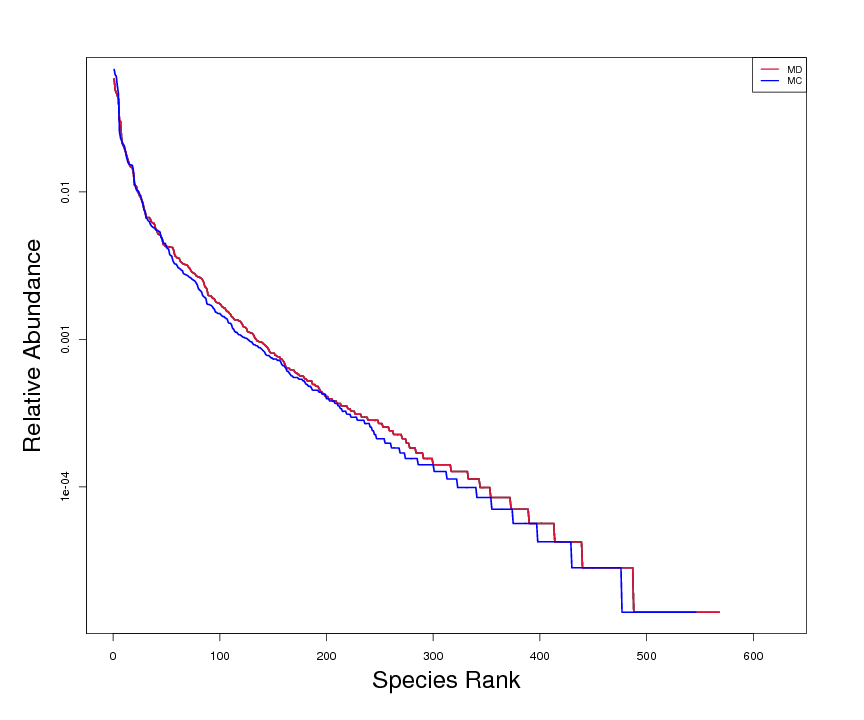

Supplement: Supplementary file 1 [file Data_Sheet_1.zip › P101SC18090073-01-B1-3-4_result/03.AlphaDiversity/group_rank_abundance.png]

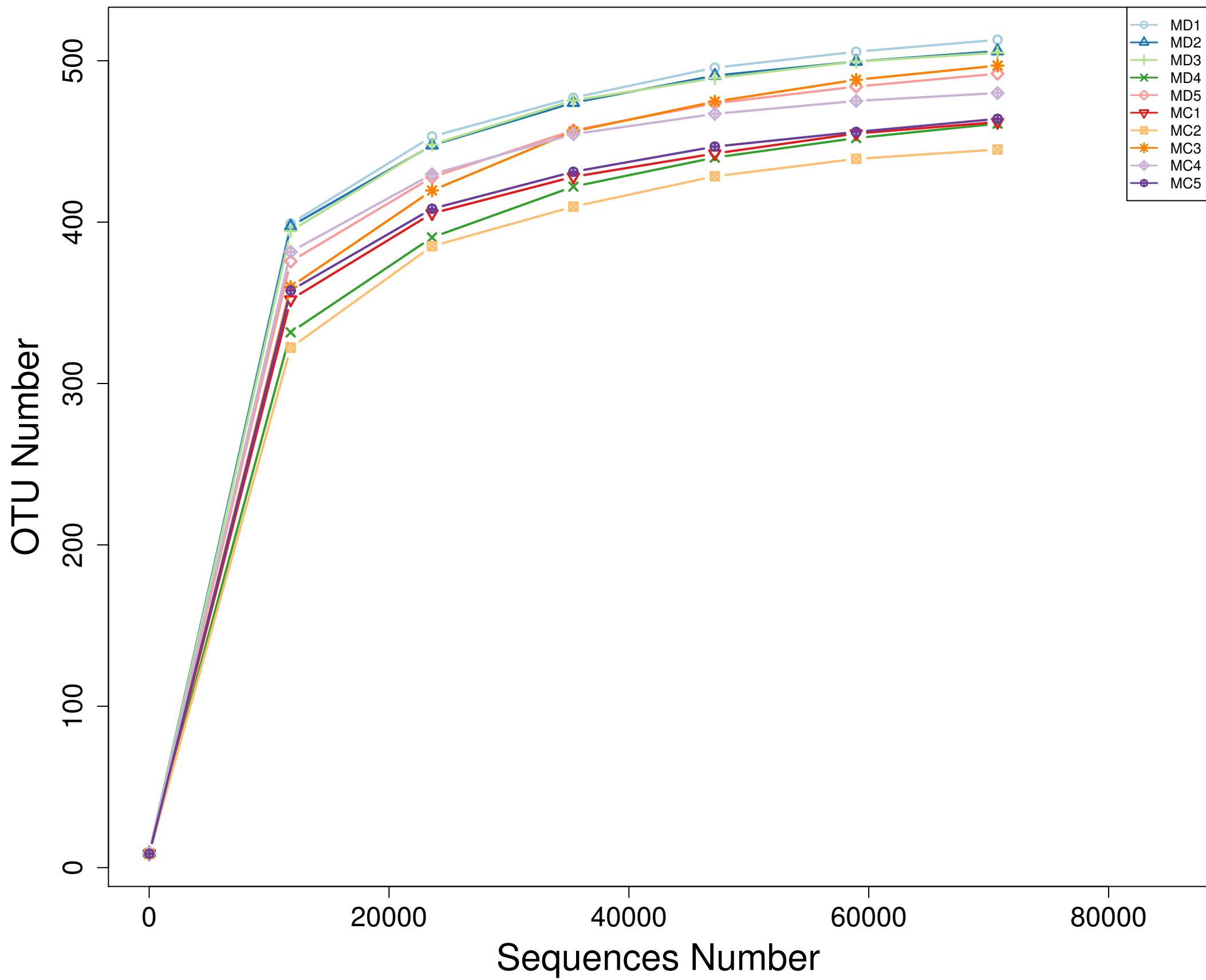

Supplement: Supplementary file 1 [file Data_Sheet_1.zip › P101SC18090073-01-B1-3-4_result/03.AlphaDiversity/observed_species.pdf]

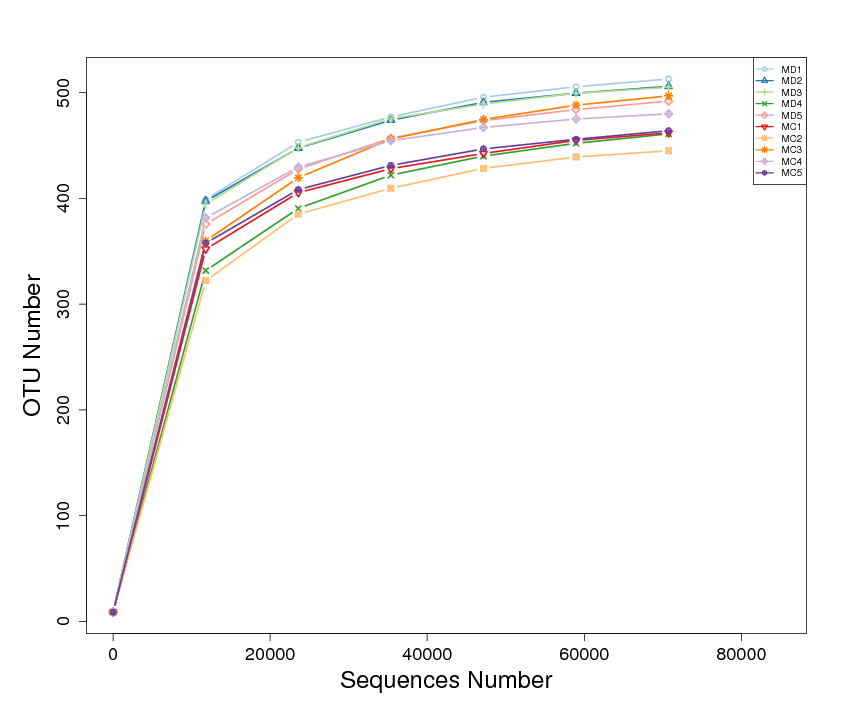

Supplement: Supplementary file 1 [file Data_Sheet_1.zip › P101SC18090073-01-B1-3-4_result/03.AlphaDiversity/observed_species.png]

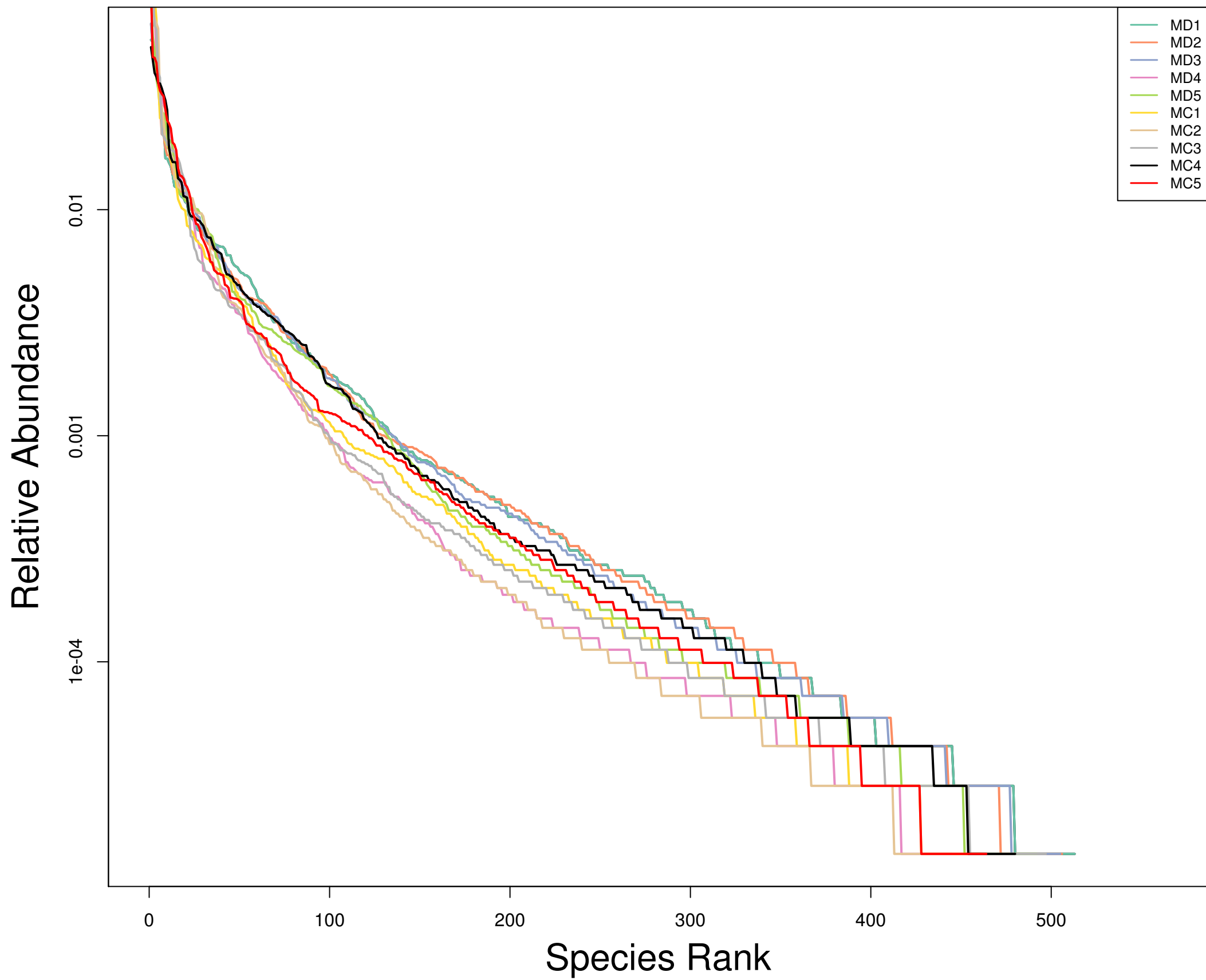

Supplement: Supplementary file 1 [file Data_Sheet_1.zip › P101SC18090073-01-B1-3-4_result/03.AlphaDiversity/rank_abundance.pdf]

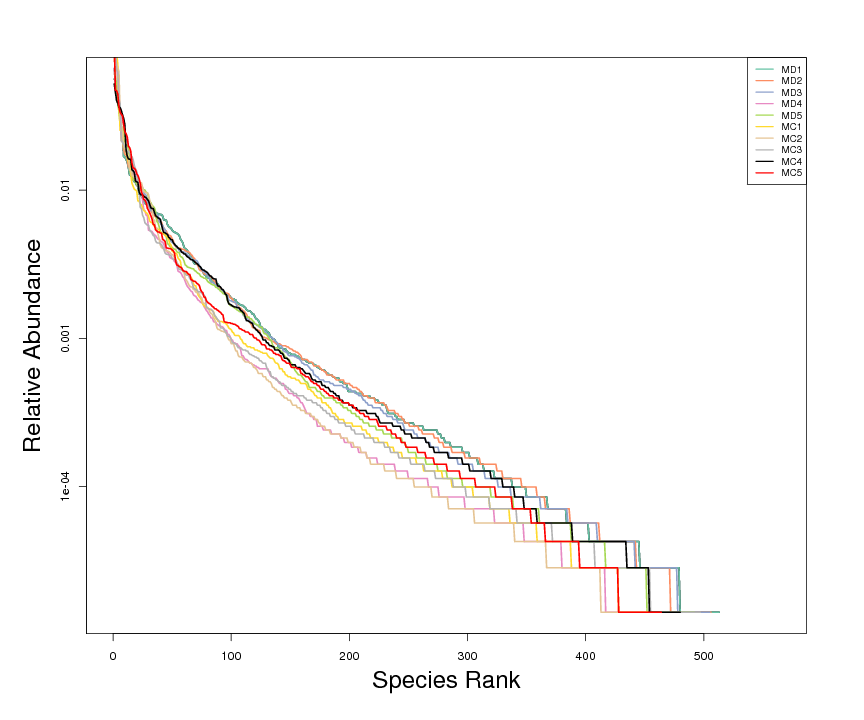

Supplement: Supplementary file 1 [file Data_Sheet_1.zip › P101SC18090073-01-B1-3-4_result/03.AlphaDiversity/rank_abundance.png]

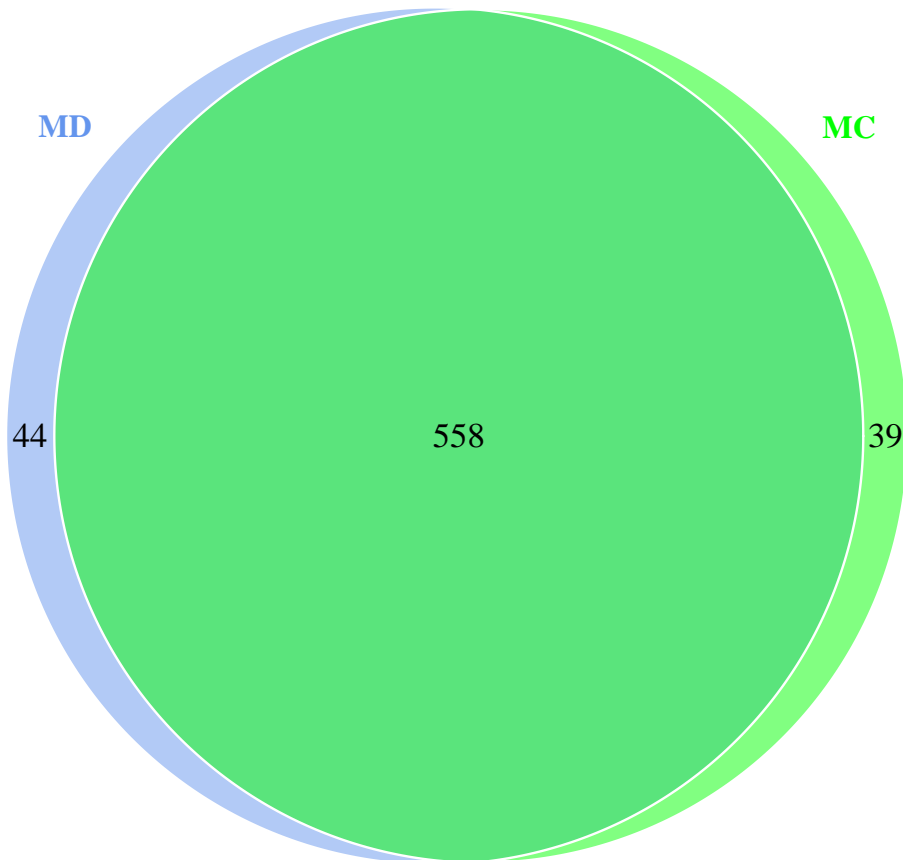

Supplement: Supplementary file 1 [file Data_Sheet_1.zip › P101SC18090073-01-B1-3-4_result/03.AlphaDiversity/venn_group/1_MD_MCvenn.pdf]

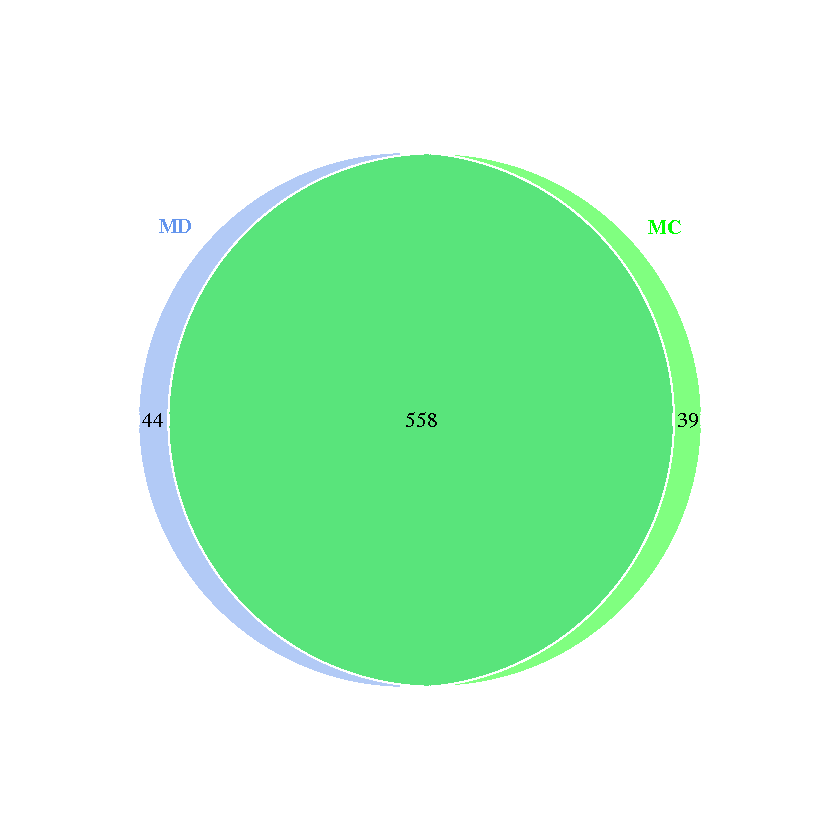

Supplement: Supplementary file 1 [file Data_Sheet_1.zip › P101SC18090073-01-B1-3-4_result/03.AlphaDiversity/venn_group/1_MD_MCvenn.png]
